# Study protocol for a randomized clinical trial to assess 7 *versus* 14-days of treatment for *Pseudomonas aeruginosa* bloodstream infections (SHORTEN-2 trial)

**DOI:** 10.1371/journal.pone.0277333

**Published:** 2022-12-22

**Authors:** José Molina, Clara María Rosso-Fernández, Enrique Montero-Mateos, José Ramón Paño-Pardo, María Solla, Ana Belén Guisado-Gil, Rocío Álvarez-Marín, María Eugenia Pachón-Ibáñez, Adelina Gimeno, Guillermo Martín-Gutiérrez, José Antonio Lepe, José Miguel Cisneros, on behalf of the SHORTEN-2 trial team

**Affiliations:** 1 Unit of Infectious Diseases, Microbiology and Parasitology, Virgen del Rocío University Hospital, Seville, Spain; 2 Institute of Biomedicine of Seville (IBiS), Virgen del Rocío University Hospital/CSIC/University of Seville, Seville, Spain; 3 CIBER de Enfermedades Infecciosas, Instituto de Salud Carlos III, Madrid, Spain; 4 Unidad de Investigación Clínica y Ensayos Clínicos (CTU), Hospital Virgen del Rocío, Sevilla, Spain; 5 Department of Pathology and Institute of Biomedical Research of Salamanca (IBSAL), University Hospital of Salamanca, Salamanca, Spain; 6 Department of Infectious Diseases, Hospital Clínico Universitario Lozano Blesa, Zaragoza, Spain; 7 Instituto de Investigación Sanitaria Aragón (IIS Aragón), Zaragoza, Spain; 8 Department of Pharmacy, Virgen del Rocío University Hospital, Seville, Spain; GERMANY

## Abstract

**Background:**

Research priorities in Antimicrobial Stewardship (AMS) have rapidly evolved in the last decade. The need for a more efficient use of antimicrobials have fueled plenty of studies to define the optimal duration for antibiotic treatments, and yet, there still are large areas of uncertainty in common clinical scenarios. *Pseudomonas aeruginosa* has been pointed as a priority for clinical research, but it has been unattended by most randomized trials tackling the effectiveness of short treatments. The study protocol of the SHORTEN-2 trial is presented as a practical example of new ways to approach common obstacles for clinical research in AMS.

**Objective:**

To determine whether a 7-day course of antibiotics is superior to 14-day schemes for treating bloodstream infections by *P*. *aeruginosa* (BSI-PA).

**Methods:**

A superiority, open-label, randomized controlled trial will be performed across 30 Spanish hospitals. Adult patients with uncomplicated BSI-PA will be randomized to receive a 7 *versus* 14-day course of any active antibiotic. The primary endpoint will be the probability for the 7-day group of achieving better outcomes than the control group, assessing altogether clinical effectiveness, severe adverse events, and antibiotic exposure through a DOOR/RADAR analysis. Main secondary endpoints include treatment failure, BSI-PA relapses, and mortality. A superiority design was set for the primary endpoint and non-inferiority for treatment failure, resulting in a sample size of 304 patients.

**Conclusions:**

SHORTEN-2 trial aligns with some of the priorities for clinical research in AMS. The implementation of several methodological innovations allowed overcoming common obstacles, like feasible sample sizes or measuring the clinical impact and unintended effects.

**Trial registration:**

EudraCt: 2021-003847-10; ClinicalTrials.gov: NCT05210439.

## Introduction

The rapid spread of bacterial resistance has become a priority Public Health problem worldwide [1], and urgent actions are needed to reverse this global trend, which is estimated to produce more than 10 million deaths a year by 2050 [2]. *Pseudomonas aeruginosa* infections have been pointed by the World Health Organization as a critical priority for clinical research [3], and yet, there still are large areas of uncertainty regarding its usual clinical management, like optimal treatment duration [4] or the need of combined therapies [5].

One of the main vectors for the selection of bacterial resistance is the prolonged duration of antibiotic treatments [6]. Therefore, generating good-quality evidence to allow a more efficient use of antibiotics has been a priority in the clinical research tackling potential responses against resistant bacteria [7, 8]. Hitherto, three randomized trials have confirmed the non-inferiority of 7-day courses of antibiotics compared to 14-day schemes for non-complicated bacteremic infections produced by Gram-negative bacilli [9–11]. Nevertheless, only one of them included a limited number of cases of bloodstream infections produced by *P*. *aeruginosa* (BSI-PA), and thus the question on the efficacy of short treatments remains open for this microorganism, whose ability to produce relapsing infections and breakthrough antibiotic resistance is superior to other Gram-negative bacteria [12, 13].

Although clinical trials would be the preferential design to define the optimal duration of treatments, some limitations have been observed when used to define the best therapeutic strategy in the setting of antimicrobial stewardship (AMS). Interventions pursuing reduced durations may be associated with less effectiveness in certain circumstances [14, 15], while others targeting enhanced effectiveness may entail increased adverse events (either toxicity or bacterial resistance) [16, 17]. Traditional assessment of individual outcomes may be insufficient for AMS, where the benefits of reducing antibiotic exposure of patients need to be properly balanced with effectiveness. The Desirability of Outcome Ranking and Response Adjusted for Days of Antibiotic Risk (DOOR/RADAR) analyses developed by Evans *et al*. [18] allow for an integrated assessment of several endpoints in clinical trials (efficacy, safety and antibiotic exposure), and have been proposed as a methodological innovation which could enhance clinical research in AMS [19]. However, few trials have implemented it hitherto [9, 20, 21].

In this manuscript, we present the study protocol of a randomized clinical trial designed to prove the efficacy and the safety of 7-day schemes for treating patients with non-complicated BSI-PA. Methodological innovations implemented to enhance the design, conduction and outcome assessment of the trial are also discussed.

## Material and methods

### Objective

To determine whether 7-day courses of antibiotics are superior to traditional 14-day schemes for BSI-PA, by assessing altogether its clinical effectiveness and its potential to reduce antibiotic exposure and severe adverse events.

### Design

The SHORTEN-2 trial is an investigator-driven, open-label, randomized controlled trial in phase IV.

### Setting

The trial will be developed across 30 Spanish hospitals (S1 File).

### Eligibility

Adult patients with BSI-PA will be eligible. Main exclusion criteria include: (a) source of the bacteremia not properly controlled at least 72h before randomization; (b) BSI-PA due to an infection requiring prolonged treatment (e.g., necrotizing pneumonia, prostatitis, bone and joint infections, etc.); (c) neutropenia <500 cells/mm3 at randomization; (d) bacteremic pneumonia in severely immunocompromised hosts; (e) Serious burning; (f) BSI-PA by strains resistant to all betalactams *and* fluorquinolones; (g) another episode of BSI-PA in the previous 90 days; (h) polymicrobial bacteraemia; (i) patients on palliative care or with a survival expectancy < 48h; (j) pregnancy.

### Recruitment

Blood samples of patients with suspected bacteremia will be processed at local Microbiology laboratories according to current EUCAST recommendations [22]. Participating hospitals develop as part of their routine clinical practice daily meetings between Microbiologists and Infectious Diseases physicians where all positive blood cultures in the center are reported. Potential participants will be identified at these meetings. All centers named both an Infectious Diseases physician and a Microbiologist as local responsible researchers for the trial to facilitate screening and follow-up tasks. Recruitment will be reinforced with periodical newsletters and meetings, and different social media actions through a Twitter^®^ account created *ad hoc* (@shorten2trial).

### Intervention

Patients will be allocated to receive either 7 days or 14 days of any active antibiotic treatment since the date of the last positive blood culture. Treatments will be stopped at the scheduled point as far as patients have remained apyretic and with no signs or symptoms of infection in the preceding 72 hours. If this condition is not confirmed at this point, patients will be reassessed each 48 to 72 hours until these requirements are fulfilled. Treatment dosing or infusion modalities will be optimized according to the latest EUCAST recommendations [22] after randomization if not previously performed. Other clinical decisions (de-escalation, switch to oral route, hospital discharge, etc.) will be accomplished according to the criterion of the clinician in charge of the patient. Antibiotic treatments will be resumed whenever necessary if an unfavorable course is suspected. The administration of the trial treatment in outpatient antimicrobial therapy programs, or patient management on a fully ambulatory-care basis are also permitted if considered appropriate. Definitions regarding antibiotic treatments are detailed in S2 File, Section A.

### Outcomes

The primary outcome will be the probability of patients in the 7-day arm of achieving better results than the 14-day assessing altogether clinical outcomes, risk of adverse events and antibiotic exposure through a DOOR/RADAR analysis. The DOOR ordinal scale for clinical outcomes will be defined as follows: (1) clinical cure without incidences; (2) clinical cure with a proven or probable relapse; (3) clinical cure with a severe adverse event; (4) not cured; (5) death. Patients in the same clinical outcome category will be ranked according to the number of days of antibiotic treatment received for any cause at the test of cure visit (day +30 after treatment cessation). The probability for patients in the 7-day arm of achieving a better result (a lower DOOR/RADAR score) than patients in the 14-day arm will be calculated.

Secondary outcomes include treatment failure, a DOOR analysis (without RADAR), all-cause mortality, proven, probable, or possible relapses, new episodes of BSI-PA for any cause, relapse of fever for any cause, superinfections, severe adverse events, number of days free of antibiotic treatment, number of days of hospital stay, and recovery of basal functional status. All outcomes will be assessed at the test of cure visit (day +30 after treatment cessation) and at the end of follow-up visit (day +90 from the date of the first positive blood culture). Definitions for all outcomes can be found in S2 File, Section C.

### Follow-up

**Fig 1:**
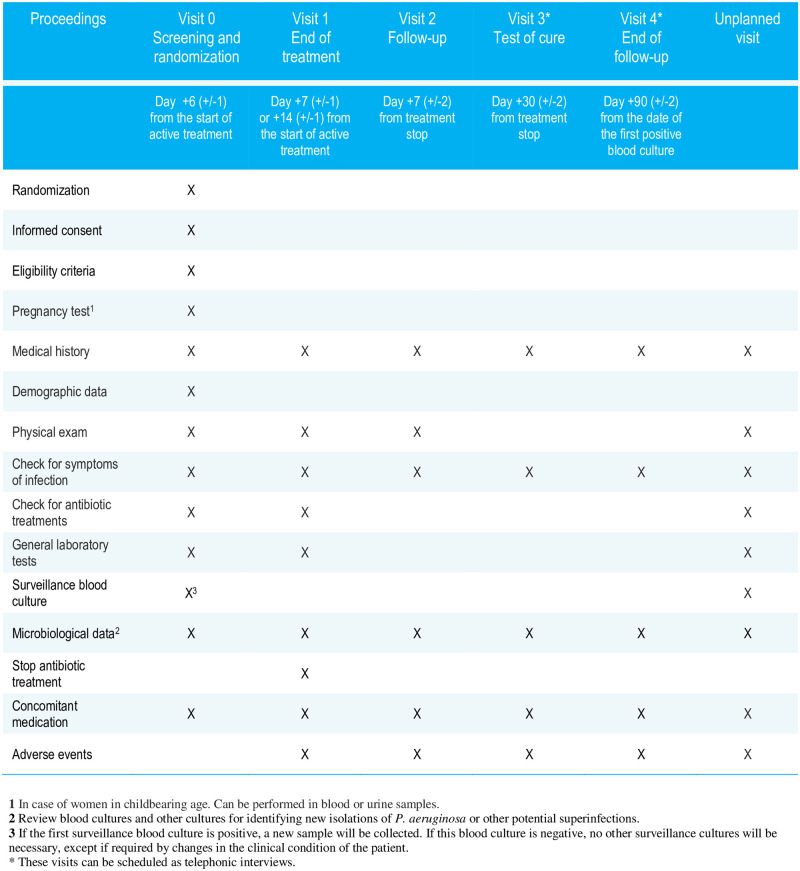


**Fig 2:**
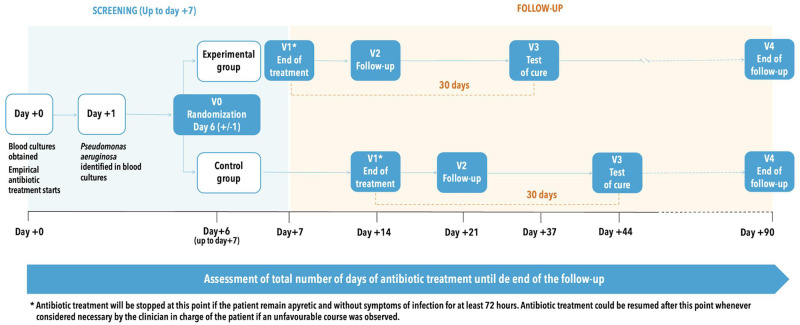


Visits and their scheduled proceedings are showed in Fig 1. The follow-up is detailed in Fig 2.

### Randomization

Randomization will be performed on day 6 (+/-24h) since the beginning of the first active antibiotic treatment. Simple randomization will be performed in a 1:1 ratio, stratified by site and by source of the BSI-PA (pneumonia or non-pneumonia sources) through a predesigned randomization list. The process will be centralized in the coordinating center and performed online through an automatic system integrated in the electronic case report form (eCRF). The randomization list will be computer-generated (Epidat 4.0). Only after the eCRF is fulfilled with inclusion criteria, the system will provide patient allocation. The Information Technology department responsible for the eCRF and the Clinical Trials Unit (CTU-HUVR) will be the only custodians of the randomization list.

### Blinding

Considering the number of antibiotics involved, an open-label design was chosen for pragmatic reasons. To avoid potential biases, precise definitions and allocation algorithms were incorporated in the eCRF to ensure an accurate assessment of primary and secondary endpoints (S2 File, Sections C-E). Additionally, primary outcomes will be confirmed by a blinded panel of experts, external to the research team. Finally, statistical analyses of the results will be performed on a dataset blinded for group allocation.

### Data collection, management, and monitoring

This is an investigator-driven trial for which CTU-HUVR has delegated sponsor functions on behalf of the Andalusian Public Foundation for the Management of Health Research of Seville (FISEVI). CTU-HUVR leads the coordination of legal and administrative procedures to get sites prepared for their participation in the study and is responsible for data revision and source verification. Access to eCRF designed for the study is pre-approved with specific attributions for database modification only possible for accredited investigators. Workers from the Spanish Clinical research Network (SCReN) will receive authorization for checking the information to ensure the accuracy of data. Principles of Good clinical Practice rules (EMA/CHMP/ICH/135/1995) will be applied on the performance of follow up and monitoring of the study.

### Statistical plan and interim analysis

All analyses will be performed on intention-to-treat and per-protocol populations. A superiority hypothesis was set for the primary endpoint. To prove it, we will determine the probability of a better DOOR/RADAR outcome in the 7-day arm and its 95% confidence interval (CI), and it will be demonstrated if the lower bound of the CI is above 60%. A non-inferiority hypothesis was set for the secondary endpoint of treatment failure, with a non-inferiority margin of 7.5%. All CIs will be calculated using the Newcombe-Wilson method. Prespecified subgroup and sensitivity analysis will be done. Multiple imputation will be used if missing data exceed the 5% and the necessary conditions for such procedure are met. All analyses will be performed using R software (version 4.1). An interim analysis will be performed when 40% of the sample is recruited. The potential futility of the trial will be assessed through the conditional power (CP) for the safety variable of non-inferiority. A CP of <15% is considered to make it unlikely to demonstrate the efficacy of the intervention. A complete description of the statistical plan can be found in S3 File.

### Sample size

Sample size was calculated for both the superiority and non-inferiority endpoints. For the primary endpoint, assuming a superiority margin of 60%, a two-sided error α = 0.05, and a power of 80%, 262 patients will be needed. The sample size for the secondary outcome was based on Bae *et al*. [4], which reported a therapeutic failure of 15.6% and 11.3% for patients receiving prolonged or short treatments. 262 patients will be required for a non-inferiority margin of 7.5%, a power of 80% and a one-sided α-error of 0.025. Assuming a 5% loss and a non-adherence of 5%, a final sample size of 304 was set.

### Safety and adverse event reporting

Severe adverse events (SAE) will be reported to the Pharmacovigilance department of the coordinating center within 24 hours after their detection. Relationship with the study medications will be evaluated considering the opinion of the personal notifying the adverse event and the safety information available for each active antibiotic permitted. Meddra (Medical Dictionary for regulatory Activities) dictionary will be used to codify SAEs, and reports on safety will be sent to the Regulatory Authority (RA) timely. A periodic reconciliation on safety data will be performed to ensure all the eCRF gathered information is properly communicated to the competent authorities.

### Ethical and regulatory considerations

The trial was approved by the Seville Provincial Committee on Ethics in Research on Medical Products on January 10^th^ 2022 (minutes number 22/2021) for the 30 participant sites. The Spanish Regulatory Authority (AEMPS) granted its authorization on January 12th, 2022, with classification of “Low Intervention Risk” due to the pragmatic design of the trial. Principles of the Declaration of Helsinki are considered, and all patients must sign an informed consent form before any procedure is done. Follow-up reports of the situation of recruitment and data safety updated report will be sent to RA and EC according to local legislation. Data protection legislation is considered for any data treatment along the study. Access to study data will be restricted to investigators until the database is completely locked, analyzed, and published provided it is required by RA. Results will be published according to CONSORT standards [23].

### Timeline

The study is planned to start recruitment in the first quarter of 2022, and it is anticipated to be completed in the first quarter of 2025. The expected time for publication is the end of 2025.

## Discussion

Research priorities in the area of knowledge of AMS have rapidly evolved, paralleling an exponential increase in the number of publications in the last decade [24]. Different experts have converged into a series of unmet needs, mostly related to the demonstration of the clinical benefits of AMS strategies or the best ways to implement them (Table 1) [7, 8, 19, 25–27]. Whilst achieving these more ambitious goals can be challenging, several actions are possible to overcome common obstacles (Table 2), many of which have been implemented in the study protocol presented in this article.

**Table 1:** Priorities in clinical research on antimicrobial stewardship [7, 8, 19, 25–2,7].

A. Generate new evidence for the most appropriate use of antimicrobials.
To optimize the use of already-available antibiotics in common infectious syndromes: duration, dosing and infusion strategies, including the use of therapeutic drug monitoring.
To optimize antibiotic treatments and prophylaxis in strategic special populations commonly excluded or poorly represented in clinical trials: immunocompromised hosts, obesity, elderly or paediatric patients, etc.
Validate the effectiveness of antibiotic-sparing strategies: monotherapy versus combined therapies, carbapenem-sparing schemes, etc.
B. Develop new designs and methodologies to prove the clinical benefits of ASPs.
Investment in infrastructures that enhance interventions and the assessment of their results both at an individual and population level.
Standardize process and outcome measures for cuasi-experimental and observational studies, in order to enable comparisons between interventions.
Prioritize designs able to avoid risk of bias in effectiveness studies: randomized controlled trials, cluster-randomized trials, cuasi-experimental studies with time series analyses.
Implementation of new statistical methods for assessing benefits and potential risks of AMS interventions: DOOR/RADAR analyses, DOOR/MAT analyses, etc.
To facilitate the conduction of pragmatic, point-of-care trials able to structure the assessment of the efficacy of those non-experimental treatments provided spontaneously in healthcare centers according to local dogmas (“pseudo-randomization”).
C. Identify the most efficient interventions for antimicrobial stewardships programs (ASP) and the best strategies for their implementation.
Comparative analyses of different interventions in terms of effectiveness, feasibility, and costs.
Assessment of rapid microbiological diagnostic programs, optimal strategies for implementation and its clinical impact.
Identify elements that support the long-term sustainability of ASPs.
Design specific interventions for non-hospital settings: primary care, emergency departments, long-term care, post-discharge treatments, etc.
Define requirements and new tools for the implementation of ASPs in settings lacking workers with an expertise in Infectious Diseases or antimicrobial therapy: human resources (specially the role of nurses), electronic tools for clinical decision support, distance training, etc.
Identify behavioral factors (social, organizational, emotional, cultural…) that have an influence on antibiotic use, and incorporate them into ASP designs.
D. Define adequate metrics for assessing the clinical impact of ASPs.
Clinical outcome measures: define and validate standardized measures for assessing the impact of ASPs on patients’ health.
Antimicrobial consumption measures: define and validate standardized measures for assessing consumption in the non-hospital setting, prescriptions at hospital discharge or defining the appropriateness of prescriptions.
Bacterial resistance measures: define indicators able to measure the risk for the development of resistance according to antibiotic selection, dose, or duration.
Process measures: define and validate process measures that enable the demonstration of causality between ASPs and clinically significant benefits.

**Table 2:** Frequent obstacles for the design of academic clinical trials in antimicrobial stewardship (AMS), and actions proposed to overcome them.

Obstacles	Potential actions
**Designing the trial**	
**Define a relevant clinical question**	Consider current research priorities (see Table 1).
	Decline hypotheses on already-answered issues.
**Define appropriate outcome measures**	Define clinical outcomes (mortality, clinical cure, hospital stay, etc.)
	Prioritize the use of standardized outcome measures to enable comparisons or pooled analyses [19, 28]
	Assess unintended effects of the studies interventions through specific outcome measures or DOOR/RADAR analyses.
**Identify factors interfering with the outcome assessed and design measures to control them**	Pre-assessment of clinical routines among participating centers
	Design variables *ad hoc* to control potential interferences
	Implement analyses for the center effect if heterogeneity is expected.
**Set a feasible sample size**	Collaborative multicentric studies integrated in research networks
	Consider DOOR/RADAR for sample size calculation
	Design pragmatic point-of care trials [27]
**Conducting the trial**	
**Limited human resources**	Integration into research networks.
	Utilization of professional networks (scientific societies)
**Continued patient recruitment**	Design multidisciplinary local teams (microbiologists and clinicians) already involved in the clinical process tackled by the study.
	Permanent communication and engagement with local teams: use social media, newsletters, online meetings, etc.
**Publishing and disseminating trial results**	
**Future dissemination of results**	Confirm that your design is compliant with CONSORT standards [23] previously to the trial prompt.
	Transparency measures: Publish the protocol trial and statistical plan in adequate platforms (clinicaltrials.gov), scientific journals, etc.
	Lean on social media for disseminating trial progress and its scientific production.

The first step in the design of a clinical trial should be to elaborate a relevant clinical question, and attending aforementioned areas of uncertainty should be prioritized (Table 1). The aim of our trial, as well as its methodology, align well with some of these proposals.

Defining relevant clinical outcomes as final endpoints, instead of process measures (like antibiotic consumption or appropriateness of prescriptions), has also been pointed as a needed step forward for AMS. Standardizing these outcome measures to allow comparison or pooled analyses across studies could undoubtedly enhance the results to achieve in future years. An international consensus on priority primary endpoints for trials tackling bloodstream infections have recently been published [28], which were used in our trial. A guide for defining outcomes for other designs can be found in the consensus document elaborated by Schweitzer *et al*. [19].

However, the assessment of individual efficacy outcomes could still be insufficient in the setting of AMS, since unintended consequences derived from these interventions are not infrequent [16, 17, 29]. This is why designs allowing for a more accurate balance between benefits and potential harms of the compared strategies have been encouraged. The DOOR/RADAR analyses implemented in our trial provides a useful approach for studies analyzing optimal treatment durations, by assessing the impact of a given intervention in two steps: first, patients are classified according to an ordinal scale of pre-defined relevant clinical outcomes, and subsequently individuals in each clinical category are ranked according to the days of antibiotic treatment received. This classification allows to identify the intervention for which patients achieve the best results, considering all variables together. However, this methodology is not exempt from limitations [24, 30]. First, composite outcomes in DOOR/RADAR analyses may mask, under certain circumstances, worse results for specific endpoints [24]. Therefore, relevant outcomes may need specific assessment. In our design, sample size was calculated to ensure the power of the trial not only for the superiority in the DOOR/RADAR endpoint, but also the non-inferiority of treatment failures. Moreover, performing these analyses *post hoc* exposes them to an increased risk of bias, since modifications in the outcome scale can lead to completely different conclusions [30]. Contrarily to most previous studies [9, 20, 21], the SHORTEN-2 trial is one of the first to include it as a predefined analysis, and as a primary endpoint.

Recruiting appropriate sample sizes for proving clinical benefits can be challenging, even for groups already integrated into research networks. The DOOR/RADAR methodology facilitates more feasible sample sizes compared to traditional non-inferiority trials [31], enabling superiority designs that may answer the need of evidence on the neat benefits of AMS interventions.

**Fig 3:**
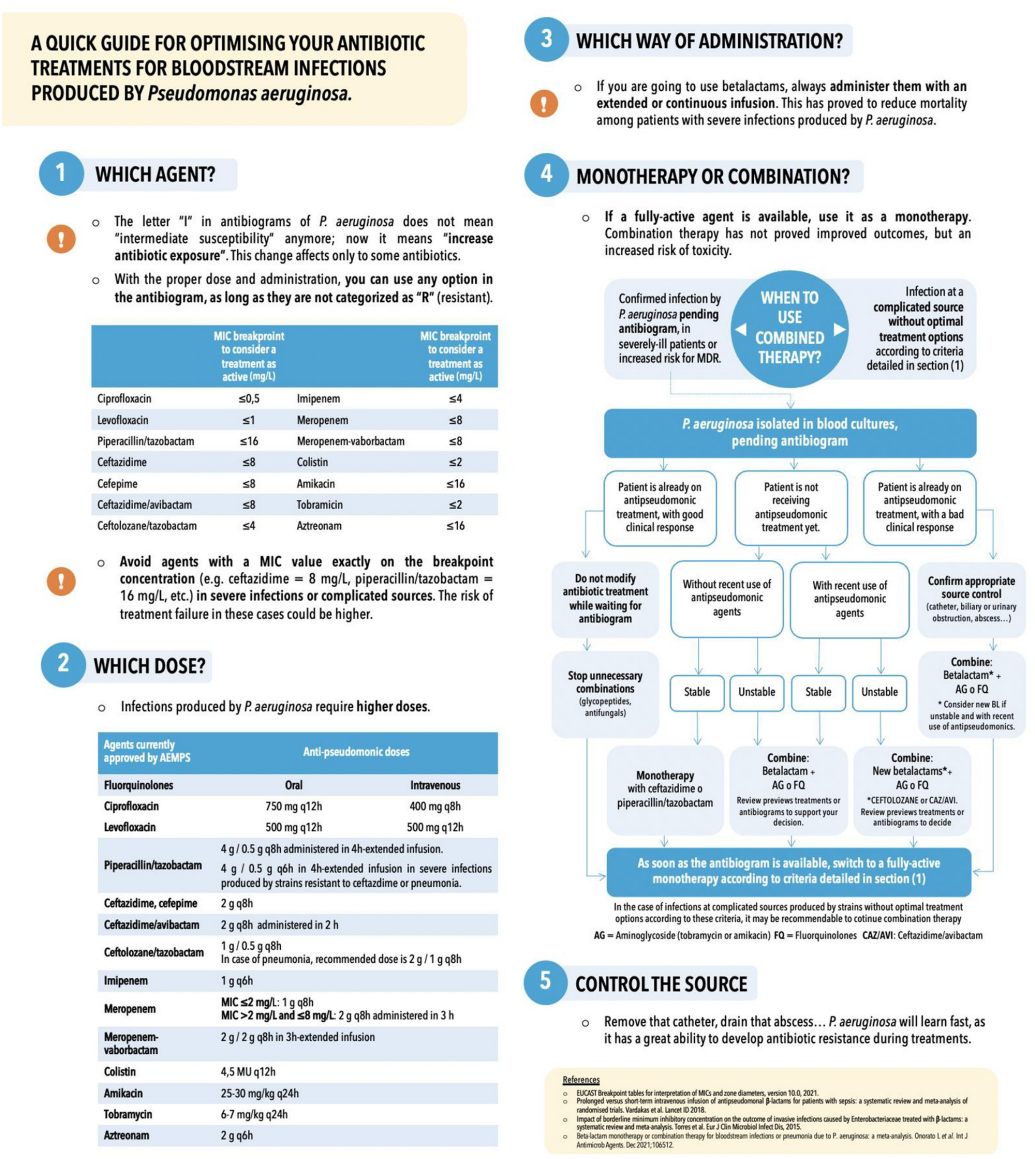


Finally, the demonstration of the clinical impact of specific aspects of antibiotic treatments (duration, dosing, routes of administration, etc.) can be complex even in the setting of randomized controlled trials, since all of them contribute to the outcomes. It will be relevant to assess clinical routines among participating centers, and to define appropriate measures *ad hoc* to control for its potential interference with the pursued endpoints. In our case, the use of an online survey allowed to gather real-time data which proved to be useful for designing the protocol and planning the kick-off visits (http://shorturl.at/bcezF, S4 File), and for defining more homogeneous clinical management among centers (Fig 3).

In conclusion, the SHORTEN-2 trial has been developed in the framework of current research priorities in AMS, and several methodological innovations have been implemented to overcome some common obstacles. The results obtained from this trial are expected to define the effectiveness of short treatments in such a priority clinical problem as BSI-PA.

## Supporting information

S1 File List of participating centers. (DOCX)

S2 File Definitions for main trial variables. (DOCX)

S3 File Statistical analysis plan. (DOCX)

S4 File Results of the survey among participating centers regarding diagnostic and clinical routines for the management of BSI-PA. (DOCX)

S5 File Full-length study protocol (English). (PDF)

S6 File Full-length study protocol (Spanish). (PDF)
