# Supplementary material for: Study protocol for a randomized clinical trial to assess 7 versus 14-days of treatment for Pseudomonas aeruginosa bloodstream infections (SHORTEN-2 trial)
Source: PLoS One. 2022 Dec 22;17(12):e0277333. doi: 10.1371/journal.pone.0277333 (PMC9778939; doi:10.1371/journal.pone.0277333)
Supplement: S1 File — (DOCX) [file pone.0277333.s001.docx]

S1 File. List of participating centers.

| Center | Location |
| --- | --- |
| **Hospital Universitario Virgen del Rocío (coordinating center)** | **Sevilla (Andalucía)** |
| Complejo Hospitalario de Jaén | Jaén (Andalucía) |
| Complexo Hospitalario Universitario A Coruña | La Coruña (Galicia) |
| Complexo Hospitalario Universitario de Vigo | Pontevedra (Galicia) |
| Hospital Clínico Universitario Lozano Blesa | Zaragoza (Aragón) |
| Hospital Costa del Sol | Málaga (Andalucía) |
| Hospital Parc Taulí | Barcelona (Cataluña) |
| Hospital Universitario Central de Asturias | Asturias (Asturias) |
| Hospital Universitario Clínico San Cecilio | Granada (Andalucía) |
| Hospital Universitario Cruces | Vizcaya (País Vasco) |
| Hospital Universitario de Bellvitge | Barcelona (Cataluña) |
| Hospital Universitario de Donostia | Guipúzcoa (País Vasco) |
| Hospital Universitario de Jerez de la Frontera | Cádiz (Andalucía) |
| Hospital Universitario de Puerto Real | Cádiz (Andalucía) |
| Hospital Universitario Juan Ramón Jiménez | Huelva (Andalucía) |
| Hospital Universitario La Fe | Valencia (Comunidad Valenciana) |
| Hospital Universitario La Paz | Madrid (Comunidad de Madrid) |
| Hospital Universitario Lucus Augusti | Lugo (Galicia) |
| Hospital Universitario Marqués de Valdecilla | Cantabria (Cantabria) |
| Hospital Universitario Ramón y Cajal | Madrid (Comunidad de Madrid) |
| Hospital Universitario Regional de Málaga | Málaga (Andalucía) |
| Hospital Universitario Reina Sofía | Córdoba (Andalucía) |
| Hospital Universitario San Pedro | La Rioja (La Rioja) |
| Hospital Universitario Son Espases | Palma (Islas Baleares) |
| Hospital Universitario Torrecárdenas | Almería (Andalucía) |
| Hospital Universitario Vall d’Hebron | Barcelona (Cataluña) |
| Hospital Universitario Virgen de la Victoria | Málaga (Andalucía) |
| Hospital Universitario Virgen de las Nieves | Granada (Andalucía) |
| Hospital Universitario Virgen de Valme | Sevilla (Andalucía) |
| Hospital Universitario Virgen Macarena | Sevilla (Andalucía) |
