# Supplementary material for: Study protocol for a randomized clinical trial to assess 7 versus 14-days of treatment for Pseudomonas aeruginosa bloodstream infections (SHORTEN-2 trial)
Source: PLoS One. 2022 Dec 22;17(12):e0277333. doi: 10.1371/journal.pone.0277333 (PMC9778939; doi:10.1371/journal.pone.0277333)

S2 File. Definitions for main trial variables.

1. ***Treatment definitions***

***Active antibiotic treatment.*** Any antibiotic treatment with confirmed *in vitro* activity against the strain isolated in blood cultures according to EUCAST breakpoints will be considered active. Due to pragmatic reasons, and to enhance reproducibility of the trial results in real-life conditions, treatments prescribed before randomization will be considered active irrespective of dosing schemes, as far as no resistance is documented in the antibiogram. Specifically, aminoglycosides monotherapy will not be considered active except for the treatment of pyelonephritis. Breakpoints to consider antibiotic treatments as active are summarized in the following table:

| Agent | MIC |
| --- | --- |
| Ciprofloxacin | ≤0.5 mg/L |
| Levofloxacin | ≤1 mg/L |
| Piperacillin/tazobactam | ≤16 mg/L |
| Ceftazidime | ≤8 mg/L |
| Cefepime | ≤8 mg/L |
| Ceftazidime/avibactam | ≤8 mg/L |
| Ceftolozane/tazobactam | ≤4 mg/L |
| Imipenem | ≤4 mg/L |
| Meropenem | ≤8 mg/L |
| Meropenem-vaborbactam | ≤8 mg/L |
| Colistin | ≤2 mg/L |
| Amikacin | ≤16 mg/L |
| Tobramycin | ≤2 mg/L |
| Aztreonam | ≤16 mg/L |

***Optimized antibiotic treatment.*** An active antibiotic treatment administered following dosing and ways of administration recommended by EUCAST:

| Agent | Dosing |
| --- | --- |
| Ciprofloxacin | 750 mg/12h oral or 400 mg/8h i.v. |
| Levofloxacin | 500 mg/12h oral or i.v. |
| Piperacillin/tazobactam | 4 g/0.5 g/8h in 4h-extendend infusion.  4g/0.5 g/6h in 4h-extended infusión in case of strains resistant to ceftazidime or pneumonia. |
| Ceftazidime | 2 g/8h i.v. |
| Cefepime | 2 g/8h i.v. |
| Ceftazidime/avibactam | 2 g/8h i.v. in 2h-extended infusion |
| Ceftolozane/tazobactam | 1 g/0.5 g/8h. For pneumonia, the appropriate dose is 2 g/1 g/8h. |
| Imipenem | 1g/ 6h i.v. |
| Meropenem | MIC ≤2 mg/L: 1g/ 8h i.v.  MIC >2 mg/L and ≤8 mg/L: 2g/8h i.v in 3h infusion. |
| Meropenem/vaborbactam | 2g/2g/8h i.v. in 3h-extended infusion. |
| Colistin | 4.5 MU/12h i.v. |
| Amikacin | 25-30 mg/kg/24h i.v. |
| Tobramycin | 6-7 mg/kg/24h i.v. |
| Aztreonam | 2 g/6h i.v. |

The use of optimized treatments for all episodes of BSI-PA will be encouraged among all participating centers through didactic materials and meetings. All treatments will be optimized after randomization, if not previously performed.

1. ***Clinical variables*.**

***Charlson index***: The Charlson index is a standardized measure to predict risk of death within one year of hospitalization for patients depending on their specific comorbidities. *Charlson ME, Pompei P, Ales KL, MacKenzie CR. A new method of classifying prognostic comorbidity in longitudinal studies: development and validation. J Chronic Dis 1987;40(5):373-83.*

***Sepsis***: A life-threatening organ dysfunction caused by a dysregulated host response to infection. For clinical operationalization, organ dysfunction can be represented by an increase in the Sequential [Sepsis-related] Organ Failure Assessment (SOFA) score of 2 points or more. Patients who present with two or more of these signs during a known episode of bacteremia will also be considered septic: respiratory rate of 22/min or greater, altered mentation, or systolic blood pressure of 100 mm Hg or less (Singer M, et al. The Third International Consensus Definitions for Sepsis and Septic Shock (Sepsis-3). JAMA. 2016 Feb 23;315(8):801-10. doi: 10.1001/jama.2016.0287).

***Septic shock*:** Patients presenting with sepsis criteria and a vasopressor requirement to maintain a mean arterial pressure of 65 mm Hg or greater and serum lactate level greater than 2 mmol/L (>18 mg/dL) in the absence of hypovolemia ((Singer M, et al. The Third International Consensus Definitions for Sepsis and Septic Shock (Sepsis-3). JAMA. 2016 Feb 23;315(8):801-10. doi: 10.1001/jama.2016.0287).

***Source control.*** Interventions performed to remove the source of the BSI-PA, including: withdrawal of vascular catheters, urinary or biliary derivations, surgical debridement of infected tissues, surgical or percutaneous drainage of deep-seated abscesses, etc. In the case of urinary infections related to urinary catheters, the removal of the catheter is not mandatory to consider the source properly controlled.

***Multi-drug resistant P. aeruginosa***. Strains resistant to at least one agent in ≥3 classes of antibiotics will be considered multi-drug resistant, according to the latest definition of the European Center for Disease Control.

***Extensively-drug resistant P. aeruginosa***. Strains resistant to all classes of antibiotics except for one or two will be considered extensively drug resistant, according to the latest definition of the European Center for Disease Control.

1. ***Primary outcome measures***.

***Death***. Mortality due to any reason during the follow-up.

***Clinical cure***. The patient is alive and with resolution of the fever and the signs and symptoms of the infection that motivated the inclusion in the trial.

***Proven relapse***. Comprises two different scenarios (see flowchart in section ‘e’):

1. Recurrence of the signs and symptoms of the infection responsible for the inclusion of the patient in the trial after treatment cessation, in a patient whose symptoms had already resolved, with a new isolation of *P. aeruginosa* in blood cultures or in samples representative of the respective site of infection (see flowchart for assessing patients with recurrent symptoms in section ‘e’). Patients with a recurrent isolation of *P. aeruginosa* in microbiological samples without signs or symptoms of clinical infection of new onset are specifically excluded from this category (must be considered as colonizations).
2. Hematogenous seed in a new site of infection, secondary to the infection which motivated the inclusion in the trial, with microbiological samples from the new distant site of infection confirming the presence of *P. aeruginosa*.

***Probable relapse***. Comprises three different scenarios (see flowchart in section ‘e’):

1. Patients with recurrence of symptoms at the site of the infection that motivated the inclusion after the treatment cessation in a patient whose symptoms had already resolved, with no isolation of an alternative etiologic agent (either negative cultures or not collected). Patients with an alternative isolation different to *P. aeruginosa* are specifically excluded from this category (should be considered superinfections).
2. Patients with relapse of the BSI-PA without any focal symptom or signs suggesting a specific source, with no documentation of an alternative source for the new episode of bacteremia. Patients with a new episode of BSI-PA for whom an alternative source is documented, different from the infection that motivated the inclusion in the trial, are specifically excluded from this category (should be recorded as “new episode of BSI-PA”).
3. Clinical suspicion of hematogenous infection in a new site of infection secondary to the infection which motivated the inclusion in the trial, without available microbiological samples from the new distant site of infection, or with a negative culture of these samples. Patients with samples from the new distant site of infection which confirm an alternate etiology (different to *P. aeruginosa*) for this new infection are specifically excluded from this category (should be recorded as superinfections).

***Possible relapse***. Recurrence of the fever after treatment cessation in a patient whose signs and symptoms has already resolved, without focal symptom or signs suggesting an specific source, and with no other reason documented for the fever (see flowchart in section ‘e’).

1. ***Secondary outcome measures.***

***Treatment failure.*** Death for any cause, proven or probable relapse during the follow-up.

### *New episode of BSI-PA*. Isolation of *P. aeruginosa* in blood cultures after discontinuation of treatment in a patient with a previous negative blood culture.

### *Relapse of fever*. Reappearance of fever once apyrexia (temperature ≤37°C) has been reached for at least 72h.

### *Superinfection*. Infections by any etiology other than the one that motivated inclusion in the trial, including other Gram-negative bacilli, enterococci or staphylococci, C. difficile infection, and fungal infections by yeasts or filamentous fungi, during follow-up. Specifically, microbiological cultures obtained from non-sterile sites (urine, skin, respiratory tract) that do not correlate with a clinical infection syndrome will be interpreted as colonizations and not as superinfections.

***DOOR Category***. The DOOR group in which the patient is classified without considering the days of antibiotic treatment (see DOOR/RADAR section).

***Days free of antibiotic treatment***. Total number of days free of antibiotic treatment. For this purpose, the days on which at least one dose of antimicrobial treatment (antibiotic or antifungal) is received, whatever the indication (including antibiotic prophylaxis), will be subtracted from the total number of days observed in each patient.

***Days of hospitalization***. Days from the start of treatment to hospital discharge. All days of hospital stay observed during follow-up will be added together regardless of whether they are consecutive or not.

***Serious adverse events***. The number of serious adverse events will be collected and presented as a rate per 1,000 patient-days and the proportion of patients with at least one serious adverse event. For the definition of serious adverse events, refer to the latest version of the protocol.

***Recovery of baseline functional status***. The patient is able to perform the same activities that she or he was capable of before the episode of BSI-PA.

1. ***Flowchart for assessment of patients with recurrent symptoms or signs of infection.***


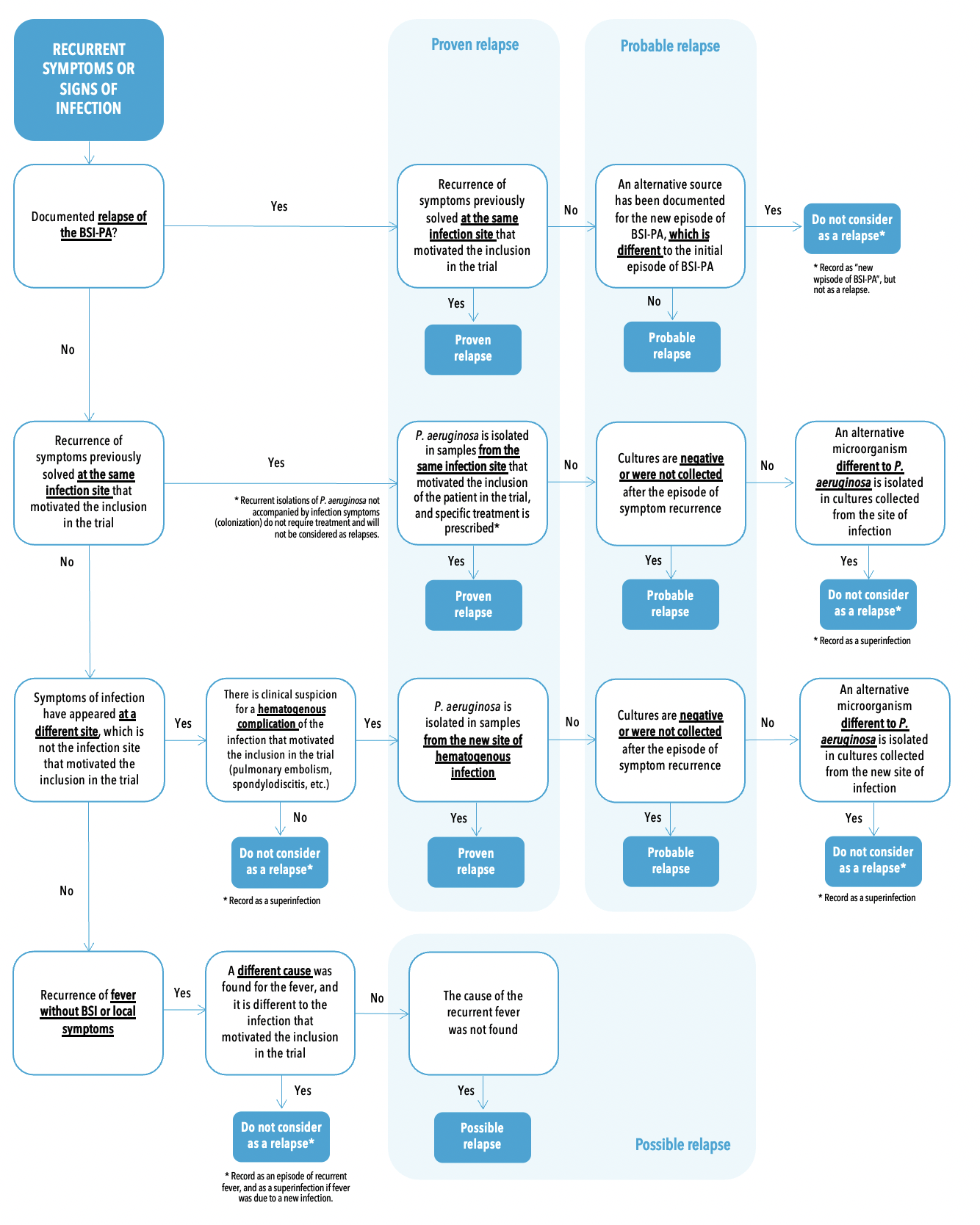

Supplement: S2 File — (DOCX) [file pone.0277333.s002.docx]
