# Supplementary material for: Study protocol for a randomized clinical trial to assess 7 versus 14-days of treatment for Pseudomonas aeruginosa bloodstream infections (SHORTEN-2 trial)
Source: PLoS One. 2022 Dec 22;17(12):e0277333. doi: 10.1371/journal.pone.0277333 (PMC9778939; doi:10.1371/journal.pone.0277333)
Supplement: S3 File — (DOCX) [file pone.0277333.s003.docx]

**STATISTICAL ANALYSIS PLAN**

**“Efficacy and safety of 7 versus 14 days of antibiotic treatment for Pseudomonas aeruginosa bacteraemia: a multicentre, randomized clinical trial (SHORTEN-2) with a DOOR/RADAR analysis”**

**CODE:** SHORTEN II

**EUDRACT NUMBER:** 2021-003847-10

**STATISTICAL ANALYSIS PLAN VERSION:** 1.0 January 24th, 2022.

**INDEX**

[1. Introduction 4](#_Toc93764141)

[1.1 Objectives 4](#_Toc93764142)

[2. Methods 4](#_Toc93764143)

[2.1 Design 4](#_Toc93764144)

[2.2 Randomization 4](#_Toc93764145)

[2.3 Sample size 5](#_Toc93764146)

[2.4 Data handling 6](#_Toc93764147)

[2.5 Timing of analysis 6](#_Toc93764148)

[2.6 Interim analysis 6](#_Toc93764149)

[3. Statistical Principles 7](#_Toc93764150)

[3.1 General considerations 7](#_Toc93764151)

[3.2. Analysis populations 7](#_Toc93764152)

[3.3 Protocol deviations 7](#_Toc93764153)

[4. Trial population 8](#_Toc93764154)

[4.1 Screening and recruitment 8](#_Toc93764155)

[4.2 Patient withdrawal 8](#_Toc93764156)

[4.3 Baseline patient characteristics 8](#_Toc93764157)

[5. Outcomes 8](#_Toc93764158)

[5.1 Primary outcome 8](#_Toc93764159)

[5.2 Secondary outcomes 8](#_Toc93764160)

[5.3 Outcome definitions 10](#_Toc93764161)

[*6.* Analysis 13](#_Toc93764162)

[6.1. Primary outcome 13](#_Toc93764163)

[6.2. Key secondary outcome 14](#_Toc93764164)

[6.3. Other secondary outcomes 14](#_Toc93764165)

[6.4 Subgroup and sensitivity analysis 14](#_Toc93764166)

[6.5 Missing data 15](#_Toc93764167)

[6.6. Safety analysis 15](#_Toc93764168)

[6.7 Statistical software 15](#_Toc93764169)

[7. References ­­16](#_Toc93764170)

# Introduction

## 1.1 Objectives

Primary objective: To determine whether a 7-day antibiotic treatment regimen is superior to a 14-day regimen in the treatment of *P. aeruginosa* bacteraemia, evaluating in an integrated manner both the effectiveness of the short regimen and its potential to reduce serious adverse events and antibiotic exposure.

Secondary objective of non-inferiority: To determine whether the short regimen is non-inferior to the long regimen in terms of treatment failure rate (defined as relapse and mortality).

Other secondary objectives:

To compare the effectiveness of both treatment arms in terms of mortality, DOOR scale, rate of proven, probable, or possible relapse, rate of recurrence of bacteraemia from any cause, or recovery of baseline functional class.

To compare the safety of both treatment arms assessed through the rate of adverse effects and superinfections, specifically those produced by multidrug-resistant bacteria and *C. difficile* in both treatment regimens.

To analyze the efficiency of the short regimens in terms of the number of treatment-free days and days of hospital stay avoided at the end of the follow-up period.

To determine risk factors related to treatment failure and risk of relapses.

# 2. Methods

## 2.1 Design

Pragmatic, phase IV, open-label, randomized, multicenter, pragmatic clinical trial to demonstrate the superiority of antibiotics with an authorized indication for 7 days vs. 14 days in the treatment of *P. aeruginosa* bacteraemia using DOOR (Desirability of Outcome Ranking)/RADAR (Response Adjusted for Duration of Antibiotic Risk) [1].

The study will be sufficiently powered to prove the superiority of the short treatment using the DOOR/RADAR variable. Only if the superiority of this variable is demonstrated will the non-inferiority analysis be performed for the composite variable of all-cause mortality and relapse.

All analyses performed will assess treatment efficacy at 30 days after stopping treatment and at 90 days from blood culture extraction that allowed entry into the trial (see the latest version of the protocol for a complete description).

## 2.2 Randomization

The trial's randomization will occur on the sixth day of antibiotic treatment (±24h) in a 1:1 ratio to the experimental or control arm. Randomization will be simple and stratified by center and pulmonary/extrapulmonary origin for bacteraemia.

## 2.3 Sample size

Definition of superiority. The superiority of the experimental treatment for the primary outcome variable of the study is established if the 7-day treatment is ≥60% better than the 14-day treatment, as assessed by the DOOR/RADAR scale.

To calculate the sample size, the Mann-Whitney U test was performed for an alternative hypothesis of a 60% probability that X > Y (with "X" being the DOOR/RADAR score of a randomly selected patient from the experimental group, and "Y" being the DOOR/RADAR score of a randomly selected patient from the control group), following the previously published methodology[2]. To confirm this superiority hypothesis, the estimated necessary sample size will be 262 patients, assuming an error α = 0.05, a power of 80%.

The hypothesis of non-inferiority will be evaluated for the treatment failure variable assessed at 30 days after discontinuation of trial treatment. An observational study published by Bae et al.[3], reports an all-cause mortality and realpses of 15.6% and 11.3% for patients receiving prolonged or short treatments. Considering this result and choosing a non-inferiority margin of 7.5%, a power of 80%, a one-sided α-error of 0.025, the estimated sample size would be 262.

The final number of the sample has been adjusted, expecting a non-adherence to the treatment of 5% and a loss to follow-up of 5% so that the estimated sample size needed to analyze the hypothesis of non-inferiority will be 306 patients. Thus, sufficient power is estimated for both variables.

### Justification

The chosen non-inferiority margin of 7.5% is more stringent than that used in other antibiotic duration shortening trials, in which 10% has been used. [4]. However, since the composite variable includes a highly relevant outcome (including death from any cause), we believe that its reduction in this study is justified.

Non-adherence may increase the risk of type 1 error in non-inferiority trials, a problem that is present in studies evaluating treatment duration [5]. In the study by von Dach et al.[6] 10.9% of the patients in the short arms received a longer treatment than indicated, and 7.9% of the patients in the standard arm received a shorter treatment. These failures to comply with the protocol were due in 90% of the cases to logistic and communication problems or the clinician's decision in the absence of complications that would indicate a change in the therapeutic regimen. If these causes are excluded, only 0.6% and 1.8% in the experimental and control groups received treatment different from expected. We believe that, by delaying randomization -which can be performed on the same day as the suspension of antibiotic treatment in the experimental arm- associated with close monitoring, we can avoid these causes of non-adherence. Thus, conservatively, we will calculate the sample for approximately double the number observed in this trial (1.5% non-adherence in the experimental arm and 3.5% in the control arm). In doing so, we will increase the sample by the factor obtained from 1/(1-*Pe-Pc*)^2^, where *Pe* is the proportion of non-adherents in the experimental group and *Pc* is the proportion of non-adherents in the control group [7].

Based on recent studies comparing standard vs. short treatment, we expect losses of less than 5%.[8]–[10]. However, to conservatively try to guarantee the power of the study, we will readjust the sample assuming 5% losses, as in other current protocols [11]. The sample will then be expanded by multiplying it by the factor obtained from 1/(1-*Pp*), where *Pp* is the expected loss proportion.

Given that the two variables are dependent (the key secondary is related to the primary), no significant increase in type 2 error is expected. [12].

## 2.4 Data handling

The information will be collected in the electronic case report form (eCRD - BD-SHORTEN II). Only data stored in this database will be analyzed. No data will be exported for analysis until the data have been validated by the study investigators and database managers.

Blinding of data analyses. As described in the study protocol, all data analyses will be performed in a blinded fashion. Therefore, patient data, including the trial arm, will be blinded.

## 2.5 Timing of analysis

An interim analysis will be performed when 40% of the projected patients have completed follow-up, which an independent committee will evaluate to assess safety and futility (see section 2.6). The final analysis will be performed once the database is closed.

## 2.6 Interim analysis

An interim analysis will be performed when 40% of the sample is recruited. This will be performed by an independent evaluation committee that will have full access to all study data (see section 2.6.1). The potential futility of the trial will be assessed through the conditional power (CP) for the safety variable. It will be done according to the trend observed in the interim analysis and that projected if the initial hypothesis is met. A CP of <15% is considered to make it unlikely to demonstrate the efficacy of the treatment[13], so discontinuation of the trial will be jointly considered if this margin is crossed. In addition, any differences in mortality between groups will be carefully evaluated. [14].

### *2.6.1*. Independent Evaluation Committee

To avoid, as far as possible, the bias that the open-label nature of the trial could generate, the evaluation of the results will be performed by a blinded independent committee (not taking part in the study as investigators) to treatment assignment. This committee will be formed by three expert investigators belonging to the CIBER of infectious diseases and will reach its conclusions by consensus (DBSM). The composition of these members will be communicated to the Ethics Committee before the start of the trial.

The main responsibilities of the DSMB are

1. Periodically review and evaluate the accumulated study data for participant safety, study conduct and progress, and, when appropriate, efficacy.
2. Make recommendations to the study coordination team on the trial's continuation, modification, or termination. For this purpose, a clear definition of the meeting schedule, stopping rules with statistical descriptions, and selected committee members will be approved prior to the start of the study.

# 3. Statistical Principles

## 3.1 General considerations

This is a randomized controlled clinical trial with standard treatment. A primary variable will be assessed, and only if this is met will a key secondary variable be assessed.

Continuous variables will be presented with the number of observations, median and interquartile range. Categorical variables will be presented as absolute numbers and percentages, with the denominator of these proportions being the total number of patients excluding those lost for that variable.

The chi-square test or Fisher's exact test will be used to compare discrete variables in the two study groups. Student's t-test will be used for continuous variables if they are normally distributed, and Mann-Whitney U for continuous variables that do not meet this requirement and for ordinal variables.

The results will be presented with bilateral 95% confidence intervals according to the NewCombe method, and bilateral p-values <0.05 will be considered significant, except for the key secondary variable, which, when evaluating non-inferiority, will be presented with the 97.5% one-sided confidence interval and in which a one-sided p < 0.025 will be considered significant.

The analyses performed will adhere to the standards indicated by the CONSORT guidelines.

## 3.2. Analysis populations

Analyses of primary and secondary outcomes will be performed on intention-to-treat (ITT) and per-protocol (PP) populations. These are defined as follows:

- Intention-to-treat population: all randomized patients constitute this population, whether or not patients continued to receive antibiotic therapy.
- Per-protocol population: This group consists of all randomized patients, received the assigned duration of antibiotic therapy (within ± 48 hours), had follow-up at day 90, and in whom no major protocol deviations were documented during the study period.

## 3.3 Protocol deviations

Major protocol deviations are:

- Randomization (by error) despite not meeting study entry criteria.
- Receipt of non-active antibiotic(s) after randomization for the infection motivating study inclusion.
- Difference in the duration of antibiotic therapy > 48 hours in fixed arms.
- Switch to another arm during the trial.

For a complete description of the inclusion and exclusion criteria, we refer to the latest version of the protocol.

# 4. Trial population

## 4.1 Screening and recruitment

The causes of screening failure will be summarized in tabular form (refer to the latest version of the protocol for inclusion and exclusion criteria). Similarly, withdrawals will be presented in tables by treatment group and completed visits.

An explanatory flow chart will be generated, according to CONSORT guidelines, describing the number of patients screened, randomized, lost to follow-up, analyzed by ITT and PP, as well as the number excluded from PP with their causes.

## 4.2 Patient withdrawal

A patient will be withdrawn from the trial due to (1) patient decision, (2) loss to follow-up, (3) safety criteria that make it inadvisable to continue in the trial, and (4) major protocol violation. The scheduled visits will continue with the ITT analysis in the last two cases. In the case of patients who wish to leave the study will be offered the possibility of continuing with the follow-up visits even if they abandon the treatment. If this option is rejected, the termination visit will be carried out at that time.

## 4.3 Baseline patient characteristics

To ascertain the comparability of the groups, the demographic characteristics, and main clinical variables according to the treatment arm and the population studied (ITT and PP) will be collected in tabular form. Baseline clinical data include, among others, disease severity at the time of infection (according to qSOFA score), the origin of bacteraemia, type of infection acquisition (community-acquired, community-acquired, or nosocomial), baseline microbiological data (e.g., presence of a multidrug-resistant bacterial infection of multidrug-resistant bacteria at inclusion; see definition below), immunosuppression, and renal function.

# 5. Outcomes

## 5.1 Primary outcome

The probability that any patient in the experimental arm achieves better outcomes than a patient in the control group assessed through their DOOR/RADAR analysis score assessed at day +30 from discontinuation of trial treatment.

## 5.2 Secondary outcomes

The secondary non-inferiority endpoint is therapeutic failure assessed at day +30 from discontinuation of trial treatment. As a secondary analysis, an additional analysis of the primary and secondary non-inferiority variable will be performed at day +90 from the date of the extraction of the first positive blood culture.

Other secondary variables will be:

| **Variable** | **Periodo de seguimiento** |
| --- | --- |
| Mortality from any cause | Day +30 from discontinuation of trial treatment.  Day +90 from the date of extraction of the first positive blood culture. |
| Clinical cure | Day +30 from discontinuation of trial treatment.  Day +90 from the date of extraction of the first positive blood culture. |
| Relapses | Day +30 from discontinuation of trial treatment.  Day +90 from the date of extraction of the first positive blood culture. |
| New episode of BSI-PA. | Day +30 from discontinuation of trial treatment.  Day +90 from the date of extraction of the first positive blood culture. |
| Recurrence of fever | Day +30 from discontinuation of trial treatment.  Day +90 from the date of extraction of the first positive blood culture. |
| Superinfections | Day +30 from discontinuation of trial treatment.  Day +90 from the date of extraction of the first positive blood culture. |
| DOOR Category | Day +30 from discontinuation of trial treatment.  Day +90 from the date of extraction of the first positive blood culture. |
| Serious adverse events | Day +30 from discontinuation of trial treatment.  Day +90 from the date of extraction of the first positive blood culture. |
| Antibiotic-free days | Day +30 from discontinuation of trial treatment.  Day +90 from the date of extraction of the first positive blood culture. |
| Days of hospitalization | Day +30 from discontinuation of trial treatment.  Day +90 from the date of extraction of the first positive blood culture. |
| Recovery of baseline functional capacity | Day +30 from discontinuation of trial treatment.  Day +90 from the date of extraction of the first positive blood culture. |

## 5.3 Outcome definitions

**DOOR/RADAR**

The primary variable will be the probability that any patient in the experimental arm will achieve better outcomes than a patient in the control group, as assessed by their DOOR / RADAR analysis score. This analysis categorizes patients into two classes:

(a) A first ordinal classification of clinical outcomes (DOOR), defined by the following mutually exclusive categories, assessed at the end of follow-up:

1. uneventful cure.

2. Cure with proven or probable relapse.

3. Cure with a serious adverse event.

4. No clinical cure.

5. Death.

b) A second classification in which patients in the same clinical outcome category are classified according to the number of days of antibiotic treatment (RADAR), defined as the number of days of antimicrobial treatment from the time of collection of the first positive blood culture and up to 30 days after discontinuation of trial treatment. Measurement of the number of antimicrobial treatment days will include all antibiotics and antifungals indicated during that period as empiric or targeted treatment of superinfections occurring during that follow-up period. Antimicrobials indicated as prophylaxis will not be included.

Therefore, the rank of outcome is established as the priority classification criterion, subordinating the assessment of the duration of treatment to patients with equal clinical outcomes. Thus, patients with a lower DOOR / RADAR score will be those with better results in terms of clinical effectiveness and reduced exposure to antibiotic treatment.

### Therapeutic failure

Death from any cause or proven or probable relapse during follow-up (day +30 of treatment interruption and +90 of blood culture extraction).

**Special considerations:** an expert committee to evaluate patients with treatment failure, specifically probable relapse, is the non-objective outcome variable. Due to the above criteria, they will undergo final evaluation by a panel of study investigators. This committee will be blinded to be unaware of the assignment of the patient being evaluated to the experimental or control group. The three investigators of the committee will be asked to confirm or not the relapse of bacteraemia, providing, in this case, an alternative explanation. In case of discrepancy, the majority vote will take the final decision.

### Death

Death of the patient from any cause during follow-up.

### Clinical cure

Patient alive, with resolution of fever and signs and symptoms of infection responsible for inclusion in the trial and negative control blood cultures during follow-up.

### Relapses

Includes the presence of a proven, probable, or possible relapse during patient follow-up. The following definitions of these events can also be found in the flow chart in supplementary file 2.e.:

Proven relapse

Proven relapse will be considered:

1. New episode of *P. aeruginosa* bacteraemia associated with symptoms at the same site as the previous infection that motivated inclusion in the trial.
2. Reappearance of previously resolved symptoms and signs of the infection that led to inclusion in the trial, with new isolation of *P. aeruginosa* in representative samples from the site of infection.
3. Occurrence of local symptoms of infection at a different site, suspected to be the result of a hematogenous complication (embolism, spondilodyscytis...) of the infection that led to inclusion in the trial, in which *P. aeruginosa* is isolated at the new site of infection.

Probable relapse

Probable relapse will be considered:

1. New episode of *P. aeruginosa* bacteraemia without presenting symptoms of infection at the same site as the previous infection that motivated inclusion in the trial and a different focus is not identified.
2. b) Reappearance of previously resolved symptoms and signs of the infection that led to inclusion in the trial, without new isolation of *P. aeruginosa* in representative samples from the site of infection, either because of negative cultures or because microbiological studies have not been performed.
3. Occurrence of local symptoms of infection at a different site, suspected to be the result of a hematogenous complication (embolism, spondilodyscytis...) of the infection that led to inclusion in the trial, without new isolation of *P. aeruginosa* in samples representative of the site of infection, either because of negative cultures or because microbiological studies have not been performed.

Possible relapse

A relapse of fever after discontinuation of treatment in a patient whose symptoms had already resolved, without new episodes of *P. aeruginosa* bacteraemia or symptoms specific to the site infection responsible for inclusion in the trial, and without any other defined alternative cause for the fever will be considered a possible relapse.

Other situations:

The following cases will not be considered relapses:

1. New episode of *P. aeruginosa* bacteraemia without presenting symptoms of infection at the same site as the previous infection responsible for inclusion in the trial and where a different focus is identified.
2. Reappearance of previously resolved symptoms and signs of the infection that led to inclusion in the trial, in which a microorganism other than *P. aeruginosa* is isolated and considered responsible for the symptoms.
3. Occurrence of local symptoms of infection at a different site, in which:
   1. It is not suspected to be a hematogenous complication of the infection that prompted the trial's inclusion.
   2. It is suspected to result from a hematogenous complication (embolisms, spondilodyscytis...) of the infection that motivated inclusion in the trial, but a microorganism other than *P. aeruginosa* is isolated and it is considered responsible for the symptoms.
4. Recurrence of fever after discontinuation of treatment in a patient whose symptoms had already resolved, in which a cause of fever other than the infection that led to inclusion in the trial is identified.

### New episode of BSI-PA.

Isolation of *P. aeruginosa* in blood cultures after discontinuation of treatment in a patient with a previous negative blood culture.

### Recurrence of fever

Reappearance of fever once apyrexia (temperature ≤37°C) has been reached for at least 72h.

### Superinfection

Infections by any etiology other than the one that motivated inclusion in the trial, including other Gram-negative bacilli, enterococci or staphylococci, C. difficile infection, and fungal infections by yeasts or filamentous fungi, during follow-up.

Specifically, microbiological cultures obtained from non-sterile sites (urine, skin, respiratory tract) that do not correlate with a clinical infection syndrome will be interpreted as colonizations and not as superinfections.

### DOOR Category

The DOOR group in which the patient is classified without considering the days of antibiotic treatment (see DOOR/RADAR section).

### Days free of antibiotic treatment

Total number of days free of antibiotic treatment. For this purpose, the days on which at least one dose of antimicrobial treatment (antibiotic or antifungal) is received, whatever the indication (including antibiotic prophylaxis), will be subtracted from the total number of days observed in each patient.

### Days of hospitalization

Days from the start of treatment to hospital discharge. All days of hospital stay observed during follow-up will be added together regardless of whether they are consecutive or not.

### Serious adverse events

The number of serious adverse events will be collected and presented as a rate per 1,000 patient-days and the proportion of patients with at least one serious adverse event.

For the definition of serious adverse events, refer to the latest version of the protocol.

### Recovery of functional capacity

The patient self-reported the recovery of the baseline situation prior to the episode of bacteraemia.

# *6.* Analysis

## 6.1. Primary outcome

As stated in the protocol, a superiority hypothesis is postulated, expecting that seven days of antibiotic therapy will be superior to 14 days of antibiotic therapy in patients meeting the inclusion criteria for this trial. Analyses will be performed in the PP and ITT populations.

The probability that a patient in the randomly selected experimental group has a better DOOR/RADAR outcome than a patient in the control group is obtained from the between-treatment pairwise comparisons in which the experimental group have a better outcome than the control in pairwise comparisons (ties will be assigned 0.5) divided by the total number of possible pairwise comparisons[1], [15]. The superiority hypothesis will be considered proven if the lower limit of the 95% confidence interval calculated by Newcombe's method is above 60%.

## 6.2. Key secondary outcome

For the non-inferiority analysis, the difference in the proportion of patients with therapeutic failure in each arm and its one-tailed 97.5% confidence interval will be calculated by calculating the Newcombe-Wilson score.

The hypothesis of non-inferiority will be considered proven if the upper limit of this confidence interval is below 7.5%. Since there is an established hierarchical order in which non-inferiority cannot be declared without first demonstrating the superiority of the primary variable, no readjustment of alpha has been performed[16].

## 6.3. Other secondary outcomes

The rest of the secondary variables are considered exploratory and hypothesis-generating; therefore, the statistical values and confidence intervals are descriptive, and no adjustment for multiplicity has been performed.[16]. These results will be presented in a forest plot with the absolute difference between the groups and their 95% confidence intervals.

## 6.4 Subgroup and sensitivity analysis

The following predefined subgroup analyses are established in which the primary variable will be assessed:

1. patients with bacteraemia of pulmonary focus versus patients with bacteraemia of extrapulmonary foci.
2. Patients who have started optimized treatment in the first 48h from the date of extraction of the first blood cultures versus those who start it after the first 48h.
3. Patients receiving immunosuppressive treatment.

In any case, the study does not have the necessary power to detect differences between the subgroups, so these results should be considered exploratory and hypothesis-generating.

As a sensitivity analysis, the study variables will be evaluated stratified by center and origin of the bacteraemia (pulmonary/non-pulmonary). The same procedure will be followed if any prognostic factor is not adequately balanced between the groups.

## 6.5 Missing data

Every effort will be made to collect data per protocol. Multiple imputation will be performed if the missing data is more than 5% of the subjects and the necessary conditions for such a procedure are met [17]. Using covariates and outcome variables, 20 datasets will be imputed [18].

## 6.6. Safety analysis

### 6.6.1 Adverse events

The safety analysis will be performed on the ITT population. The number of patients presenting at least one adverse event will be tabulated according to the treatment arm and grouped according to the Medical Dictionary of Regulatory Activities (MedDRA) preferred term. All SAEs and those AEs (according to the MedDRA term) that exceed 5% in any arms will be presented in tables. Additional tables will be presented according to the degree of a causal relationship with the treatment (possible, probable, or definite). The proportion of patients in each arm will be calculated by dividing the number of patients with at least one AE by the number of ITT patients in that arm.

Also, these same calculations will be presented on the ITT population, but in the form of rates (per 1,000 patient-days) of SAEs and AEs under the same assumptions. In this case, the quotient will be the number of adverse events and the denominator the number of patient-days. The number of patient-days will be obtained from the total number of days observed in each arm, from day 0 to censoring each patient (due to study termination, death, or loss to follow-up).

### 6.6.2 Treatment adherence

The number of patients in each group who presented a deviation from the assigned treatment duration will be tabulated. The cases will be presented according to whether such duration (shortened, prolonged, or unknown) and according to its cause: screening failure, logistic error/lack of communication between the research team, physician's decision in the absence of a suspected complication, failure or adverse effect, patient's decision, withdrawal of consent or loss to follow-up. Baseline characteristics will also be compared between adherent and non-adherent patients according to their causes.

## 6.7 Statistical software

All analyses will be performed with the statistical programs R version 4.1.2 and SPSS version 22.0.

# 7. References

[1] S. R. Evans *et al.*, “Desirability of Outcome Ranking (DOOR) and Response Adjusted for Duration of Antibiotic Risk (RADAR).,” *Clin. Infect. Dis. an Off. Publ. Infect. Dis. Soc. Am.*, vol. 61, no. 5, pp. 800–806, Sep. 2015, doi: 10.1093/cid/civ495.

[2] G. E. Noether, “Sample Size Determination for Some Common Nonparametric Tests,” *J. Am. Stat. Assoc.*, vol. 82, no. 398, pp. 645–647, Jun. 1987, doi: 10.1080/01621459.1987.10478478.

[3] M. Bae *et al.*, “Short versus prolonged courses of antimicrobial therapy for patients with uncomplicated Pseudomonas aeruginosa bloodstream infection: a retrospective study.,” *J. Antimicrob. Chemother.*, Sep. 2021, doi: 10.1093/jac/dkab358.

[4] J. H. Rex *et al.*, “Progress in the Fight Against Multidrug-Resistant Bacteria 2005-2016: Modern Noninferiority Trial Designs Enable Antibiotic Development in Advance of Epidemic Bacterial Resistance.,” *Clin. Infect. Dis. an Off. Publ. Infect. Dis. Soc. Am.*, vol. 65, no. 1, pp. 141–146, Jul. 2017, doi: 10.1093/cid/cix246.

[5] Y. Mo, C. Lim, J. A. Watson, N. J. White, and B. S. Cooper, “Non-adherence in non-inferiority trials: pitfalls and recommendations.,” *BMJ*, vol. 370, p. m2215, Jul. 2020, doi: 10.1136/bmj.m2215.

[6] E. von Dach *et al.*, “Effect of C-Reactive Protein-Guided Antibiotic Treatment Duration, 7-Day Treatment, or 14-Day Treatment on 30-Day Clinical Failure Rate in Patients With Uncomplicated Gram-Negative Bacteraemia: A Randomized Clinical Trial.,” *JAMA*, vol. 323, no. 21, pp. 2160–2169, Jun. 2020, doi: 10.1001/jama.2020.6348.

[7] J. Wittes, “Sample size calculations for randomized controlled trials.,” *Epidemiol. Rev.*, vol. 24, no. 1, pp. 39–53, 2002, doi: 10.1093/epirev/24.1.39.

[8] R. G. Sawyer *et al.*, “Trial of short-course antimicrobial therapy for intraabdominal infection.,” *N. Engl. J. Med.*, vol. 372, no. 21, pp. 1996–2005, May 2015, doi: 10.1056/NEJMoa1411162.

[9] D. M. Drekonja, B. Trautner, C. Amundson, M. Kuskowski, and J. R. Johnson, “Effect of 7 vs 14 Days of Antibiotic Therapy on Resolution of Symptoms Among Afebrile Men With Urinary Tract Infection: A Randomized Clinical Trial.,” *JAMA*, vol. 326, no. 4, pp. 324–331, Jul. 2021, doi: 10.1001/jama.2021.9899.

[10] A. Dinh *et al.*, “Discontinuing β-lactam treatment after 3 days for patients with community-acquired pneumonia in non-critical care wards (PTC): a double-blind, randomized, placebo-controlled, non-inferiority trial.," *Lancet (London, England)*, vol. 397, no. 10280, pp. 1195–1203, Mar. 2021, doi: 10.1016/S0140-6736(21)00313-5.

[11] N. Daneman *et al.*, "Bacteraemia Antibiotic Length Actually Needed for Clinical Effectiveness (BALANCE) randomized clinical trial: study protocol.," *BMJ Open*, vol. 10, no. 5, p. e038300, May 2020, doi: 10.1136/bmjopen-2020-038300.

[12] Food and Drug Administration, “Multiple endpoints in clinical trials: guidance for industry,” *FDA Guid.*, no. January, 2017.

[13] E. Lesaffre *et al.*, “Statistical controversies in clinical research: futility analyses in oncology-lessons on potential pitfalls from a randomized controlled trial.,” *Ann. Oncol. Off. J. Eur. Soc. Med. Oncol.*, vol. 28, no. 7, pp. 1419–1426, Jul. 2017, doi: 10.1093/annonc/mdx042.

[14] S. J. Pocock, “Current controversies in data monitoring for clinical trials.,” *Clin. Trials*, vol. 3, no. 6, pp. 513–521, 2006, doi: 10.1177/1740774506073467.

[15] S. R. Evans and D. Follmann, “Using Outcomes to Analyze Patients Rather than Patients to Analyze Outcomes: A Step Toward Pragmatism in Benefit:Risk Evaluation,” *Stat. Biopharm. Res.*, vol. 8, no. 4, pp. 386–393, 2016, doi: 10.1080/19466315.2016.1207561.

[16] "European Medicine Agency (EMA). Guideline on Multiplicity Issues in Clinical Trials (Draft)," 2017. https://www.ema.europa.eu/en/documents/scientific-guideline/draft-guideline-multiplicity-issues-clinical-trials_en.pdf (accessed December 12th, 2021).

[17] J. C. Jakobsen, C. Gluud, J. Wetterslev, and P. Winkel, "When and how should multiple imputation be used for handling missing data in randomized clinical trials - a practical guide with flowcharts.," *BMC Med. Res. Methodol.*, vol. 17, no. 1, p. 162, Dec. 2017, doi: 10.1186/s12874-017-0442-1.

[18] J. W. Graham, A. E. Olchowski, and T. D. Gilreath, “How many imputations are really needed? Some practical clarifications of multiple imputation theory,” *Prev. Sci.*, vol. 8, no. 3, pp. 206–213, 2007, doi: 10.1007/s11121-007-0070-9.
