# Supplementary material for: Study protocol for a randomized clinical trial to assess 7 versus 14-days of treatment for Pseudomonas aeruginosa bloodstream infections (SHORTEN-2 trial)
Source: PLoS One. 2022 Dec 22;17(12):e0277333. doi: 10.1371/journal.pone.0277333 (PMC9778939; doi:10.1371/journal.pone.0277333)
Supplement: S4 File — (DOCX) [file pone.0277333.s004.docx]

S4 File. Results of the survey among participating centers regarding diagnostic and clinical routines for the management of BSI-PA.

|  | Number of  complying centers  n/N (%) (N=30) |  |
| --- | --- | --- |
|  | |  |
| General features of the center and the research team | |  |
| Hospital with > 500 beds | 24/30 (80) |  |
| Number of PA-BSI reported per year (n) |  |  |
| Less than 20 episodes | 2/30 (7) |  |
| Between 20 and 50 | 17/30 (57) |  |
| Between 50 and 100 | 10/30 (33) |  |
| More than 100 | 1/30 (3) |  |
| Local prevalence of MDR P. aeruginosa |  |  |
| Below 10% | 13/30 (43) |  |
| Between 10-20% | 12/30 (40) |  |
| Between 20-30% | 2/30 (7) |  |
| Not routinely monitored | 3/30 (10) |  |
| How many patients expected to be recruited per year? | |  |
| Less than 5 | 3/30 (10) |  |
| From 5 to 10 | 19/30 (63) |  |
| More than 10 | 8/30 (27) |  |
| Some of the members of the research team are involved in regular clinical attendance of patients of bacteremia | 29/30 (97) |  |
| How confident is the research team in the trial hypothesis? |  |  |
| Confident or very confident | 24/30 (80) |  |
| Moderately confident | 6/30 (20) |  |
|  |  |  |
| Microbiological procedures | |  |
| Microbiology laboratory available 7 days a week | 30/30 (100) |  |
| Microbiology laboratory available 24 hours a day | 25/30 (83) |  |
| Blood cultures results informed 24 hours a day | 22/30 (73) |  |
| Full susceptibility reports available for blood cultures in up to 24 hours after positivity. | 27/30 (90) |  |
| Rapid diagnostic tests are usually performed for resistance detection in PA-BSI. | 15/30 (50) |  |
| PCR-based techniques | 6/30 (20) |  |
| Immunochromatography-based tecniques | 9/30 (30) |  |
| Early antibiogram report (6 hours) | 8/30 (27) |  |
| Clinical procedures | |  |
| Local guidelines available with specific recommendations on treatment of PA-BSI, based on local epidemiology | 10/30 (33) |  |
| Infectious diseases expert available for clinical counseling on management of PA-BSI | 29/30 (97) |  |
| Blood cultures informs are linked to an antimicrobial stewardship program for optimizing diagnostic and therapeutic approach | 28/30 (93) |  |
| How frequently are increased dosing employed for PA-BSI, as recommended by EUCAST? |  |  |
| All prescriptions | 4/30 (13) |  |
| Most prescriptions | 14/30 (47) |  |
| Only selected cases | 12/30 (40) |  |
| How frequently are extended or continuous infusion employed for treating PA-BSI? |  |  |
| All prescriptions | 4/30 (13) |  |
| Most prescriptions | 10/30 (33) |  |
| Only selected cases or not employed | 16/30 (54) |  |
| How frequently are combined schemes employed as empirical therapy of PA-BSI pending definitive antibiogram report? |  |  |
| Infrequent or very infrequent | 15/30 (50) |  |
| Rather frequent | 8/30 (27) |  |
| Frequent or very frequent | 7/30 (23) |  |
| How frequently are combined schemes employed as targeted therapy of PA-BSI by fully-susceptible strains? |  |  |
| Infrequent or very infrequent | 24/30 (80) |  |
| Rather frequent | 4/30 (13) |  |
| Frequent or very frequent | 2/30 (7) |  |
| What is the most usual duration for treating uncomplicated cases of PA-BSI? |  |  |
| 14 days or more | 14/30 (47) |  |
| 10 days | 13/30 (43) |  |
| 7 days or less | 3/30 (10) |  |
| Available OPAT program which allows the administration of main antipseudomonal betalactams to outpatients | 15/30 (50) |  |
| Available therapeutic drug monitoring of antipseudomonal agents in clinical practice |  |  |
| Aminoglycosides | 28/30 (23) |  |
| Betalactams | 5/30 (17) |  |
| Colistin | 2/30 (7) |  |
