# Supplementary material for: Study protocol for a randomized clinical trial to assess 7 versus 14-days of treatment for Pseudomonas aeruginosa bloodstream infections (SHORTEN-2 trial)
Source: PLoS One. 2022 Dec 22;17(12):e0277333. doi: 10.1371/journal.pone.0277333 (PMC9778939; doi:10.1371/journal.pone.0277333)
Supplement: S5 File — (PDF) [file pone.0277333.s005.pdf]

CLINICAL TRIAL PROTOCOL

"Efficacy and safety of 7 versus 14 days of antibiotic treatment for *Pseudomonas aeruginosa* bacteraemia: a multicentre, randomized clinical trial (SHORTEN-2) with a DOOR/RADAR analysis".

CODE: SHORTEN II

EUDRACT NO: 2021-003847-10

VERSION: 2.0 of 17 January 2022

Version history of the study protocol.

Date	Version	Changes	Authors
15 October 2021	1.0		
17 January 2022	2.0		

SUMMARY PROTOCOL	
TYPE OF APPLICATION	Clinical trial to evaluate the efficacy and safety of 7 vs. 14 days of different licensed antibiotics in the treatment of *Pseudomonas aeruginosa* bacteraemia.
IDENTIFICATION OF THE PROMOTER	Andalusian Public Foundation for the Management of Health Research in Seville (FISEVI).<br>Virgen del Rocío University Hospital.<br>Laboratory building, 6th floor.<br>Avda. Manuel Siurot S/N<br>41013/Seville<br>Tel. 955012820
TITLE OF THE ESSAY	"Efficacy and safety of 7 versus 14 days of antibiotic treatment for *Pseudomonas aeruginosa* bacteraemia: a multicentre, randomized clinical trial (SHORTEN-2) with a DOOR/RADAR analysis".
PROTOCOL CODE	SHORTEN II
COORDINATING RESEARCHER	Dr. José Miguel Cisneros Herreros<br>Infectious Diseases, Microbiology and Preventive Medicine CMU<br>Hospital Universitario Virgen del Rocío/ Instituto de Biomedicina de Sevilla (IBiS)<br>Avda. Manuel Siurot s/n<br>41013 Seville<br><br>Clinical Coordinator<br>Dr. José Molina Gil-Bermejo<br>Infectious Diseases, Microbiology and Preventive Medicine CMU<br>Virgen del Rocío University Hospital/ IBiS<br>Avda. Manuel Siurot s/n<br>41013 Seville<br><br>Microbiological Coordinator<br>Dr. José Antonio Lepe Jiménez

	Infectious Diseases, Microbiology and Preventive Medicine CMU<br>Virgen del Rocío University Hospital/ IBiS<br>Avda. Manuel Siurot s/n<br>41013 Seville<br><br>Clinical Pharmacist Coordinator<br>Dr. Ana Belén Guisado Gil<br>UGC of Hospital Pharmacy<br>Virgen del Rocío University Hospital/ IBiS<br>Avda. Manuel Siurot s/n<br>41013 Seville<br><br>Sample and Experimental Research Logistics Coordinator<br>Dr. María Eugenia Pachón Ibáñez<br>Infectious Diseases, Microbiology and Preventive Medicine CMU<br>Virgen del Rocío University Hospital/ IBiS<br>Avda. Manuel Siurot s/n<br>41013 Seville<br><br>Medical and security coordinator/monitor<br>Dr. Clara M Rosso Fernández<br>UICEC-HUVR, CDCA<br>Virgen del Rocío University Hospital/ IBiS<br>Avda. Manuel Siurot s/n<br>41013 Seville
SITES WHERE THE TRIAL IS PLANNED TO TAKE PLACE	Multicentre study involving Spanish hospitals 30 Spanish hospitals. Annex II
CLINICAL RESEARCH ETHICS COMMITTEE(S)	Reference Committee: CEIm provincial de Sevilla.
RESPONSIBLE FOR MONITORING	Clinical Research and Clinical Trials Unit, Hospital Universitario Virgen del Rocío-SCReN (*Spanish Research Network*).

EXPERIMENTAL TREATMENT AND CONTROL	The patients in the study will receive the antibiotic treatment chosen at the discretion of the physician responsible for the patient for the treatment of *P. aeruginosa* bacteraemia*, in accordance with the* protocols established in the hospital to which they belong.<br><br>The study intervention consists of monitoring the duration of active antibiotic treatment according to the groups described below:<br><br>● **Experimental group (short arm)**: shall receive seven days of active antibiotic treatment since the last positive blood culture for *P. aeruginosa* provided that the patient has been free of symptoms and signs of infection for the previous three days infection.<br><br>● **Control group (long arm)**: will receive 14 days of active antibiotic treatment from the last positive blood culture for *P. aeruginosa provided* that the patient has been free of symptoms and signs of infection for the previous three days.<br><br>After discontinuation of treatment, treatment may be resumed if the physician in charge of the patient deems it necessary due to an unfavourable outcome.
CLINICAL TRIAL PHASE	Phase IV
OBJECTIVES	Primary objective: To determine whether a 7-day antibiotic treatment regimen is superior to a 14-day regimen in the treatment of *P. aeruginosa* bacteraemia, assessing in an integrated manner both the effectiveness of the short regimen and its potential to reduce serious adverse events and antibiotic exposure.<br><br>Secondary objectives:<br><br>1. Determine whether the short regimen is non-inferior to the long regimen in terms of recurrence of infection and mortality, and whether discontinuation can be safely applied at the

	individual level using a simple clinical decision rule.<br><br>2. Describe adverse effects and superinfections, specifically those caused by multi-resistant bacteria, fungi and *Clostridioides difficile* in both treatment regimens.<br><br>3. To analyze the efficiency of short regimens by looking at the number of treatment days and days of hospital stay avoided at the end of the follow-up period.<br><br>4. Check if recurrences are from the same strain by comparing *P. aeruginosa* strains by genetic sequencing.<br><br>5. To compare the ecological impact of short and prolonged treatment regimens by analyzing the diversity of gut microbiota.
DESIGN	Pragmatic, phase IV, open-label, randomized, multicentre, clinical trial to demonstrate the superiority of antibiotics with licensed indication for 7 days vs. 14 days in the treatment of *P. aeruginosa* bacteraemia.
STUDY DISEASE OR DISORDER	Monomicrobial bacteraemia caused by *P. aeruginosa.*
PRIMARY ENDPOINT	The primary endpoint will be the probability that any patient in the experimental arm achieves better outcomes than a patient in the control group as assessed by their DOOR/RADAR score.
STUDY POPULATION AND TOTAL NUMBER OF PATIENTS	The study population will consist of adult patients (18 years and older) with monomicrobial bacteraemia caused by *P. aeruginosa*.<br>The required sample size is 306 patients (153 per arm).

FOLLOW-UP	Ninety days from the date of withdrawal of the first positive blood culture.
DURATION OF TREATMENT	The initial duration of active antibiotic treatment will be 7 or 14 days, depending on the assigned arm. In patients assigned to the short (experimental) arm, active treatment will be discontinued on day 7 if the patient is apyretic and has no symptoms of infection in the previous 72 hours. In patients assigned to the long (control) arm, active treatment will be stopped on day 14 if the patient is apyretic and has no symptoms of infection in the previous 72 hours. If this clinical rule is not met, treatment should be maintained, and the patient will be reassessed every 48-72 hours. Once the above clinical criteria are met, treatment should be discontinued.
TIMETABLE AND EXPECTED COMPLETION DATE	The total duration of the trial is expected to be four years from the start of the recruitment period (2022 to 2025). A period of 3 months is included for the submission of documentation to the Spanish Agency for Medicines and Health Products (AEMPS) and the Ethics Committees, as well as initial training at the participating centres. The patient recruitment period will be 36 months and another 9 months are estimated for the analysis and subsequent dissemination of results.

1.- TABLE OF CONTENTS

2.- GENERAL INFORMATION 12
2.1 Identification of the clinical trial 12
2.2 Type of clinical trial 12
2.3 Data relating to the promoter 12
2.3.1 Person authorized by the promoter 12
2.3.2 Responsible for Monitoring 12
2.4 Description of the products under study 13
2.5 Data from the study investigators 14
2.6 Laboratories, Medical Department or Related Institutions 14
3.- JUSTIFICATION 15
4HYPOTHESES AND OBJECTIVES OF THE TRIAL 19
4.1 Hypotheses 19
4.2. Objectives 19
5. TRIAL DESIGN 20
5.1 Study variables 20
5.1.1 Main outcome variable 20
5.1.2 Secondary variables. 21
5.2 Design 22
5.3 Randomization procedure 23
5.4 Blinding 24
5.5 Study treatments 25
5.6 Allowed medicines and treatments 26
5.7 Follow-up of patients 26
5.8 Study termination or discontinuation criteria 27
5.9 Procurement, packaging and labelling of medication 27
5.10 Storage and dispensing of medication 27
5.11 End of trial 28

6.- SELECTION CRITERIA 28
6.1 Inclusion criteria 28
6.2 Exclusion criteria 28
6.3 Withdrawal criteria 29
6.3.1 By decision of the patient 29
6.3.2 Loss of tracking 29
6.3.3 By security criteria 30
6.3.4 For non-compliance or violation of the rules contained in the protocol 30
6.4 Follow-up of prematurely withdrawn patients 30
6.5 Definitions 30
New episode of bacteraemia: new isolation of P. aeruginosa in blood cultures from any cause after discontinuation of treatment in a patient who had already demonstrated a negative blood culture after the initial episode that led to inclusion in the trial. 33
Recurrence of fever: return of fever after reaching apyrexia (temperature ≤37°C) for at least 72h. 33
Days free of antibiotic treatment: total number of days free of antibiotic treatment. For this purpose, the days on which at least one dose of antimicrobial treatment (antibiotic or antifungal) is received for any indication (including antibiotic prophylaxis) shall be subtracted from the total number of days observed for each patient. 33
Days of hospitalization: days from the start of treatment to hospital discharge. All days of hospital stay observed during follow-up will be added together regardless of whether they are consecutive or not. 33
Serious adverse events: the number of serious adverse events shall be collected and presented as a rate per 1,000 patient-days and as a proportion of patients with at least one serious adverse event. 33
7.- TREATMENT OF SUBJECTS 34
7.1 Concomitant medication 34
7.2 Rescue medication 34
7.3 Schedule of visits and evaluations 34
7.4 Procedures per visit 35
7.5 Other Study Procedures (Sub-Studies) 39
7.5.1 Microbiological studies 39

7.5.2 Studies of bacterial diversity in the gut microbiota. 39

8.- ASSESSMENT OF EFFECTIVENESS 39
8.1 Efficiency variables 39

8.2 Laboratory tests 40

9.- SAFETY ASSESSMENT 40
9.1 Security assessments 40

9.1.1 Physical examination, vitals 40

9.1.2 Laboratory and microbiological tests 41

9.2 Adverse events of interest for follow-up 41

9.3 Definitions 41

9.4 Background safety information 43

9.5 Reporting and collection of serious adverse events 43

9.6 Expedited notification of RAGI 44

9.7 Expedited notification of other relevant safety information 45

9.8 Notification to investigators 45

9.9 Medication errors 45

10.- STATISTICS 45
10.1 Sample size calculation 45

10.2 Statistical analysis 46

10.3 Analysis populations 47

10.4 Security analysis and other secondary results 47

10.5 Intermediate analysis 47

10.6 Independent Evaluation Committee 48

11.- ETHICAL ASPECTS 48
11.1 Informed consent 49

11.2 Data protection 49

11.3 Responsibilities of study participants 50

11.4 Monitoring and auditing 50

11.5 Premature termination or suspension of the study 50

11.6 Documentation of the study 51

12.- FINANCING AND INSURANCE 51
12.1 Financing........ 51

12.2 Insurance 51

13.- PUBLICATION POLICY 51
ANNEX I. BIBLIOGRAPHY 53
ANNEX II: LIST OF PARTICIPATING CENTRES 56
ANNEX III. FLOWCHART 58
ANNEX IV. EUCAST 59
ANNEX V. DIDACTIC MATERIAL 60
ANNEX VI. QUESTIONNAIRE FOR VISITS 3, 4 AND UNPLANNED VISITS 60
ANNEX VII. ALGORITHM FOR ALLOCATING PATIENTS TO PROVEN, PROBABLE OR POSSIBLE RECURRENCE CATEGORIES. 63
ANNEX VIII. PATIENT INFORMATION AND INFORMED CONSENT FORM. 64
ANNEX IX. CHARLSON INDEX 65
ANNEX X. PITT SCORE 66
ANNEX XI. PROCEDURE FOR THE SUBMISSION OF MICROBIOLOGICAL ISOLATES AND FAECAL SAMPLES FOR GUT MICROBIOTA STUDIES. 67
ANNEX XII: ISOLATION FORM 70
ANNEX XIII. AAG NOTIFICATION FORM 71
ANNEX XIV. HELSINKI DECLARATION OF THE WORLD MEDICAL ASSOCIATION 75

2.- GENERAL INFORMATION

2.1 Identification of the clinical trial

Study code: SHORTEN II
EudraCT: 2021-003847-10

2.2 Type of clinical trial

Phase IV, open-label, randomized, pragmatic, multicentre, pragmatic, phase IV clinical trial to demonstrate the superiority of a 7-day *vs.* 14-day licensed antibiotic regimen in the treatment of *P. aeruginosa* bacteraemia.

2.3 Data relating to the promoter

Andalusian Public Foundation for the Management of Health Research in Seville (FISEVI).
Virgen del Rocío University Hospital.
Laboratory Building, 6th floor.
Avda. Manuel Siurot S/N
41013 Seville
Tel. 955012820

2.3.1 Person authorized by the promoter

Dr. Clara M. Rosso Fernández
Clinical Research and Clinical Trials Unit
Virgen del Rocío University Hospital
Avda. Manuel Siurot s/n
41013 Seville
Tel.:955313414
Fax: 954232992

2.3.2 Responsible for Monitoring

Clinical Research and Clinical Trials Unit, Hospital Universitario Virgen del Rocío-SCReN (Spanish Research Network).
Virgen del Rocío University Hospital
Avda. Manuel Siurot S/N
41013 Seville
Tel.:955313414
Fax: 955095338

2.4 Description of the products under study

- **Experimental group (short arm)**: short 7-day regimen with active antibiotic treatment.
- **Control group (long arm)**: 14-day long regimen with active antibiotic treatment.

Active antibiotic treatment shall mean any treatment with proven *in vitro* activity against the strain responsible for the patient's bacteraemia, regardless of the dose administered.

The list of treatments that are considered active according to the MICs of the isolated micro-organism is given in **Table 1** below:

Table 1. List of active treatments according to the MICs of the isolated microorganism.

**Active antibiotic treatments**	**MIC isolated micro-organism**
Ciprofloxacin	MIC ≤0,5 mg/L
Levofloxacin	MIC ≤1 mg/L
Piperacillin/tazobactam	MIC ≤16 mg/L
Ceftazidime	MIC ≤8 mg/L
Cefepime	MIC ≤8 mg/L
Ceftazidime/avibactam	MIC ≤8 mg/L
Ceftolozane/tazobactam	MIC ≤4 mg/L
Imipenem	MIC ≤4 mg/L
Meropenem	MIC ≤ 8 mg/L
Meropenem-vaborbactam	MIC ≤8 mg/L
Colistin	MIC ≤2 mg/L
Amikacin	MIC ≤16 mg/L
Tobramycin	MIC ≤2 mg/L
Aztreonam	MIC ≤16 mg/L

Once the patient is randomized, doses will be optimized if this was not done prior to randomization, according to the EUCAST recommendations reflected in **Table 2:**

Table 2. List of dosage optimized doses of active antibiotic treatments.

Active antibiotic treatments	Dosage-optimized posology
Ciprofloxacin	750 mg/12h oral or 400 mg/8h i.v.
Levofloxacin	500 mg/12h oral or i.v.
Piperacillin/tazobactam	4 g/0.5 g/8h administered as a 4 h extended infusion.<br>4g/0.5 g/6h extended infusion for ceftazidime-resistant strains or pneumonia.
Ceftazidime	2 g/8h i.v.
Cefepime	2 g/8h i.v.
Ceftazidime/avibactam	2 g/8h i.v. administered as a 2 h infusion
Ceftolozane/tazobactam	1 g/0.5 g/8h. For pneumonia, the appropriate dose is 2 g/1g/8h.
Imipenem	1g/ 6h i.v.
Meropenem	MIC ≤2 mg/L: 1g/ 8h i.v.<br>MIC >2 mg/L and ≤8 mg/L: 2g/8h i.v in 3h infusion.
Meropenem-vaborbactam	2 g/2 g every 8 h as a 3 h infusion.
Colistin	4.5 MU/12h i.v.
Amikacin	25-30 mg/kg/24h i.v.
Tobramycin*.	6-7 mg/kg/24h i.v.
Aztreonam	2 g/6h i.v.

* Specifically, aminoglycoside monotherapy shall not be considered as active treatment, except for the treatment of acute pyelonephritis.

2.5 Data from the study investigators

See Annex II

2.6 Laboratories, Medical Department or Related Institutions

Infectious Diseases, Microbiology and Preventive Medicine Clinical Management Unit.
Hospital Universitario Virgen del Rocío/ Instituto de Biomedicina de Sevilla (IBiS)
Avenida Manuel Siurot s/n
41013 Seville
Tel. 955012820

Pharmacy Clinical Management Unit
Virgen del Rocío University Hospital
Avenida Manuel Siurot s/n
41013 Seville
Tel. 955012095

Clinical Research and Clinical Trials Unit, Hospital Universitario Virgen del Rocío-SCReN (*Spanish Research Network*). Virgen del Rocío University Hospital
Avda. Manuel Siurot S/N
41013 Seville
Tel.:955313414
Fax: 955095338

Microbiology Service.
Virgen Macarena University Hospital
Avenida Dr. Fedriani, 3
41009 Seville
Tel. 955926552

CIBER on Infectious Diseases

3.- JUSTIFICATION

Multidrug-resistant (MDR) bacterial infections are one of the greatest threats to public health worldwide (1). For this reason, the European Union and its member states, including Spain, have implemented plans to combat MDR (2,3).

Improving antibiotic use is a key objective of these plans because inappropriate, often excessive and sometimes unjustified antibiotic use is a key risk factor in the development of BMR (2,3).

The Antimicrobial Stewardship Programs (ASP)are one of the most effective interventions to improve antibiotic use (4) and by doing so in an intensive and sustained manner, they reduce bacterial resistance in both hospital (5) and primary care (6).

Administering the correct duration of antibiotic treatment is one of the main areas of improvement for ASPs because it avoids overtreatment and its negative consequences at both the individual and population level. Unfortunately, the optimal duration of antibiotic treatment is still unknown for many of the more common infections. This is because the clinical trials carried out for the approval of available antibiotics were designed for efficacy and safety, not optimal duration.

Therefore, clinical research on the duration of antibiotic treatment is of great interest. Several clinical trials have been carried out in different infectious diseases, including community-acquired pneumonia (7); ventilator-associated pneumonia (8); pyelonephritis (9); intra-abdominal infection (10), post-chemotherapy febrile neutropenia (11) and

Enterobacterial bacteraemia (12, 13, 14) demonstrating that short treatment regimens are not inferior to the traditionally recommended long treatment regimens.

P. aeruginosa infections, and particularly bacteraemia, which is the focus of this project, are common and have a poor prognosis.

P. aeruginosa is among the bacterial species that most frequently cause invasive infections in humans. These infections usually affect patients with comorbidities and healthcare-related situations such as immunosuppressed patients due to any cause: transplant recipients, haematological and oncological patients, major burns, major surgery and patients admitted to intensive care units, and are associated with high mortality, which ranges from 12-60% in various studies on bacteraemias (15). *P. aeruginosa* is a frequent cause of bacteraemia, which is associated with high morbidity and mortality. Therapeutic options for these infections are limited for several reasons: *P. aeruginosa* is intrinsically resistant to most antibiotics used against other Gram-negative bacteria, it frequently develops resistance during treatment (15) and, in addition, there has been an increase in the incidence of multidrug-resistant (MDR) and extensively drug-resistant (XDR) strains in recent years (16).

The duration of antibiotic treatment for bacteraemia caused by *P. aeruginosa* has traditionally responded to experts´ opinion. Different scientific societies propose to treat catheter-related Gram-negative bacteraemias, including *P. aeruginosa*, with a variable duration of 7-14 days (19,20) and there are no indications from other sources. A recent study suggested that shorter courses of antibiotics to treat *P. aeruginosa* bacteraemia may be equally effective as longer courses in terms of risk of 30-day mortality or recurrence. (21). However, the design of this observational study (a retrospective cohort of 249 patients with different treatment durations) is not robust enough to draw conclusions that can be applied to clinical practice.

The risk of recurrence of infection is the argument most widely used to justify the recommendation of prolonged treatment in *P. aeruginosa* infections. It is based on a clinical trial conducted in 401 patients with ventilator-associated pneumonia due to different etiologies which showed that antibiotic treatment for 8 days had comparable clinical efficacy to 14 days in terms of mortality and recurrence, except in pneumonias caused by non-fermenting Gram-negative bacilli, including *P. aeruginosa*, in which recurrence was more frequent in the short treatment group, with equal mortality (8). These results have limitations due to insufficient sample size and the difficulty in distinguishing whether the *P. aeruginosa* isolated from the respiratory secretion sample of the suspected recurrence caused infection or was a colonization of the endotracheal tube.

In *P. aeruginosa* bacteraemia, recurrence is relatively frequent and has a worse prognosis. In a series of 290 patients with *P. aeruginosa* bacteraemia, recurrence occurred in 15 of

episodes within 30 days of treatment discontinuation (21). Recurrence of *P. aeruginosa* bacteraemia was associated with a worse prognosis than the primary episode of bacteraemia (18 vs. 7% mortality) in another series of 441 patients (22). The absence of molecular typing of the isolates is a major limitation of these results that does not allow confirmation of whether the second isolates were recurrences or reinfections. Reinfections may be more common in patients at high risk for *P. aeruginosa* bacteraemia, such as haematological patients with post-chemotherapy neutropenia. The frequency of this is unknown.

The optimal duration of antibiotic treatment of patients with *P. aeruginosa* bacteraemia is unknown. Well-designed clinical trials are needed to find out. The traditional design of non-inferiority clinical trials has limitations in assessing different antibiotic treatment strategies, short *vs.* long course, mainly due to the lack of integration of benefits and harms at the patient level. With this traditional design, efficacy and safety endpoints have been analyzed separately, but this approach may prevent investigators from detecting groups of patients with significant overlapping of these effects (i.e. enhanced efficacy at the expense of increased adverse events) (23).

The DOOR (*Desirability of Outcome Ranking)/RADAR* (*Response Adjusted for Duration of Antibiotic Risk*) design is a novel study design in assessing the risks and benefits of new strategies to optimize antibiotic use to overcome most of these shortcomings.

RADAR is a methodology that uses a superiority design and a 2-step process: First, categorizing patients based on overall clinical outcomes (risk/benefit), and second, ranking patients with according to a ranking of desirable outcomes or DOORs. DOORs are constructed by assigning higher ranks to patients with, first, better overall clinical outcomes and, second, shorter durations of antibiotic use for similar overall clinical outcomes. Antibiotic use strategies are compared through DOOR rankings. The probability that a randomly selected patient will have a better DOOR if assigned a new strategy is estimated.

A limitation of this analysis is that, like other composite variables, the advantage of a strategy in DOOR analysis does not necessarily imply an advantage on all variables; in fact, disadvantages on specific variables are possible.

The DOOR/RADAR method is a significant improvement over previous measures of outcomes achieved by PROAs. It provides an innovative approach to the clinical impact of a given intervention, weighing its efficacy and potential harms on patients in a way that is intuitive for clinicians (23). In addition, its methodology can facilitate the design of superiority clinical trials through more accessible sample sizes (24).

DOOR/RADAR analysis has been used to assess the impact of short course treatment in invasive meningococcal disease (25), intra-abdominal infection (26) and community-

acquired pneumonia (27) but so far no clinical trials have been designed using this innovative analysis.

In the last years, the recent increase in knowledge about the human gut microbiota has changed our view of antibiotics. Thus, antibiotics are no longer considered only beneficial drugs, but also potentially harmful, as their abuse appears to play a critical role in the pathogenesis of several disorders associated with microbiota impairment (e.g. *C. difficile* infection or metabolic disorders) (28). This new insight has triggered studies aimed at understanding how antibiotic class, dose, route of administration and duration of treatment affect the gut microbiota and thus patient outcome (29).

In a recent systematic review, studying how antibiotics induce changes in the human gut microbiota for the most commonly prescribed antibiotics in primary care in the UK (30), they concluded that antibiotics have an impact on the gut microbiota, rapidly reducing bacterial diversity, leading to dysbiosis. After treatment, gut bacteria are capable of some degree of recovery to their baseline state in most individuals, but these studies do not consider recovery in the elderly or other populations. The effect of how bacterial diversity in the intestinal tract impacts on mortality and outcome in several diseases such as cancer or in the follow-up of allogeneic haematopoietic stem cell transplantation has been analysed (31,32), however, there is no study analysing the effect of antibiotic duration on the gut microbiota of patients with *P. aeruginosa* bacteraemia.

The aim of the present trial will be to prove that a 7-day course of antibiotics is better than traditional 14-day schemes for treating patients with *P. aeruginosa* bacteraemia, in terms of reducing patients' antibiotic exposure and thus its side effects, while achieving similar clinical outcomes.

Design rationale

The optimal duration of antibiotic treatment in *P. aeruginosa* bacteraemia is unknown, and no clinical trials have studied it. Therefore, the currently recommended treatment time is based on expert opinion, and is so imprecise that it varies between 7 and 14 days.

Based on data from clinical trials in other bacteria than *P. aeruginosa*, which show that shorter treatment regimens could be as effective and safe as the longer regimens currently recommended, this research project is designed to conduct the first clinical trial to address this important health problem, and to answer the hypothesis that current antibiotic treatments are too long and therefore less safe for the patient, because they are more exposed to their direct side effects, the risk of developing bacteria resistant to the antibiotics used and other superinfections, and finally, because of longer hospital stays.

This clinical trial will use an innovative methodology for the analysis of the results, the so-called DOOR/RADAR, which allows to prove that the optimal duration of antibiotic treatment is associated with the best prognosis for people with this infection.

4HYPOTHESES AND OBJECTIVES OF THE TRIAL

4.1 Hypotheses

In patients with *Pseudomonas aeruginosa* bacteraemia, active antibiotic treatment with a duration of 7 days is superior to 14 days.

Specific hypotheses:

1. The short regimen is better than the long regimen in terms of days without antibiotic treatment and risk of adverse events, superinfections and days of hospital stay.
2. The short regimen is non-inferior to the long regimen in terms of bacteraemia recurrence and mortality, and antibiotic discontinuation can be safely applied on an individual basis following a simple clinical decision rule.
3. Recurrent *P. aeruginosa* bacteraemias are, in some cases, reinfections by other strains than the initial one.
4. The short regimen will have less ecological impact, resulting in less reduction of the bacterial biodiversity of the gut microbiota.

4.2. Objectives

Primary objective: To determine whether a 7-day antibiotic treatment regimen is superior to a 14-day regimen in the treatment of *P. aeruginosa* bacteraemia, assessing in an integrated manner both the effectiveness of the short regimen and its potential to reduce serious adverse events and antibiotic exposure.

Secondary objectives:

1. Determine whether the short regimen is non-inferior to the long regimen in terms of recurrence of infection and mortality, and whether discontinuation of antibiotic treatment can be safely applied at the individual level using a simple clinical decision rule.

2. Describe adverse effects and superinfections, and specifically those caused by multidrug-resistant bacteria and *C. difficile* in both treatment regimens.

3. To analyse the efficiency of short regimens in terms of number of treatment days and days of hospital stay avoided at the end of the follow-up period.

4. Determine risk factors related to treatment failure and risk of recurrences.

5. Confirm recurrence of infections by sequencing *P. aeruginosa* isolates occurring during follow-up.

6. To compare the effect of short and prolonged treatment regimens on the preservation of gut microbiota diversity.

5. TRIAL DESIGN

5.1 Study variables

5.1.1 Main outcome variable

The primary variable will be the probability that any patient in the experimental arm achieves better outcomes than a patient in the control group as assessed through their score in the DOOR / RADAR analysis. This analysis categorizes patients into two steps:

a) A first ordinal classification of clinical outcomes (DOOR), defined by the following mutually exclusive categories, assessed at the end of follow-up:
1. Cure without incident.
2. Cure with proven or probable recurrence.
3. Cure with a serious adverse event.
4. No clinical cure.
5. Death.

b) A second classification in which patients in the same clinical outcome category are classified according to the number of days of antibiotic treatment (RADAR), defined as the number of days of antimicrobial treatment from the time of collection of the first positive blood culture and up to 30 days after discontinuation of trial treatment. Measurement of the number of antimicrobial treatment days shall include all antibiotics and antifungals indicated during that period as empirical or targeted treatment of superinfections occurring during that follow-up period. Antimicrobials indicated as prophylaxis shall not be included.

Therefore, the rank of outcome is established as a priority classification criterion, subordinating the assessment of treatment duration to patients with equal clinical outcome. Thus, patients with a lower DOOR / RADAR score will be those with better outcomes in terms of clinical effectiveness, as well as reduced exposure to antibiotic treatment.

The final result will be expressed as the probability that a patient in the experimental group scored better than a patient in the control group on the DOOR/RADAR scale. The results will be considered favourable for the intervention studied if this percentage exceeds 50%.

5.1.2 Secondary variables.

Table 3. List of secondary study variables and their follow-up period.

Variable	Monitoring period
Treatment failure	Day +30 from discontinuation of trial treatment.<br><br>Day +90 from the date of extraction of the first positive blood culture.
Analysis DOOR	Day +30 from discontinuation of trial treatment.<br><br>Day +90 from the date of extraction of the first positive blood culture.
All-cause mortality	Day +30 from discontinuation of trial treatment.<br><br>Day +90 from the date of extraction of the first positive blood culture.
Clinical cure	Day +30 from discontinuation of trial treatment.<br><br>Day +90 from the date of extraction of the first positive blood culture.
Proven, probable or posible relapses	Day +30 from discontinuation of trial treatment.<br><br>Day +90 from the date of extraction of the first positive blood culture.
New episode of bacteraemia	Day +30 from discontinuation of trial treatment.<br><br>Day +90 from the date of extraction of the first positive blood culture.
Recurrence of fever	Day +30 from discontinuation of trial treatment.

	Day +90 from the date of extraction of the first positive blood culture.
Superinfections	Day +30 from discontinuation of trial treatment.<br><br>Day +90 from the date of extraction of the first positive blood culture.
Serious adverse events	Day +30 from discontinuation of trial treatment.<br><br>Day +90 from the date of extraction of the first positive blood culture.
Antibiotic-free days	Day +30 from discontinuation of trial treatment.<br><br>Day +90 from the date of extraction of the first positive blood culture.
Days of hospitalization	Day +30 from discontinuation of trial treatment.<br><br>Day +90 from the date of extraction of the first positive blood culture.
Recovery of baseline functional capacity	Day +30 from discontinuation of trial treatment.<br><br>Day +90 from the date of extraction of the first positive blood culture.

5.2 Design

Phase IV, open-label, randomized, multicentre, pragmatic, clinical trial to demonstrate the superiority/non-inferiority of a 7-day *vs.* 14-day licensed antibiotic regimen in the treatment of *P. aeruginosa* bacteraemia.

a. Experimental group (short arm): will receive seven days of active antibiotic treatment from the last *P. aeruginosa* positive blood culture as long as the clinical decision rule for treatment discontinuation is met.

b. Control group (long arm): will receive 14 days of active antibiotic treatment from the last *P. aeruginosa* positive blood culture as long as the clinical decision rule for treatment discontinuation is met.

5.3 Randomization procedure

Randomization will be performed on day 6 (+/- 24h) from the start of active antibiotic treatment.

Patients who prior to the time of randomization had received active treatment at a dose that was not optimized according to EUCAST criteria may be included in the trial. In this case, the dose will be optimized at the time of randomization, without the need to extend the total duration of treatment foreseen in the trial.

Patients will be detected from the daily review of blood culture results. In those patients with isolation of *Pseudomonas aeruginosa* in it, the inclusion and exclusion criteria of the study will be checked and informed consent will be requested. Patients who meet all the inclusion criteria but have some exclusion criteria will be considered to have failed *screening*, and the reason for exclusion will be recorded in the corresponding case report form.

To facilitate the inclusion of patients in the study, the following procedure will be followed.

1. Daily identification of all *P. aeruginosa* positive blood cultures by the Microbiologist responsible for the study at each site.
2. Clinical assessment of patients identified with *P. aeruginosa* bacteraemia by the clinician responsible for the study to see if the patient meets the inclusion and exclusion criteria.
3. Patients who meet the inclusion criteria and have no exclusion criteria will be asked for informed consent by the clinician responsible for the study at each centre.
4. Patients who meet the inclusion criteria but have any exclusion criteria will be considered to have failed *screening* and the reason for exclusion should be recorded.
5. Patients who do not meet the inclusion criteria will not be included in the trial. The criterion(s) they do not meet should be collected.

An antibiogram of the isolate must be available based on one of the methods recommended by EUCAST.

Once the informed consent has been signed and all eligibility criteria have been assessed, randomization will be performed on day 6 from the start of antibiotic treatment (+/-24h), in a 1:1 ratio, to the experimental arm or to the control arm. Randomization will be simple and stratified by centre and by pulmonary/extrapulmonary origin for bacteraemia (see details in flowchart, (Annex III).

A randomization list will be generated using Epidat 4.0 software and integrated into the case report form (eCRF), so that only after the registration of all eligibility criteria will the allocation of the arm to which the patient belongs be revealed to the investigator, as well as a code to identify the patient, composed of a numerical code identifying centre and patient (e.g. XX-XX).

Only the staff in charge of this trial in the Clinical Trials Unit and the IT support team responsible for the eCRF will have access to the randomization list.

5.4 Blinding

An open-ended design has been chosen for pragmatic reasons; in addition, the masking of a large number of different drugs that would have to be carried out would be logistically unfeasible.

As there is no masking, no blinding procedure is applicable either.

The following measures have been put in place to avoid as far as possible the bias that the open-label nature of the trial might generate:

- Objective classification criteria have been established for the assessment of primary and secondary variables.
- The assessment of outcome variables will be carried out by a blinded independent committee (not taking part in the study as investigators) with respect to treatment allocation. This committee will consist of 3 expert investigators belonging to CIBER Infectious Diseases and will reach its conclusions by consensus. This independent assessment is essential in the only outcome indicator that is not objective: the diagnosis of probable recurrence. Finally, statistical analyses of the results obtained will be carried out on a blinded dataset in which data relating to allocation to treatment arms will be removed.

5.5 Study treatments

a. Experimental group (short arm): will receive seven days of active antibiotic treatment from the last positive blood culture as long as the clinical decision rule for treatment discontinuation is met.

b. Control group (long arm): will receive 14 days of active antibiotic treatment from the last *P. aeruginosa* positive blood culture as long as the clinical decision rule for treatment discontinuation is met.

Active antibiotic treatment is considered to be any treatment with proven *in vitro* activity according to the EUCAST recommendations against the strain responsible for the patient's bacteraemia, regardless of the dosage or form of administration (**Table 3**).

Once the patient is randomized, treatment will be optimized where necessary, as defined in the list in **Table 4**, according to EUCAST recommendations (Annex IV).

Specifically, aminoglycoside monotherapy will not be considered appropriate except for the treatment of acute pyelonephritis.

(a) In the **experimental group (short arm)** the duration of active antibiotic treatment will be 7 days from the date of collection of the last positive blood culture, provided that there are no symptoms and signs of infection within the previous 72 hours. During this period of time, the initial antibiotic may be modified (simplification of the empirical treatment after obtaining the antibiogram results, continuation of intravenous treatment to oral treatment, modification of the agent due to adverse events, etc.) provided that the new antibiotic is an appropriate therapeutic option for the treatment of the infection in question. If the patient does not meet the clinical criteria for withdrawal, he/she should be re-evaluated at successive intervals of 48-72h until it is documented that he/she meets these criteria, and then treatment should be discontinued.

(b) In the **control group (long arm),** the total duration of active antibiotic treatment will be 14 days from the last positive blood culture, provided that there were no symptoms and signs of infection in the previous 72 hours. During this period of time, the initial antibiotic agent may be changed to a different one (due to simplification of the empirical treatment after obtaining the results of the antibiogram, continuation of intravenous treatment to oral treatment, modification of the agent due to adverse effects, etc.) provided that the new agent is an appropriate therapeutic option for the treatment of the infection in question.
If the patient does not meet the clinical criteria for withdrawal, he/she should be reassessed at successive intervals of 48-72 hours until it is documented that he/she meets these criteria, and treatment should then be discontinued.

Patients who prior to the time of randomization had received active treatment but at doses that were not optimized according to EUCAST criteria may be included in the trial. In this case, the dose will be optimized at the time of randomization, and they will not need to extend the total treatment duration foreseen in the trial arm, provided they meet the clinical requirements set by the trial for treatment interruption.

Antibiotic treatment may be resumed after this point if the physician in charge of the patient deems it necessary due to unfavorable evolution. The route and form of antibiotic administration, criteria for hospital discharge or clinical management of possible complications will be at the discretion of the physician in charge of the patient, following usual clinical standards and local antibiotic management policies.

Continuation of intravenous treatment by intravenous home antimicrobial therapy programmes is allowed for patients who, in the judgement of their responsible physician, meet stability criteria for outpatient management.

Prior to the start of the trial and during the trial, participating centres will receive educational material on the optimal use of antibiotics in patients with invasive infections caused by *P. aeruginosa*, according to the best available evidence (Annex V).

Control blood culture: blood culture taken at the time of patient inclusion, and taken successively every 48-72 hours if the blood cultures remain positive, until a negative blood culture is obtained. Control blood cultures will also be taken whenever the patient presents symptoms or clinical signs of a possible recurrence of the infection, or whenever the patient's clinical situation so requires at the discretion of the responsible physician.

5.6 Allowed medicines and treatments

The use of any of the antibiotics commonly used in routine clinical practice for the treatment of *P. aeruginosa* bacteraemia is permitted, with the usual doses and administration schedules described in the technical data sheet or recommended by local and international scientific societies in each case (**Table 1**).

There are no restrictions on the use of other medicines, however, only antibiotic therapy will be included as concomitant medication.

5.7 Follow-up of patients

For patients with hospital admission criteria, clinical follow-up will be performed daily by their responsible physician(s) while they remain hospitalized, and by the Clinical Investigator responsible for the Trial at the Centre and when deemed necessary by the Clinical Trial Coordinator. The randomization, end-of-treatment and visit 2 visits will necessarily be face-to-face. For patients without hospital admission criteria, visits 3 and 4

may be conducted by telephone call on days +30 from discontinuation of trial treatment and +90 from extraction of the first positive blood culture, using a structured questionnaire (Annex VI).
Follow-up of patients will be performed at the visits defined in this protocol and will continue until day +90 after the first positive blood culture is taken. The schedule of visits is specified in **Table 4** and in section 8.4.

5.8 Study termination or discontinuation criteria

Premature discontinuation of the clinical trial can occur due to a decision by the regulatory authorities, a change in the opinion of the Clinical Research Ethics Committees, safety and/or drug safety issues, or indications of inefficacy.

A pre-defined stopping rule has been established for the interim analysis, which will be performed after 40% of the sample size has been recruited. The trial will be conducted by an independent assessment committee that will have full access to all study data (see section 2.6.1). The potential futility of the trial will be assessed by means of the conditional power (CP) for the secondary endpoint based on the trend observed in the interim analysis, as well as the projected one if it is met (see section 2.6.2.) a baseline scenario. A PC of <15% is considered unlikely to demonstrate treatment efficacy, and therefore discontinuation of the trial will be considered in conjunction if crosses this margin. In addition, any difference in mortality between the groups will be carefully assessed.

The investigator as well as the sponsor reserve the right to discontinue the study at any time for reasonable medical and/or administrative reasons.

5.9 Procurement, packaging and labelling of medication

Not applicable as the medicines to be used in this study will be those that are dispensed on a daily basis in the Pharmacy Services of the participating hospitals.

5.10 Storage and dispensing of medication

Medication shall be stored in the Pharmacy Service of each centre under the storage conditions specified by the manufacturer for such products.

Dispensing will be carried out according to the standardized procedures of each participating centre, always keeping a control of administration. A dispensing register will be included in the study documentation to ensure the traceability of the products administered, noting the active ingredient, batch, expiry date and number of units administered.

5.11 End of trial

The day of the final visit of the last patient included in the study is considered the end of the trial.

6.- SELECTION CRITERIA

Adult patients with monomicrobial bacteraemia (blood culture isolation) due to *P. aeruginosa.*

6.1 Inclusion criteria

1. Adult patients (18 years and older).
2. Who present with *P. aeruginosa* bacteraemia.
3. Who have received 6 days (+/-1) of active antibiotic treatment against bacteraemia active antibiotic treatment against bacteraemia counted from the date of the first positive blood culture until the time of randomization.
4. Have signed the informed consent form for the trial.

Note: Inclusion of the same patient more than once is not allowed.

6.2 Exclusion criteria

1. Under 18 years of age.
2. Pregnant or breastfeeding women. Potentially fertile patients should have a negative pregnancy test.
3. Bacteraemia source not adequately controlled at least 72h before randomization at least 72h prior to randomization.
4. Bacteraemia secondary to an infection necessarily requiring prolonged antibiotic treatment, greater than 7 days, including:
    - Post-obstructive or necrotising pneumonia
    - Lung abscesses
    - Acute prostatitis
    - Bone and joint infections
    - Infections of the central nervous system
    - Endovascular infections related to vascular prostheses.
    - Any other at the discretion of the physician responsible for the patient.

5. Coexistence of a different infection at the time of diagnosis of bacteraemia also requiring antibiotic treatment antibiotic treatment.
6. Bacteremic pneumonia in severely immunocompromised patients, defined as:
   - Patients with severe neutropenia (<500 cells / mm3)
   - Recipients of allogeneic hematopoietic stem cell or solid organ transplantation, during the first year after the transplantation
   - Active graft-versus-host disease requiring immunosuppressive treatment
   - Patients with solid tumours undergoing chemotherapy
   - Untreated HIV infection with CD4 < 200 cells / mm3
   - Patients with primary combined immunodeficiency
   - Steroid treatment with prednisone > 20 mg/day (or equivalent) for 14 days prior to randomization
7. Bacteraemia of any origin in patients with severe neutropenia (<500 cells/mm3) at randomization.
8. Bacteraemia of any origin in major burns.
9. Bacteraemia caused by strains resistant to all beta-lactams and quinolones.
10. Polymicrobial bacteraemia including microorganisms other than *P. aeruginosa.*
11. Patients in palliative care o with a survival expectancy of less than 48h at the time of randomization at the time of randomization.
12. *P. aeruginosa* bacteraemia in the previous 90 days.
13. The physician responsible for the patient does not want to include the patient in the clinical trial.

6.3 Withdrawal criteria

6.3.1 By decision of the patient

In accordance with the Declaration of Helsinki, patients have the right to withdraw from the study at any time and for any reason, either personally or through their representative.

6.3.2 Loss of tracking

Due to loss of patient follow-up, a patient will be considered lost to follow-up when the patient does not attend the end-of-treatment visits (Day 7+/-2 after initiation of active antibiotic treatment in the experimental arm and Day 14+/-2 after initiation of active antibiotic treatment in the control arm) and the test-of-cure visit (Day +30 from discontinuation of trial treatment). Regardless, data from these patients will be included in the intention-to-treat analysis.

6.3.3 By security criteria

The occurrence of an adverse event or any other reason that, in the judgement of the clinician, requires the withdrawal of the antibiotic in use because the treatment is not considered safe for the patient, may be life-threatening, or may have serious consequences for the patient.

6.3.4 For non-compliance or violation of the rules contained in the protocol

When the patient no longer complies with the trial standards, he/she may be withdrawn at the discretion of the responsible investigator or due to loss to follow-up.

6.4 Follow-up of prematurely withdrawn patients

If a patient is prematurely withdrawn from the trial, the investigator will provide the primary reason for the withdrawal and, as indicated in the GCP guidelines, procedures will be followed according to standard treatment protocols for the patient's condition at the discretion of the responsible clinician.

6.5 Definitions

Active treatment: Any treatment with proven *in vitro* activity against the strain responsible for the patient's bacteraemia according to EUCAST criteria (34), regardless of the dose used.
Specifically, aminoglycoside monotherapy will not be considered active except for the treatment of acute pyelonephritis.
The list of active treatments is shown in **Table 1**.

Optimized treatment: Treatment with proven *in vitro* activity and administered at doses optimized according to EUCAST recommendations (34).
The list of optimized treatments is shown in **Table 2.**

Patients who prior to the time of randomization had received active treatment but at doses that were not optimized according to EUCAST criteria may be included in the trial provided they meet the clinical improvement criteria for randomization. In this case, the dose will be optimized at the time of randomization, and it will not be necessary to extend the planned treatment time in their group for this reason, provided they meet the clinical safety requirements established by the trial for treatment discontinuation.

Death: Death of the patient from any cause.

Proven relapses: It consists of three different situations:

a) Recurrence of symptoms and signs of the infection responsible for inclusion in the trial after discontinuation of treatment, in a patient whose symptoms had resolved, with re-isolation of *P. aeruginosa* from blood cultures or representative samples from the site of infection.

b) Patients with recurrent isolates of *P. aeruginosa* in microbiological samples without clinical signs and symptoms of new-onset infection are specifically excluded from this category, as these isolates should be considered as colonisations.

c) Patient who develops during follow-up a distant haematogenous infection attributed to the infection that led to inclusion in the trial (spondylodiscitis, osteoarticular or prosthetic material infection, pulmonary embolisms, etc.), in which the presence of P. aeruginosa in a representative sample from the new site of remote infection.

Probable relapse: Comprises three different situations:

a) Patients with relapse of local symptoms and signs of the infection that prompted inclusion in the trial after discontinuation of treatment, in a patient whose symptoms had resolved, but in whom no etiological agent responsible for the symptoms is isolated (due to negative or uncollected cultures).

   Patients with recurrence of symptoms and signs related to the initial site of infection, for which an etiology other than *P. aeruginosa* is determined in cultures are specifically excluded from this category (to be coded as superinfections).

b) Patients with recurrence of *P. aeruginosa* bacteraemia without any symptoms or focal signs in whom a source for the new episode of bacteraemia is not demonstrated.

   Patients who have a new episode of *P. aeruginosa* bacteraemia in which an alternative and distinct source to the one for which they were included in the trial is demonstrated are specifically excluded from this category (to be coded as "new episode of bacteraemia").

c) Patient who develops during follow-up a distant haematogenous infection attributed to the infection that led to inclusion in the trial (spondylodiscitis, osteoarticular or prosthetic material infection, pulmonary embolisms, etc.), where *P. aeruginosa* is not documented because no representative sample from the new distant site of infection is available, or because no samples from this site of infection are available.

   Patients in whom such complications are suspected, but in whom microbiological samples from the new site of infection confirm an alternative etiology for the infection other than P. aeruginosa are specifically excluded from this category (to be coded as superinfections). P. aeruginosa are specifically excluded from this category (to be coded as superinfections).

Being a subjective outcome variable, the assessment of the outcome variable will be made by the external evaluation committee, which is unaware of the assignment of the case to the experimental or control group.

Possible relapse: Recurrence of fever after discontinuation of treatment in a patient whose symptoms had already resolved, without symptoms specific to the site of infection responsible for inclusion in the trial, and without any other defined alternative cause for the fever.

The ascription of patients to the categories of proven, probable or possible recurrence is provided in the algorithm in Annex VII.

Treatment failure: Death from any cause or proven or probable recurrence during follow-up.

Clinical cure: Patient alive, with resolution of fever and signs and symptoms of infection responsible for inclusion in the trial and negative control blood cultures at follow-up.

Control of the source: The performance of interventions aimed at eliminating or draining the source responsible for the bacteraemia, including the removal of vascular catheters, urinary or biliary tract diversions, surgical debridement of soft tissues, surgical or percutaneous drainage of deep abscesses, etc. In the case of urinary tract infections in patients with indwelling urinary catheters, removal of the catheter is not mandatory in order to consider the source properly controlled.

Superinfection: infections of any other etiology, including other Gram-negative bacilli other than *P. aeruginosa* including other Gram-negative bacilli, *Enterococcus* sp., *Staphylococcus* sp, *C. difficile* infection and fungal infections by yeast or filamentous fungi, during follow-up.

Specifically, microbiological cultures obtained from non-sterile sites (urine, skin, respiratory tract) that do not correlate with a clinical syndrome of infection will be interpreted as colonisations and not as superinfections.

New episode of bacteraemia: new isolation of P. aeruginosa in blood cultures from any cause after discontinuation of treatment in a patient who had already demonstrated a negative blood culture after the initial episode that led to inclusion in the trial.

Recurrence of fever: return of fever after reaching apyrexia (temperature ≤37°C) for at least 72h.

Days free of antibiotic treatment: total number of days free of antibiotic treatment. For this purpose, the days on which at least one dose of antimicrobial treatment (antibiotic or antifungal) is received for any indication (including antibiotic prophylaxis) shall be subtracted from the total number of days observed for each patient.

Days of hospitalization: days from the start of treatment to hospital discharge. All days of hospital stay observed during follow-up will be added together regardless of whether they are consecutive or not.

Serious adverse events: the number of serious adverse events shall be collected and presented as a rate per 1,000 patient-days and as a proportion of patients with at least one serious adverse event.

For the definition of serious adverse events, please refer to the latest version of the protocol.

Recovery of basal functional capacity: return to the self-referred activity that the patient was able to perform before the episode of bacteraemia.

Multidrug-resistant *P. aeruginosa*: according to the recent proposal of the European Centre for Disease Prevention and Control Disease Control of the European Centre for Disease Prevention and Control, we define a multidrug-resistant pathogen as one that is resistant to at least one agent from ≥3 classes of antimicrobial agents (35).

Extensively resistant *P. aeruginosa*: According to the recently proposed European Centre for Disease Prevention and Control European Centre for Disease Prevention and Control, we define a multidrug-resistant pathogen as one that is resistant to any one agent in all but one or two classes of antimicrobial agents (35).

7.- TREATMENT OF SUBJECTS

7.1 Concomitant medication

The use of any of the antibiotics commonly used in routine clinical practice for the treatment of *P. aeruginosa* bacteraemia is permitted, with the usual doses and administration schedules described in the technical data sheet or recommended by local and international scientific societies in each case (**Table 1**).

There are no restrictions on the use of other medicines, but only antibiotic therapy is listed in the CRD as concomitant medication.

7.2 Rescue medication

The use of rescue medication is not foreseen. If a patient is withdrawn due to lack of efficacy or a similar condition, he/she will be treated according to clinical guidelines and standard clinical practice for these cases.

7.3 Schedule of visits and evaluations

Table 4. Schedule of visits and procedures.

**Procedures**	**Visit 0 selection and randomization**	**Visit 1 End of treatment**	**Visit 2 Follow-up**	**Visit 3 Healing test**	**Visit 4 End of Follow-up**	**Unplanned visit**
**Days**	**Day 0** (Day 6º +/-1) of active antibiotic treatment)	**Day +7 (+/-1) or +14 (+/-1)** since start of active treatment	**Day +7 +/-48h** since end of active treatment	**(Day +30 +/-2)** from discontinuation of trial treatment Telefónica*.	**Day +90 +/-2)** since the first positive blood culture Telefónica*.	
**Randomization**	X					
**Informed consent**	X					
**Inclusion/exclusion criteria**	X					
**Pregnancy test**[1]	X					
**Medical history / anamnesis**	X	X	X	X	X	X
**Demographic data**	X					
**Physical examination and vital signs**	X	X	X			X
**Symptoms of infection**	X	X	X	X	X	X
**Antibiotic treatment**	X	X				X
**Haematology/ biochemistry**	X	X				X

**Blood culture**	X[3]					X
**Microbiological data[2]**	X	X	X	X	X	X
**Faecal samples**	X	X		X	X	
**Suspension of antibiotic treatment**		X				
**Concomitant medication**	X	X	X	X	X	X
**Adverse events**		X	X	X	X	X

1 If applicable. To be performed on blood or urine
2 Review of blood cultures and cultures to identify isolation of P. aeruginosa and potential superinfections.
3 If the first control blood culture is positive, a new blood culture shall be taken at the time of receipt of the positive report. In case this first blood culture is negative, no further determination shall be necessary unless clinical judgement is made.

*If the patient participates in the study of bacterial diversity in the intestinal microbiota, the visit will take place in person.

7.4 Procedures per visit

Visit 0 (Selection and randomization)

It shall be performed **on the 6th day (+/- 1) of active antibiotic treatment** and the following consecutive procedures shall be carried out.

1. Verification of inclusion/exclusion criteria. Selection failures are defined as subjects who are candidates for the trial but after checking inclusion/exclusion criteria fail to meet any of them. This should be recorded in a database separate from the CRD.
2. Signing of informed consent. The nature of the study as well as the potential risks and benefits associated with the trial will be explained to the patient in detail. Patients will sign the informed consent form (Annex VIII) or, failing that, the legal representative.
3. Data collection:
    - Demographic data: date of birth, age, gender, date of inclusion.
    - Medical history, including personal history: drug allergies, previous comorbidities, antibiotic treatments administered, current reason for admission, etiological diagnosis of the infection and possibilities of controlling or eradicating the outbreak within 24 hours when possible, baseline symptoms and date of onset, antibiotic treatment received during hospitalisation, Charlson index (Appendix IX) and Pitt score (Appendix X). This information is described in detail in the data collection notebook.
    - Physical examination including vital signs (blood pressure, heart rate, respiratory rate, body temperature, oxygen saturation) and examination by organs and apparatus (cardiorespiratory auscultation, abdominal palpation,

examination of limbs and skin, including inspection of vascular, urinary or biliary catheters if present).
- Review of concomitant medication.

4. Laboratory determinations:
   - Collection of baseline analytical data. The analytical data obtained in the blood determinations relevant to the infectious process of the patient that motivates their admission or attendance at consultations may be recovered. A maximum variability of +/- 48 hours of the blood draw with respect to the date of the visit is allowed, and the data from the determination closest to the date of the visit must be collected. The data collected include basic plasma biochemistry (glucose, creatinine, urea, sodium, potassium, liver biochemistry), C-reactive protein and a basic haemogram.
   - If the patient to be included is a woman of childbearing age, a pregnancy test will be performed at this visit.

5. Blood culture collection at the time of randomization. If it is positive, it shall be repeated; if negative, no further tests are necessary unless better clinical judgement is reached.

Visit 1 (End of treatment)

Experimental arm: (Day 7+/-1 after initiation of active antibiotic treatment).

1. Data collection:
   - Medical history, including the presence of signs or symptoms of infection and the date of resolution, confirmation of adequate control of the source of infection, antibiotic treatments administered, etc. This information is described in detail in the data collection notebook.
   - Physical examination including vital signs (blood pressure, heart rate, respiratory rate, body temperature, oxygen saturation) and examination by organs and apparatus (cardiorespiratory auscultation, abdominal palpation, examination of limbs and skin, including inspection of vascular, urinary or biliary catheters if present).
   - Collection of adverse events, including adverse drug reactions, occurrence of superinfections by resistant microorganisms, or *Clostridioides difficile* diarrhoea, as described in the data collection notebook.
   - Collection of concomitant medication.
2. Laboratory determinations:
   - Analytical data collection.

Suspension of antibiotic treatment if the patient belongs to the experimental group. Prior to discontinuation of antibiotic treatment, it should be confirmed that the patient meets all the necessary requirements:

- Apirexia (temperature ≤37ºC) for at least 72h.
- Resolution of symptoms and signs attributable to infection for at least 72h.

If the patient meets these requirements, antibiotic treatment will be discontinued for patients in the experimental group.

If the patient does not meet either of these requirements, the patient shall be reassessed again after 48-72 h, repeating the systematic assessment of these two criteria, and so on.

In patients assigned to the experimental arm, the procedures designated for visit 0 and 1 can be performed on the same day, unifying them into a single visit.

Control arm (Day 14 +/- 1 day after initiation of active antibiotic treatment).

1. Data collection:
    - Medical history, including the presence of signs or symptoms of infection and the date of resolution, confirmation of adequate control of the source of infection, antibiotic treatments administered, etc. This information is described in detail in the data collection notebook.
    - Physical examination including vital signs (blood pressure, heart rate, respiratory rate, body temperature, oxygen saturation) and examination by organs and apparatus (cardiorespiratory auscultation, abdominal palpation, examination of limbs and skin, including inspection of vascular, urinary or biliary catheters if present).
    - Collection of adverse events.
    - Collection of concomitant medication.
2. Laboratory determinations:
    - Analytical data collection.

Suspension of antibiotic treatment if the patient belongs to the control group. Before discontinuing antibiotic treatment, it must be confirmed that the patient meets all the necessary requirements:

- Apirexia (temperature ≤37ºC) for at least 72h.
- Resolution of symptoms and signs attributable to infection for at least 72h.

If the patient meets these requirements, antibiotic treatment will be discontinued for patients in the experimental group.

In the event that the patient does not meet any of these requirements, the patient shall be reassessed again after 48-72 hours, repeating the systematic assessment of these three criteria, and so on.

Visit 2 (Follow-up, day +7 +/- 48h from the date of discontinuation of treatment).

1. Data collection:
    - Medical history, including the presence of signs or symptoms of infection and the date of resolution, confirmation of adequate control of the source of infection, antibiotic treatments administered, etc. This information is described in detail in the data collection notebook.
    - Physical examination including vital signs (blood pressure, heart rate, respiratory rate, body temperature, oxygen saturation) and examination by organs and apparatus (cardiorespiratory auscultation, abdominal palpation, examination of limbs and skin, including inspection of vascular, urinary or biliary catheters if present).
    - Collection of adverse events.
    - Collection of concomitant medication.
    - Microbiological data collection (review of blood cultures and cultures to identify isolation of *P. aeruginosa* or superinfections).

Visit 3 (test of cure, day 30 +/-48h after discontinuation of antibiotic treatment): to be carried out by telephone using a structured questionnaire (Annex VI)**.**

1. Data collection:
    - Collection of clinical data, including the presence of signs or symptoms of infection, antibiotic treatments administered, etc. as detailed in the data collection notebook.
    - Collection of adverse events.
    - Collection of microbiological data (review of blood cultures and cultures to identify isolation of *P. aeruginosa* or superinfections).

Visit 4 (end of follow-up, day 90 +/48h from the date of collection of the first positive blood culture): to be carried out by telephone using a structured questionnaire (Annex VI)**.**

2. Data collection:
    - Collection of clinical data, including the presence of signs or symptoms of infection, antibiotic treatments administered, etc. as detailed in the data collection notebook.
    - Collection of adverse events.
    - Collection of microbiological data (review of blood cultures and cultures to identify isolation of *P. aeruginosa*).

7.5 Other Study Procedures (Sub-Studies)

7.5.1 Microbiological studies

The processing of positive blood cultures shall be carried out according to the procedures established in each participating centre. Identification of *P. aeruginosa* isolates shall preferably be performed by mass spectrometry, and identification to genus and species level shall be considered as certain when the *score value* of the P. *aeruginosa isolate* is higher than that of the P. *aeruginosa isolate.* The antibiotic sensitivity study will be carried out using systems that allow the MIC value to be obtained for the different antibiotics (commercial microdilution or MIC gradient strips) and must be interpreted based on EUCAST criteria (*The European Committee on Antimicrobial Susceptibility Testing. Breakpoint tables for interpretation of MICs and zone diameters*. Version 11.0, 2021) .

All *P. aeruginosa* isolates recovered from blood cultures (or from other clinical samples of interest) will be kept frozen (preferably at -80ºC) for shipment to laboratory 208 of the Instituto de Biomedicina de Sevilla IBiS (Annex XI). There they will be recorded and sent to the corresponding centre according to each of the objectives of the different sub-studies.

The rules for taking and processing samples are detailed in Annex XI. The isolates shall be submitted together with the form included in Annex XII.

7.5.2 Studies of bacterial diversity in the gut microbiota.

Bacterial gut microbiota diversity from patients with *P. aeruginosa* bacteraemia at baseline and at different time points will be collected from selected hospitals in either the experimental arm (group 1, 7 days of treatment) or the control arm (group 2, 14 days of treatment). Stool samples from patients in each group 1 and 2 will be collected at visit 0 (randomization), visit 1 (end of treatment), visit 3 (test of cure) and visit 4 (end of follow-up). For each group, 20 samples will be obtained from patients receiving treatment with beta-lactams with spectrum of activity for anaerobic bacilli (piperacillin / tazobactam and carbapenems), from 20 samples from patients receiving treatment with beta-lactams with no activity against anaerobes (ceftazidime and carbapenems). (ceftazidime and cefepime) and 20 samples from patients receiving ciprofloxacin.

8.- ASSESSMENT OF EFFECTIVENESS

8.1 Efficiency variables

Main: Analysis DOOR/RADAR at day +30 from trial treatment discontinuation.

Secondary:
Secondary non-inferiority variable Treatment failure at day +30.

Other secondary variables treatment failure on day +90, all-cause mortality, clinical cure, analysis DOOR, superinfections, days of hospital stay and recurrences (proven, probable or possible)recurrence of fever, new episode of bacteraemia, recovery of baseline functional capacity on days +30 and +90. Total number of days of antibiotic free of antibiotic treatment and days of hospitalization at day +90.

8.2 Laboratory tests

Blood tests will be performed at the initial visit and thereafter as specified in the visit schedule (**Table 4**). These tests will be performed locally at each centre in accordance with standard clinical practice. The following tests will be performed:

- Blood count including leukocyte, neutrophil, haemoglobin and platelet counts.
- Blood biochemistry including determination of sodium, potassium, creatinine, urea, C-reactive protein, AST (GOT), ALT (GPT) and total bilirubin (if altered also direct bilirubin).

9.- SAFETY ASSESSMENT

9.1 Security assessments

The following clinical evaluations will be conducted to assess the safety profile of the trial:

9.1.1 Physical examination, vitals

Physical examination and vital signs. Vital signs (blood pressure, heart rate, respiratory rate, body temperature, oxygen saturation) and examination by organs and apparatus (cardiorespiratory auscultation, abdominal palpation, examination of limbs and skin, including inspection of vascular, urinary or biliary catheters if present) will be carried out at each visit.

9.1.2 Laboratory and microbiological tests

A basic haemogram, biochemistry and serial blood cultures will be carried out according to the schedule of procedures. Microbiological studies will also be carried out to identify pathogenic strains.

9.2 Adverse events of interest for follow-up

Incidence of adverse events.

- Diarrhoea: subjects who experience diarrhoea (3 or more stools per day of decreased consistency) during the study will be asked to detect *C. difficile* toxin in stool. Whether positive or negative, it will be recorded as an adverse event (AE) but will not result in discontinuation of study treatment unless deemed necessary by the PI.
- Nausea, vomiting.
- Headache.
- Seizures.
- Liver toxicity (elevated transaminases and/or bilirubin).
- Haemolytic anaemia and other haematological disorders (leukopenia, thrombopenia).
- Coagulation disorders.
- Alterations in sodium and potassium levels.
- Hypersensitivity reactions and skin rash.

9.3 Definitions

Adverse Event (AE):

An adverse event is any undesirable medical reaction experienced by the patient at any time during the course of the study, whether or not it is considered to be related to the study treatment. This definition includes the development of a new disease and exacerbation of pre-existing conditions other than the indication under study.

Adverse Reaction (AR):

An AR is any unintended, noxious reaction to an investigational medicinal product, regardless of the dose administered.

Serious Adverse Event (SAE) and Serious Adverse Reaction (SAR):

Serious AEs or ARs are considered to be those which, at any dose, may result in death, threaten the life of the subject, require hospitalization of the patient or prolong an existing hospitalization, cause permanent or significant disability or incapacity, or result in a congenital anomaly or malformation. Suspected AE or AR that is medically significant, even if it does not meet the above criteria, including major medical events that require intervention to prevent one of the consequences described above, are also considered serious. In addition, all suspected transmission of an infectious agent via a medicinal product shall be reported as serious.

The concept of "threatening the life of the subject" in the definition refers to the fact that, in the opinion of the investigator, the patient at the time of the AA or AR is at real risk of death; it does not refer to the fact that the AA/AR hypothetically could have resulted in death had it been more intense.

The term "requiring hospitalization" shall exclude both planned hospitalizations for scheduled treatments and hospitalizations that have been planned or are planned before the start of the study in relation to a pre-existing medical condition.

The following adverse events will be recorded in the CRD of the study and will be evaluated as part of the safety variables, but due to their expected nature within the evolution of the pathology under study, **no specific notification will be made to the VF department (it will not be necessary to complete the SAES form):**

- Proven or probable relapse meeting serious adverse event criteria.
- Superinfection meeting serious adverse event criteria.

See definitions in section 7.2

Unexpected Adverse Reaction (UAR):

Any AR whose nature, intensity or consequences do not correspond to the reference safety information.

Serious Unexpected Adverse Reaction (SUAR):

RAG (previously defined), the nature, severity or consequences of which do not correspond to the reference safety information.

Causality Criteria:

- Related AA: The temporal relationship of the AA with the study medication indicates a possible causal relationship and cannot be explained by factors such as the patient's clinical condition, therapeutic interventions.
- Unrelated AA: The temporal relationship of the AA to the study medication indicates an unlikely causal relationship, or other factors (medication or concomitant conditions), other therapeutic interventions provide a satisfactory explanation for the AA.

9.4 Background safety information

Data sheets of all antibiotics with anti-pseudomonal activity with authorized indication shall be included in the investigator's file of each participating centre.

9.5 Reporting and collection of serious adverse events

The principal investigator or a collaborator shall report all serious adverse events (as defined below), whether or not considered treatment-related or expected, to the FV-UICEC-HUVR Pharmacovigilance department within 24 hours (one working day) of becoming aware of them (Annex XIII). Serious adverse events occurring at any time from the patient's inclusion in the study (which is defined as the time the subject signs the informed consent) and up to 90 days from the date of drawing the first positive blood culture or leaving the study must be reported. A subject is considered complete EITHER after the conclusion of the last visit or contact (e.g., telephone contact with the investigator or a collaborator), as indicated in the protocol evaluation schedule, OR after the last dose of study medication, whichever is later. Withdrawal is defined as the date on which a subject and/or the investigator determines that the subject can no longer meet the requirements of the trial at any subsequent visits and assessments.

The investigator shall complete and sign the AAG notification form (Annex XIII) and send it by fax or e-mail to:

Clinical Research and Clinical Trials Unit
Virgen del Rocío University Hospital
Pharmacovigilance Dept.
email: pv_shorten2@scren.es
Avda. Manuel Siurot S/N
41013. Seville
Tel.: 955 01 34 14
Fax: 955095338

The PV staff shall review the form received and, if appropriate, request additional information from the investigator. The investigator will provide information to the sponsor or whoever assumes the tasks delegated by the sponsor (FV-UICEC-HUVR Unit) whenever requested to do so and in any case when their initial assessment of severity or causality changes. The reporting procedure described above will be followed for communicating follow-up information.

FV-UICEC-HUVR staff will keep a detailed record of all AAGs or of special interest that are reported to them by researchers.

In the event of a medication error or use of the investigational medicinal product outside the protocol during the conduct of the trial, the investigator shall notify the FV-UICEC-HUVR within 24 hours of becoming aware of it. The reporting circuit and form shall be the same as for AAGs.

Clinically significant events that are not life-threatening or life-threatening or require hospitalization may be considered as serious adverse drug experiences when, based on sound medical judgement, they are likely to endanger the subject or require medical or surgical intervention to prevent one of the outcomes listed in this definition. Examples of such medical events are allergic bronchospasm requiring intensive, home or emergency department treatment, blood dyscrasias or seizures not resulting in hospitalization, or development of dependence or substance abuse.

Laboratory test abnormalities also need to be reported, unless otherwise stated in this section of the protocol.

9.6 Expedited notification of RAGI

The FV-UICEC-HUVR department is responsible for notifying the AEMPS and the Autonomous Regions where the trial is conducted of all RAGI collected in the study, following the procedure indicated in the legislation in force.

The maximum period for notification of an individual case of suspected RAGI shall be 15 calendar days from the time the sponsor becomes aware of the suspected RAGI. Where the suspected RAGI has resulted in the death or endangerment of the patient, the sponsor shall submit the information within 7 calendar days of becoming aware of the suspected RAGI. He/she shall complete the information, if possible, within 8 days.

This information should include an assessment of the significance and implication of the findings, including relevant previous experience with the same or similar medicinal products.

Likewise, the competent body of each of the Autonomous Communities where the trial is being conducted should be notified of suspected ISAGR occurring in the healthcare centres of their Community. In both cases, the RAGI notification form should be used for this purpose.

9.7 Expedited notification of other relevant safety information

The FV-UICEC-HUVR department should report as soon as possible and no later than 15 days after it becomes aware of any information that could change the benefit/risk ratio of the investigational product (e.g. increase in the rate of occurrence of expected AGRs, AGIRs occurring after completion of a clinical trial, new events related to the conduct of the trial or development of the investigational product, any recommendations of the data monitoring committee relevant to subject safety, etc.).

9.8 Notification to investigators

The sponsor will submit safety information that could impact the safety of patients enrolled in the study to the investigators as soon as possible.

In addition, the investigator will be informed throughout the study of any safety issues, including modifications to the protocol due to safety concerns.

9.9 Medication errors

Medication errors are unintentional mistakes in prescribing, dispensing, administering or monitoring a medicine while under the control of a healthcare professional or patient that may cause harm to the patient.

Misuse refers to a situation where the medical product is intentionally used inappropriately, not in accordance with protocol.

Study medication errors and off-protocol use are recorded in the data collection notebook (CRD), regardless of whether they are associated with an AA/AAG or not. Misuse or abuse will be collected and reported in the safety database within 24 hours of the investigator's knowledge of the misuse or abuse.

10.- STATISTICS

10.1 Sample size calculation

Definition of superiority. Superiority of the experimental treatment for the primary outcome variable of the study is established if the 7-day treatment is ≥60% better than the 14-day treatment, as assessed by the DOOR/RADAR scale.

To calculate the sample size, the Mann-Whitney U-test was performed for one null hypothesis of a 60% probability that X > Y (where "X" is the DOOR/RADAR score of a randomly selected patient from the experimental group, and "Y" is the DOOR/RADAR score of a randomly selected patient from the control group), following the previously published methodology.

To confirm this hypothesis of superiority, the estimated sample size required will be of 262 patients, assuming an error $\alpha = 0.05$, a power of 80%.

The hypothesis of non-inferiority shall be assessed for the treatment failure variable evaluated. at 30 days after discontinuation of trial treatment. An observational study published by Bae et al. (21), reports an all-cause recurrence or death rate at this point of 15.6% and 11.3% for patients receiving prolonged or short treatments. Considering this result and choosing a non-inferiority margin of 7.5%, a power of 80%, a one-sided α-error of 0.025, the estimated sample size would be 262.

The final sample number has been adjusted, expecting a non-adherence to the treatment of 5% and a loss to follow-up of 5%, so that the estimated sample size needed to test the non-inferiority hypothesis will be 306 patients. This estimates sufficient power for both variables.

10.2 Statistical analysis

The primary analysis will be performed in the intention-to-treat (ITT) population (all randomized patients) and in the per-protocol (PP) population (all patients who comply with the study protocol without major deviations).

The statistical analyses to be carried out will be as follows:

1. Descriptive study of all variables. Continuous variables to be reported as median and interquartile range and categorical variables as absolute numbers and percentages.

2. Chi-square test or Fisher's exact test to compare qualitative variables in the two study groups.

3. Student's t-test or Mann-Whitney U test for quantitative variables, according to their fit to the normal distribution.

4. Multivariate analysis using logistic regression or linear regression as appropriate for associated variables independently of outcome variables.

5. A pre-defined subgroup analysis will be performed for bacteraemias of pulmonary and extrapulmonary origin.
6. Missing data will be analyzed by multiple imputation.
7. DOOR/RADAR analysis: in addition, an analysis will be performed using the desirability of outcome ranking (DOOR) and response adjusted for duration of antibiotic risk (RADAR) with the classification indicated in section 5.1.1.

All analyses will be carried out with the statistical software R version 3.6.3 and SPSS version 22.0.

Special considerations: an expert committee (section 10.6) to assess patients with treatment failure, specifically proven or probable recurrence, as the non-objective outcome variable. Due to the above criteria they will be subject to final assessment by a panel of study investigators. This committee will be blinded so as to be unaware of the assignment of the patient being evaluated to the experimental or control group. The three investigators on the committee will be asked to confirm or not the recurrence of bacteraemia, in this case providing an alternative explanation. In case of discrepancy, the final decision shall be taken by majority vote.

10.3 Analysis populations

Analyses of primary and secondary outcomes will be conducted on intention-to-treat (ITT) and per-protocol (PP) populations. These are defined as follows:

- Intention-to-treat population: All randomized patients constitute this population, whether or not patients continued to receive antibiotic therapy.
- Per-protocol population: This group consists of all patients who were randomized, received the assigned duration of antibiotic therapy (within ± 24 hours), had a follow-up at day +30 from discontinuation of antibiotic treatment and in whom no major protocol deviations were documented during the study period.

10.4 Security analysis and other secondary results

The frequency and degree of severity of AEs considered to be possibly, probably or definitely related to the study antibiotic shall be described by organ system class and by the preferred Medical Dictionary for Regulatory Activities (MedDRA) term. The frequency of AEs shall be reported and compared between intervention groups using Fisher's exact test or the Chi-square test, as appropriate.

10.5 Intermediate analysis

An interim analysis will be performed when 40% of the sample is recruited and all results defined for the final analysis will be evaluated.

This will be conducted by an independent evaluation committee that will have full access to all study data (see section 2.6.1). The potential futility of the trial will be assessed by means of the conditional power (CP) for the secondary endpoint based on the trend observed in the interim analysis, as well as that projected if the initial hypothesis is met. A CP <15% is considered unlikely to demonstrate treatment efficacy, and therefore trial discontinuation will be jointly assessed if this margin is crossed. In addition, any differences in mortality between groups will be carefully evaluated.

These data will be evaluated by an independent data and safety monitoring board composed of 3 expert investigators belonging to the CIBER of infectious diseases and not participating in this study (DSMB). The composition of these members will be communicated to the Ethics Committee prior to the start of the trial.

The main responsibilities of the DSMB are

1) Periodically review and evaluate cumulative study data for participant safety, study conduct and progress, and where appropriate, efficacy.

2) Make recommendations to the study coordination team on the continuation, modification, or termination of the trial. For this purpose, a clear definition of the meeting schedule, stopping rules with statistical descriptions, and selected committee members will be approved prior to the start of the study.

10.6 Independent Evaluation Committee

To avoid as far as possible the bias that the open-label nature of the trial could generate, the evaluation of the results will be carried out by an independent blinded committee (not taking part in the study as investigators) with respect to treatment allocation. This committee will be formed by three expert investigators belonging to the CIBER of infectious diseases and will reach its conclusions by consensus.

11.- ETHICAL ASPECTS

The trial will be conducted in accordance with the principles emanating from the Declaration of Helsinki, and according to the legal regulations in force (Royal Decree 1090/2015), and will not be initiated until the approval of the CEIC of reference, the conformity of the directors of the Institutions, and the authorisation of the Spanish Agency of Medicines and Health Products has been obtained.

The investigator should comply with all requirements of the protocol. If a situation arises where a temporary deviation from the protocol is required, the investigator or other

physician responsible for the patient should contact the monitor as soon as possible to discuss the situation and agree on an appropriate course of action. The investigator shall document the deviation from the protocol and the circumstances that necessitated the deviation.

11.1 Informed consent

The patient must give consent before being admitted to the clinical trial. The physician should explain the nature, purpose and possible consequences of the clinical trial in a manner that is understandable to the patient. The information provided by the physician should also be recorded. In obtaining and documenting it, investigators shall comply with the relevant legislation (Article 4 of Royal Decree 1090/2015), the standards of good clinical practice and the ethical principles that originate from the Declaration of Helsinki.

The study subject will give consent by signing the appropriate consent form. The investigator will receive an appropriate number of informed consent forms from the sponsor. For this purpose, each form should be signed by the investigator and the patient.

The investigator shall not initiate any research pertaining to the trial until the consent of the patient has been obtained.

11.2 Data protection

The processing, communication and transfer of the personal data of all participating subjects shall comply with the provisions of Regulation (EU) 2016/679 of the European Parliament and of the Council of 27 April 2016 on the protection of natural persons with regard to the processing of personal data and the free movement of such data and Organic Law 3/2018 of 5 December on the Protection of Personal Data and guarantee of digital rights. In accordance with the provisions of the aforementioned legislation, patients may exercise their rights of access, modification, opposition and cancellation of data by contacting their study doctor.

The anonymity of the subjects participating in the study will be maintained at all times. Thus, the data collected for the study will be identified by a code and only the investigator and collaborators will be able to relate these data to the patient and his/her clinical history. Therefore, the patient's identity will not be revealed to any person, with the following exceptions: personnel authorised by the sponsor, when necessary, to check the study data and procedures, but always maintaining their confidentiality in accordance with current legislation; in the event of a medical emergency or legal requirement (health authorities: Spanish Agency for Medicines and Health Products and Local Clinical Trials Committee).

Data from this study will be used only for the specific purposes of the study.

11.3 Responsibilities of study participants

The subject should follow the instructions of the investigators and report any eventuality to the investigators. The subject shall be informed of any prohibitions or restrictions to which he/she must adhere during the trial.

Failure to comply with these recommendations will result in the abandonment of the study.

All subjects participating in the study have the right to leave the study at any time by withdrawing their consent, without having to justify this decision and without any detriment to their clinical follow-up. If this occurs, the investigator will attempt to have the subject complete all necessary assessments to ensure that no adverse events occur and to ensure appropriate follow-up in the event of any problems.

11.4 Monitoring and auditing

The study will be monitored through local visits, phone calls and periodic inspection of the CRDs with sufficient frequency to check the following:

- Pace of patient inclusion, compliance with protocol procedure standards, completeness and accuracy of data entered in the logbooks, verification against original documents and occurrence of adverse events.
- Monitoring visits will be conducted by the study monitors. It is understood that these monitors will have access to patient records upon request by the investigator. The investigator will devote sufficient time to these visits and will provide access to all documentation to authorised persons.
- The study may be audited by an independent body. The study may also be monitored by members of the ECCI.

11.5 Premature termination or suspension of the study

If the trial is prematurely terminated or suspended, the sponsor should promptly inform the investigator and the regulatory authority(ies) of the termination or suspension and the reason for it. The sponsor or investigator should promptly inform the IRB/IEC and provide the reason for the termination or suspension as specified in the relevant regulatory requirements.

11.6 Documentation of the study

The documentation related to the study (protocol, CRD, informed consent, authorizations...) will be archived in a safe and easily accessible place by the research team. All information contained in clinical, histological, biochemical reports, observations or other activities is necessary for the reconstruction and evaluation of the study.

12.- FINANCING AND INSURANCE

12.1 Financing

The project has received funding through a public call for independent clinical research from the Instituto de Salud Carlos III with file number ICI21/00075.

12.2 Insurance

An application will be made to the ethics committee for consideration as a low-intervention clinical trial in accordance with the definition established in RD 1090/2015. In the event of not being accepted, the sponsor will take out, in accordance with Spanish legislation, a civil liability insurance policy, in accordance with current legislation (RD 1090/2015, article 9). This policy will cover all possible damages that the subject may suffer as a result of the administration of the product under study. This policy shall be paid for and effective before the start of the clinical trial, if the trial is approved by the corresponding health authorities.

13.- PUBLICATION POLICY

These shall comply with the provisions of Royal Decree 1090/2015 of 4 December, which regulates Clinical Trials with medicinal products, the Ethics Committees for Research with medicinal products and the Spanish Register of Clinical Studies, article 42, which contains the following text:

"The sponsor is obliged to publish the results, both positive and negative, of authorized clinical trials, preferably in scientific journals before they are disclosed to the non-healthcare public, irrespective of the obligations to publish the report of the results in the Spanish Register of Clinical Studies (REec) and of the provisions of Regulation (EU) No 536/2014 of the European Parliament and of the Council of 16 April 2014 in this regard.

2. When studies and research work on medicinal products are made public to the scientific community, the funds obtained by the author, by or for their execution, and the source of financing shall be stated.

The anonymity of the subjects participating in the trial shall be maintained at all times.

4. Treatments of as yet undetermined efficacy shall not be prematurely or sensationally publicised or exaggerated. Intermediate results that may compromise the reliability of the final trial results shall not be publicised.

5. Advertising of investigational medicinal products for human use is strictly prohibited, as established in the revised text of the Law on Guarantees and Rational Use of Medicines and Medical Devices, in Royal Decree 1416/1994, of 25 June, which regulates the advertising of medicinal products for human use, in Royal Decree 1907/1996, of 2 August, on advertising and commercial promotion of products, activities or services intended for health purposes, and in Law 34/1988, of 11 November, General Law on Advertising.

6. In all cases, the guidelines of the European Commission and, where appropriate, the instructions of the Spanish Agency for Medicinal Products and Health Products shall be followed for making public the general results of the investigations once they have been completed.

7. Where a substudy of a clinical trial ends at a later date than the rest of the trial, the summary of the results of the trial shall be published within one year of the end of the trial, without delaying the reporting of the results of the rest of the trial.

ANNEX I. BIBLIOGRAPHY

WHO Global action plan on antimicrobial resistance. WHO. [cited 28 Jan. 2019]. http://apps.who.int/iris/bitstream/handle/10665/193736/9789241509763_eng.pdf?sequence=1

Annual Report 2019-2020 National Antibiotic Resistance Plan (NARP). [cited 17 March 2021]. https://resistenciaantibioticos.es/es/publicaciones/informe-anual-2019-2020-plan-nacional-frente-laresistencia-los-antibioticos.

3.European Centre for Disease Prevention and Control (ECDC). Antimicrobial resistance strategies and action plans. 2016 https://www.ecdc.europa.eu/en/publications-data/directory-guidance-prevention-and-control/antimicrobial-resistance-strategies

4. Rodríguez-Baño J, et al. Antimicrobial use optimisation programmes (PROA) in Spanish hospitals: consensus document GEIH-SEIMC, SEFH and SEMPSPH. Infectious Infectious Diseases Microbiol Clin. 2012;30(1):22.e1-22.e23.

5. Molina J, et al. Long-Term Impact of an Educational Antimicrobial Stewardship Program on Hospital-Acquired Candidemia and Multidrug-Resistant Bloodstream Infections: A Quasi-Experimental Study of Interrupted Time-Series Analysis. Clin Infect Dis. 2017; 65(12):1992-9.

6. Peñalva G, et al. Long-term impact of an educational antimicrobial stewardship programme in primary care on infections caused by extended-spectrum β-lactamase-producing Escherichia coli in the community: an interrupted time-series analysis. Lancet Infect Dis. 2020; 20(2):199-207.

7. Uranga A, et al. Duration of Antibiotic Treatment in Community-Acquired Pneumonia: A Multicenter Randomized Clinical Trial. JAMA Intern Med. 2016; 176(9):1257-65.

8. Chastre J, et al. Comparison of 8 vs 15 days of antibiotic therapy for ventilator-associated pneumonia in adults: a randomized trial. JAMA. 2003; 290(19):2588-98.

9. Eliakim-Raz N, et al. Duration of antibiotic treatment for acute pyelonephritis and septic urinary tract infection-- 7 days or less versus longer treatment: systematic review and meta-analysis of randomized controlled trials. J Antimicrob Chemother. 2013; 68(10):2183-91.

10. Sawyer RG, et al. Trial of Short-Course Antimicrobial Therapy for Intraabdominal Infection. N Engl J Med. 2015; 372(21):1996-2005.

11. Aguilar-Guisado M, et al. Optimisation of empirical antimicrobial therapy in patients with haematological malignancies and febrile neutropenia (How Long study): an open-label, randomised, controlled phase 4 trial. Lancet Haematol. 2017; 4(12):e573-83.

12. Yahav D, Franceschini E, Koppel F, Turjeman A, Babich T, Bitterman R, et al. Seven Versus 14 Days of Antibiotic Therapy for Uncomplicated Gram-negative Bacteremia: A Noninferiority Randomized Controlled Trial. Clin Infect Dis 2019; 69(7):1091-8.

13. von Dach E, et al. Effect of C-Reactive Protein-Guided Antibiotic Treatment Duration, 7-Day Treatment, or 14-Day Treatment on 30-Day Clinical Failure Rate in Patients With

Uncomplicated Gram-Negative Bacteremia: A Randomized Clinical Trial. JAMA. 2020; 323(21):2160-9.
14. Araos R and D'Agata E. Mandell, Douglas and Bennett's Principles and Practice of Infectious Diseases. Elsevier; 2020. p. 2686-99.
15. Peña C, et al. Influence of virulence genotype and resistance profile in the mortality of Pseudomonas aeruginosa bloodstream infections. Clin Infect Dis. 2015; 60(4):539-48.
16. Recio R, et al. Pathogenic characteristics of Pseudomonas aeruginosa bacteraemia isolates in a high-endemicity setting for ST175 and ST235 high-risk clones. Eur J Clin Microbiol Infect Dis Off Publ Eur Soc Clin Microbiol. 2020; 39(4):671-8.
17. Sánchez-Diener I, et al. Weighting the impact of virulence on the outcome of Pseudomonas aeruginosa bloodstream infections. Clin Microbiol Infect. 2020; 26(3):351-7.
18. Mermel LA, et al. Clinical practice guidelines for the diagnosis and management of intravascular catheter-related infection: 2009 Update by the Infectious Diseases Society of America. Clin Infect Dis. 2009; 49(1):1-45.
19. Capdevila JA, et al. 2016 Expert consensus document on prevention, diagnosis and treatment of short-term peripheral venous catheter-related infections in adult. Rev Esp Quimioter. 2016; 29(4):230-8.
20. Bae M, Jeong Y, Bae S, Kim MJ, Chong YP, Kim S-H, et al. Short versus prolonged courses of antimicrobial therapy for patients with uncomplicated Pseudomonas aeruginosa bloodstream infection: a retrospective study. J Antimicrob Chemother. September 30, 2021;dkab358.
21. McCarthy KL, Paterson DL. Increased risk of death with recurrent
Pseudomonas aeruginosa bacteremia. Diagn Microbiol Infect Dis. 2017; 88(2):152-7.
22. Molina J, Cisneros JM. Editorial Commentary: A Chance to Change the Paradigm of Outcome Assessment of Antimicrobial Stewardship Programs. Clin Infect Dis. 2015; 61(5):807-8.
23. Evans SR, et al. Desirability of Outcome Ranking (DOOR) and Response Adjusted for Duration of Antibiotic Risk (RADAR). Clin Infect Dis. 2015; 61(5):800-6.
24. Evans SR, et al. Methodologies for pragmatic and efficient assessment of benefits and harms: Application to the SOCRATES trial. Clin Trials 2020; 17(6):617-26.
25. Cabellos C, et al. Invasive meningococcal disease: Impact of short course therapy. A DOOR/RADAR study. J Infect. 2017; 75(5):420-5.
Celestin AR, et al. Novel Method Suggests Global Superiority of Short-Duration Antibiotics for Intra-abdominal Infections. Clin Infect Dis. 2017; 65(9):1577-9.
27. Schweitzer VA, et al. Response Adjusted for Days of Antibiotic Risk (RADAR): evaluation of a novel method to compare strategies to optimize antibiotic use. Clin Microbiol Infect. 2017; 23(12):980-5.
28. Ianiro G, et al. Antibiotics as deep modulators of gut microbiota: between good and evil. Gut. 2016; 65(11):1906-15.
29. Mu C, Zhu W. Antibiotic effects on gut microbiota, metabolism, and beyond. Appl Microbiol Biotechnol. 2019; 103(23-24):9277-85.

30. Elvers KT, et al. Antibiotic-induced changes in the human gut microbiota for the most commonly prescribed antibiotics in primary care in the UK: a systematic review. BMJ Open. 2020; 10(9):e035677.
31. Gori S, et al. Gut microbiota and cancer: How gut microbiota modulates activity, efficacy and toxicity of antitumoral therapy. Crit Rev Oncol Hematol. 2019; 143:139-47.
32. Taur Y, et al. The effects of intestinal tract bacterial diversity on mortality following allogeneic haematopoietic stem cell transplantation. Blood. 2014; 124(7):1174-82.
33. Korn EL, Freidlin B. Interim monitoring for non-inferiority trials: minimizing patient exposure to inferior therapies. Ann Oncol. 2018; 29(3):573-7.
34. EUCAST: Clinical breakpoints and dosing of antibiotics [cited 17 March 2021]. https://www.eucast.org/clinical_breakpoints/
35. Magiorakos AP, Srinivasan A, Carey RB, et al. Multidrug-resistant, extensively drug-resistant and pandrug-resistant bacteria: an international expert proposal for interim standard definitions for acquired resistance. Clin Microbiol Infect. 2012;18(3):268-281.

ANNEX II: LIST OF PARTICIPATING CENTRES

CENTRE	PRINCIPAL INVESTIGATOR
Virgen del Rocío University Hospital	Dr. María del Rocío Álvarez Marín
Virgen Macarena University Hospital	Dr. Luis Eduardo López Cortés
Virgen de Valme University Hospital	Dr. Nicolás Merchante Gutiérrez
Reina Sofia University Hospital	Dr. Juan José Castón Osorio
Virgen de las Nieves University Hospital	Dr. Miguel Ángel López Zúñiga
San Cecilio University Hospital	Dr. Francisco Anguita Santos
Torrecárdenas University Hospital	Dr. María Ángeles Esteban Moreno
Regional University Hospital of Malaga	Dr. Juan Diego Ruiz Mesa
Costa del Sol Hospital	Dr. Alfonso del Arco Jiménez
Virgen de la Victoria University Hospital	Dr. Rosario Palacios Muñoz
Juan Ramón Jiménez University Hospital	Dr. María Franco Huerta
Jerez de la Frontera University Hospital	Dr. Antonio Jesús Hidalgo Castellón
Puerto Real University Hospital	Dr. Patricia Jiménez Aguilar
Jaén Hospital Complex	Dr. Carmen Herrero Rodríguez
Ramón y Cajal University Hospital	Dr. Pilar Martín Dávila
La Paz University Hospital	Dr. María Belén Loeches Yagüe
Cruces University Hospital	Dr. Ane Josune Goikoetxea Agirre
Donostia University Hospital	Dr. Maialen Ibarguren Pinilla
San Pedro University Hospital	Dr. José Antonio Oteo Revuelta
Marqués de Valdecilla University Hospital	Dr. M. Carmen Fariñas Álvarez
Central University Hospital of Asturias	Dr. Emilio García Prieto
Vigo University Hospital Complex	Dr. Adrián Sousa Domínguez
A Coruña University Hospital Complex	Dr. Mª Dolores Sousa Regueiro
Lucus Augusti University Hospital	Dr. Blanca Ayuso García

Lozano Blesa Clinical University Hospital	Dr. José Ramón Paño Pardo
Bellvitge University Hospital	Dr. Mireia Puig Asensio
Parc Taulí Hospital	Dr. Aina Gomila Grange
Vall d'Hebron University Hospital	Dr. Isabel Ruiz Camps
Son Espases University Hospital	Dr. Helem Vílchez Rueda
La Fe University Hospital	Dr. Paula Ramírez Galleymore

ANNEX III. FLOWCHART

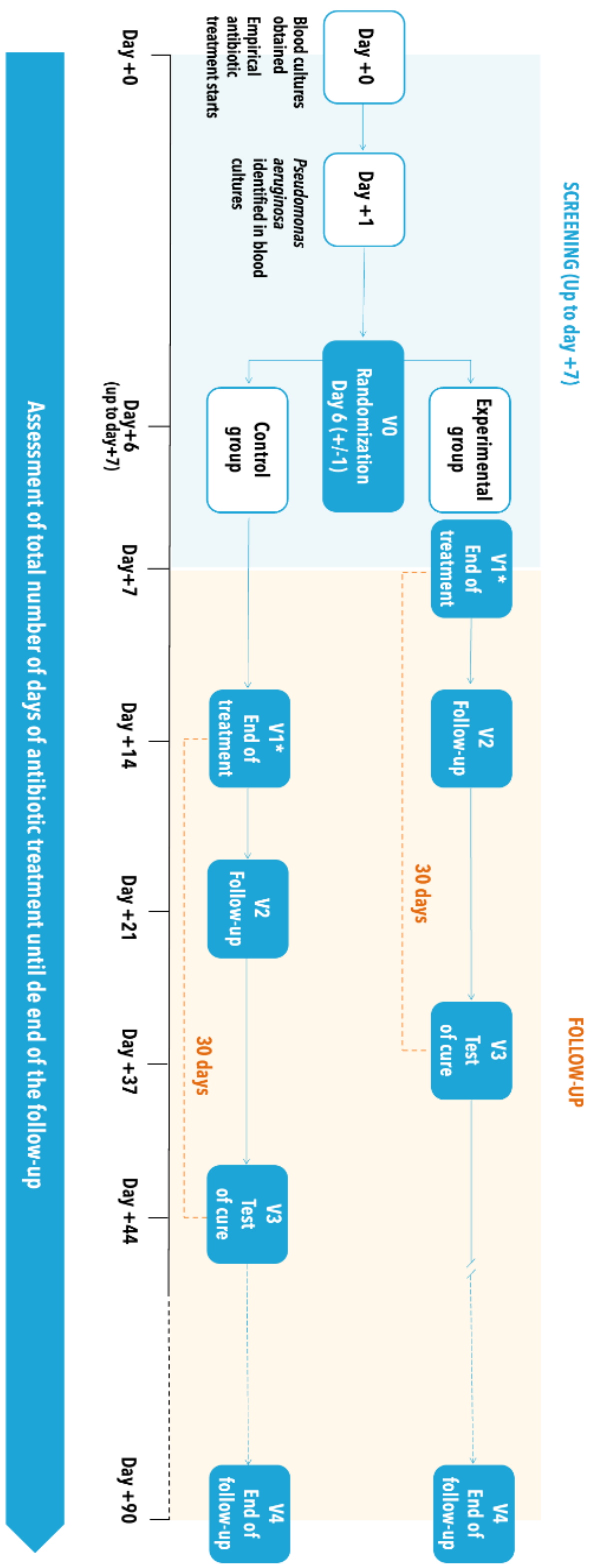

* Antibiotic treatment will be stopped at this point if the patient remain apyretic and without symptoms of infection for at least 72 hours. Antibiotic treatment could be resumed after this point whenever considered necessary by the clinician in charge of the patient if an unfavourable course was observed.

ANNEX IV. EUCAST

https://www.eucast.org/fileadmin/src/media/PDFs/EUCAST_files/Breakpoint_tables/v_11.0_Breakpoint_Tables.pdf

ANNEX V. DIDACTIC MATERIAL

A QUICK GUIDE FOR OPTIMISING YOUR ANTIBIOTIC TREATMENTS FOR BLOODSTREAM INFECTIONS PRODUCED BY *Pseudomonas aeruginosa*.

1 WHICH AGENT?

- The letter "I" in antibiograms of *P. aeruginosa* does not mean "intermediate susceptibility" anymore; now it means **"increase antibiotic exposure"**. This change affects only to some antibiotics.
- With the proper dose and administration, **you can use any option in the antibiogram, as long as they are not categorized as "R"** (resistant).

	MIC breakpoint to consider a treatment as active (mg/L)		MIC breakpoint to consider a treatment as active (mg/L)
Ciprofloxacin	≤0,5	Imipenem	≤4
Levofloxacin	≤1	Meropenem	≤8
Piperacillin/tazobactam	≤16	Meropenem-vaborbactam	≤8
Ceftazidime	≤8	Colistin	≤2
Cefepime	≤8	Amikacin	≤16
Ceftazidime/avibactam	≤8	Tobramicin	≤2
Ceftolozane/tazobactam	≤4	Aztreonam	≤16

- **Avoid agents with a MIC value exactly on the breakpoint concentration** (e.g. ceftazidime = 8 mg/L, piperacillin/tazobactam = 16 mg/L, etc.) **in severe infections or complicated sources.** The risk of treatment failure in these cases could be higher.

2 WHICH DOSE?

- Infections produced by *P. aeruginosa* require **higher doses.**

Agents currently approved by AEMPS	Anti-pseudomonic doses	
**Fluorquinolones**	**Oral**	**Intravenous**
**Ciprofloxacin**	750 mg q12h	400 mg q8h
**Levofloxacin**	500 mg q12h	500 mg q12h
**Piperacillin/tazobactam**	4 g / 0.5 g q8h administered in 4h-extended infusion.<br>4 g / 0.5 g q6h in 4h-extended infusion in severe infections produced by strains resistant to ceftazdime or pneumonia.	
**Ceftazidime, cefepime**	2 g q8h	
**Ceftazidime/avibactam**	2 g q8h administered in 2 h	
**Ceftolozane/tazobactam**	1 g / 0.5 g q8h<br>In case of pneumonia, recommended dose is 2 g / 1 g q8h	
**Imipenem**	1 g q6h	
**Meropenem**	**MIC ≤2 mg/L**: 1 g q8h<br>**MIC >2 mg/L and ≤8 mg/L**: 2 g q8h administered in 3 h	
**Meropenem-vaborbactam**	2 g / 2 g q8h in 3h-extended infusion	
**Colistin**	4,5 MU q12h	
**Amikacin**	25-30 mg/kg q24h	
**Tobramycin**	6-7 mg/kg q24h	
**Aztreonam**	2 g q6h	

3 WHICH WAY OF ADMINISTRATION?

- If you are going to use betalactams, always **administer them with an extended or continuous infusion.** This has proved to reduce mortality among patients with severe infections produced by *P. aeruginosa*.

4 MONOTHERAPY OR COMBINATION?

- **If a fully-active agent is available, use it as a monotherapy.** Combination therapy has not proved improved outcomes, but an increased risk of toxicity.

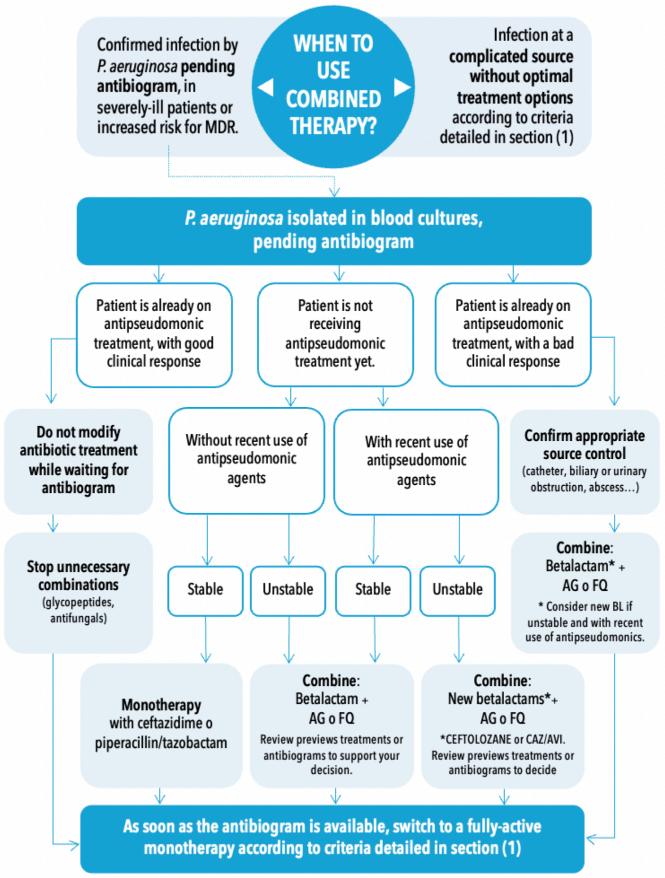

In the case of infections at complicated sources produced by strains without optimal treatment options according to these criteria, it may be recommendable to cotinue combination therapy

AG = Aminoglycoside (tobramycin or amikacin) **FQ** = Fluorquinolones **CAZ/AVI**: Ceftazidime/avibactam

5 CONTROL THE SOURCE

- Remove that catheter, drain that abscess… *P. aeruginosa* will learn fast, as it has a great ability to develop antibiotic resistance during treatments.

References

- EUCAST Breakpoint tables for interpretation of MICs and zone diameters, version 10.0, 2021.
- Prolonged versus short-term intravenous infusion of antipseudomonal β-lactams for patients with sepsis: a systematic review and meta-analysis of randomised trials. Vardakas et al. Lancet ID 2018.
- Impact of borderline minimum inhibitory concentration on the outcome of invasive infections caused by Enterobacteriaceae treated with β-lactams: a systematic review and meta-analysis. Torres et al. Eur J Clin Microbiol Infect Dis, 2015.
- Beta-lactam monotherapy or combination therapy for bloodstream infections or pneumonia due to P. aeruginosa: a meta-analysis. Onorato L *et al.* Int J Antimicrob Agents. Dec 2021;106512.

ANNEX VI. QUESTIONNAIRE FOR VISITS 3, 4 AND UNPLANNED VISITS

Visit 3 (day +30 from discontinuation of antibiotic treatment) and visit 4 (day +90 from the date of extraction of the first positive blood culture) can be carried out by telephone if the patient is not hospitalized. In order to facilitate the collection of essential data to complete the data collection notebook, the following structured questionnaire is proposed:

1. When were you discharged from hospital?

2. If you had not completed antibiotic treatment prior to discharge from hospital, have you continued to receive antibiotic treatment at home by intravenous or oral route? If yes:

   a) What treatment have you received?

   b) When did you start and when did you finish?

3. Since discontinuing antibiotic treatment in the trial:

   a) Have the symptoms of infection reappeared (refer to the local symptoms that were present at the beginning of the infectious episode for which you were included in the study)?

   b) Have you ever had a fever? If yes, has the fever been accompanied by any other signs or symptoms of infection? Please specify.

   c) Have you had samples taken on suspicion of possible infection (blood culture, urine culture, etc.)? If the answer is positive:

      - When was the sample taken?
      - What kind of sample?
      - Do you know the result of that cultivation?

   d) Have you been admitted to hospital? If the answer is yes:

      - What was the reason for this?
      - Could you please indicate the centre where you were admitted and the dates of admission and discharge?

   e) Have you required antibiotic treatment for any reason? If yes:

      - What was the reason for this?
      - What treatment has he/she received (antibiotic, dose, frequency and route of administration)?
      - When did the treatment start and end?

- Why did you stop treatment (switch to oral route, end of treatment, unfavourable evolution, superinfection, adverse effect or other)? Please specify.

f) Have you had any side effects related to the antibiotic treatment you received?

- Have you had diarrhoea (more than 3 bowel movements per day in patients with a previously normal bowel habit)?
- Have you noticed any rashes?
- Has your doctor detected any analytical abnormalities that he/she has linked to the previous antibiotic treatment?

ANNEX VII. ALGORITHM FOR ALLOCATING PATIENTS TO PROVEN, PROBABLE OR POSSIBLE RECURRENCE CATEGORIES.

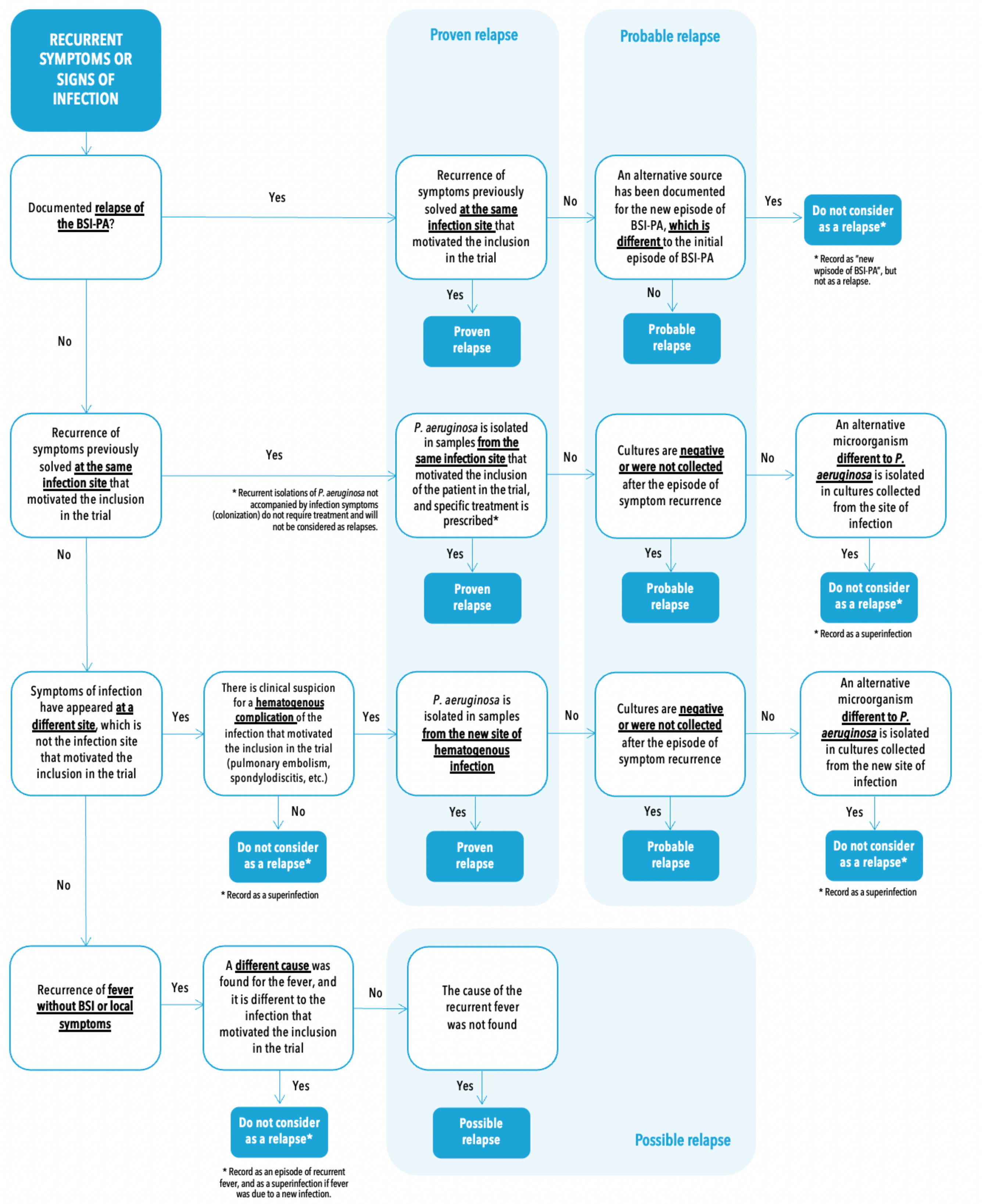

ANNEX VIII. PATIENT INFORMATION AND INFORMED CONSENT FORM.

It is attached in a separate document.

ANNEX IX. CHARLSON INDEX

SCORE	CONDITION
1	Myocardial infarction<br>Congestive heart failure<br>Peripheral vascular disease<br>Cerebrovascular disease<br>Dementia<br>Chronic lung disease<br>Connective tissue disease<br>Peptic ulcer<br>Mild liver disease<br>Diabetes without target organ involvement
2	Hemiplegia or paraplegia<br>Moderate or severe kidney disease<br>Diabetes with target organ involvement<br>Any tumour
3	Moderate or severe liver disease
6	Metastatic solid tumour<br>AIDS

Charlson ME, Pompei P, Ales KL, MacKenzie CR. A new method of classifying prognostic comorbidity in longitudinal studies: development and validation. J ChronDis 1987; 40: 373-383.

ANNEX X. PITT SCORE

Collect the highest score between the day of bacteraemia and the previous 48 hours.

CRITERIA		POINTS
Fever (Tª)	≤ 35ºC or ≥40ºC	2
	35,1-36 ó 39-39,9	1
	36,1-38,9	0
Hypotension	Acute decrease in systolic BP of 30 mmHg or diastolic BP of 20 mmHg	2
	intravenous vasopressor drug requirements	2
	Systolic BP < 90 mmHg	2
Mechanical ventilation		2
Heart failure		4
State of mind	Alert	0
	Disoriented	1
	Stupefying	2
	Coma	4

ANNEX XI. PROCEDURE FOR THE SUBMISSION OF MICROBIOLOGICAL ISOLATES AND FAECAL SAMPLES FOR GUT MICROBIOTA STUDIES.

Procedures for the submission of microbiological isolates:

1. Micro-organisms once identified shall be frozen at -80°C following the standard procedure of each centre. It shall be stored and remain uninterrupted until shipment. Any loss (even transient) of the preservation conditions of the samples shall be reported to the Study Coordinating Centre.

2. Immediately after collection, each specimen shall be uniquely identified and numbered with a label bearing the Facility Identification Code, the patient identification number of the patient in the study, the specimen identification number, and the date the specimen was collected.

3. Samples will remain at the participating Centre under the responsibility of the researchers of the Centre until they are sent. The transfer of all the samples from each centre will be carried out in successive shipments through a transport company (Enviplus Sl, Seville, Spain). The study coordination centre will specify and agree with each centre the day and specific details of sample collection and shipment.

4. A shipment to the reference centre must be prepared every 3 months. The transfer of all samples from each centre will be carried out in successive shipments through a transport company (Enviplus Sl, Seville, Spain). The study coordination centre will specify and agree with each centre the day and specific details of sample collection and shipment.

5. From the frozen strains, subculture on a blood agar plate. Incubate in an oven at 37°C for 18-20 hours until adequate bacterial growth is achieved. The growth on these plates shall serve as inoculum for transport.

6. Micro-organisms shall be swabbed in Amies, Stuart or Cary-Blair transport medium at room temperature. For this purpose a sterile swab shall be used to collect an abundant amount of micro-organisms from the agar surface of the subcultured plate. The swab shall be placed in the tube containing the transport medium. Fit the cap on the tube ensuring a good seal. **Identify the isolate clearly on the label of each tube using the reference number of the sample from the sending centre.**

7. Each isolate must be correctly identified and accompanied by the corresponding isolation form (Annex XII) and a copy of the antibiogram report from the sending centre.

8. Samples shall be sent to:

A/A Mª Eugenia Pachón Ibáñez
Institute of Biomedicine of Seville (IBiS)
Laboratory 208, first floor
C/ Antonio Maura Montaner, S/N
41013, Seville.
Telephone: 955923100 7 650720131
mpachon-ibis@us.es

9. The samples must be packaged and transported as category A infectious substances, in accordance with the regulations in force by the WHO (Guide on the regulation of the transport of infectious substances 2011-2012) and BOE no. 63 (14/3/2013).

10. Once collected by the courier, contact the reference centre by e-mail (mpachon-ibis@us.es) indicating in the subject "SHORTEN II SHIPMENT_Name of the sending hospital" to confirm the shipment of samples indicating in the text of the e-mail:

a. Name of hospital

b. Date of departure of the consignment

c. Name and telephone number of contact person making the shipment

d. Number of micro-organisms shipped

e. Confirmation that the appropriate form for each strain has been included in the shipment.

11. Once the samples have been registered, all *P. aeruginosa* isolates that meet the criteria for recurrent bacteraemia or are to be used as controls for clonal identity studies will be sent. An agreement will be reached with Mercedes Delgado of the Microbiology Department of the Hospital Universitario Virgen Macarena on the day and specific details for sending the samples.

Faecal samples for microbiota analysis

1. *Stool Nucleic Acid Collection and Preservation Tubes* [Cat. 45660, Norgen Biotek Corp.] will be shipped from the reference centre (IBiS) to the different participating centres.

2. Once agreed between the participating centres and IBiS, the samples will be collected. These tubes allow samples to be stored and shipped at room temperature; depending on specifications, they can also be stored at -20ºC or -70ºC.

Procedure for sample collection:

- Place the "wedge" together with a soaker on the toilet.
- Remove a small sample from 3 different sites using the teaspoon from the jar.
- Do not exceed the level set by the boat.

- Tighten the cap of the bottle so that everything is mixed together when shaken gently.

3. Immediately after collection of each sample:

- It shall be uniquely identified with a label bearing the Facility Identification Code, the patient's study identification number, the sample identification number, and the date the sample was collected.
- Correctly identified, it shall be stored and remain uninterrupted until shipment. Any loss (even transient) of the preservation conditions of the samples shall be reported to the Study Coordinating Centre. Until shipment, samples will remain at the participating site under the responsibility of the participating site investigators.

4. The transfer of all samples from each Centre will be carried out in successive shipments by a transport company (Enviplus Sl, Seville, Spain). The day and specific details of collection and shipment of the samples will be specified and agreed with each centre.

Samples will be sent for sequencing of the 16S rRNA V3-V4 hypervariable regions, using the Illumina MiSeq platform, and subsequent bioinformatic analyses to study the bacterial diversity in the gut microbiota of samples from both study groups to the following address:
:

A/A Mª Eugenia Pachón Ibáñez
Institute of Biomedicine of Seville (IBiS)
Laboratory 208, first floor
C/ Antonio Maura Montaner, S/N
41013, Seville.
Telephone: 955923100 7 650720131
mpachon-ibis@us.es

ANNEX XII: ISOLATION FORM

REFERENCE NUMBER<br>*(to be completed by reference centre)*	
NAME OF SENDING CENTRE	
DATE OF ISOLATION	
REFERENCE NUMBER OF THE SAMPLE AT THE SENDING SITE	
SERVICE OR UNIT OF ORIGIN	
METHOD OF IDENTIFICATION AT SENDING CENTRE	
ANTIBIOGRAM METHOD AT THE SENDING SITE	
ANTIMICROBIAL SUSCEPTIBILITY DATA<br>(it is recommended that they attach a copy of the report issued at the centre of origin).	

ANNEX XIII. AAG NOTIFICATION FORM

Subject code: ________________	TYPE OF REPORT: ☐ Initial ☐ Follow-up

1. CENTRE INFORMATION

No. of centre:	Principal Investigator:
Person notifying the AAG:	Tel:
Fax:	Mail:

2. SUBJECT INFORMATION

Sex	Age (At the beginning of the AA)	Age group (fill in only if the age of the subject is unknown)	Date of birth (dd-mmm-yyyy)	Weight (kg)	Height (cm)
☐ Male<br>☐ Female	_________<br>☐ Years ☐ Months ☐ Days	☐ Neonate ☐ Infant<br>☐ Child ☐Adolescent<br>☐ Adult ☐ Elderly	_ _ - _ _ _ - _ _ _ _		

3. ADVERSE EVENT.

Serious adverse event (Specify diagnosis or syndrome, if known. If unknown, include signs and symptoms)**:**

....

....

DESCRIPTION OF THE ADVERSE EVENT (Provide all information about the circumstances, sequence, diagnosis and treatment of the Adverse Event)

Start date (dd-mmm-yyyy):_ _ - _ _ _ _ _ - _ _ _ _ _ _ End date : (dd-mmm-yyyy):_ _ _ - _ _ _ _ _ - _ _ _ _ _ _ _ _ _ _

Severity criterion:	Unlinked (status at the time of notification):
☐ Death<br>☐ Life-threatening<br>☐ Requires or prolongs hospitalisation<br>☐ Permanent or significant disability<br>☐ Birth defect or congenital anomaly<br>Clinically relevant	☐ Recovered<br>☐In recovery<br>☐ Not recovered<br>Recovered with sequelae<br>☐Mortal<br>☐ Unknown

In the event of death, please complete the following information:

Date of exitus: _ _ _ - _ _ _ _ _ _ _ - _ _ _ _ _ _ _ _ _ Causa:____

Autopsy was carried out: ☐ Yes ☐ No ☐ Unknown

Indicate the cause of death according to the autopsy:

____

4. PRODUCT/INVESTIGATION MEDICATION (Indicate the treatment(s) assigned to the subject after randomization)
If you need more space, use copies of this page and tick this box ☐.

Medicines	Dosage and units	Frequency	Via	Start date (dd-mmm-yyyy)	End date (dd-mmm-yyyy) (if continued tick the box)	Causal relationship
					☐	☐ Related Unrelated
					☐	☐ Related Unrelated
					☐	☐ Related Unrelated

Action taken with medication in response to AA	☐ Withdrawal of medication ☐Temporary cessation of medication ☐ Unknown ☐ Dose reduction ☐ Dose increase ☐ No change Not applicable
Did AA recur when stopping medication or reducing dosage?	☐ Yes ☐ No ☐ Unknown ☐ Not applicable
Did AA reappear on reintroduction of medication?	☐ Yes ☐ No ☐ Unknown ☐ Not applicable

5. CONCOMITING MEDICATION (Include those concomitant and background treatments taken in the two weeks prior to the AA start date. Do not include treatment given to treat AA or treatment given after the date of AA onset.)
If you need more space, use copies of this page and tick this box☐.

Medicines	Daily dose (units)	Frequency	Via	Start date (dd-mmm-yyyy)	End date (dd-mmm-yyyy) (if continued tick the box)	Causal relationship	Indication
					☐	☐ Related Unrelated	
					☐	☐ Related Unrelated	
					☐	☐ Related Unrelated	

Action taken with medication in response to AA	☐ Withdrawal of medication ☐Temporary cessation of medication ☐ Unknown ☐ Dose reduction ☐ Dose increase ☐ No change Not applicable
Did AA recur when stopping medication or reducing dosage?	☐ Yes ☐ No ☐ Unknown ☐ Not applicable
Did AA reappear on reintroduction of medication?	☐ Yes ☐ No ☐ Unknown ☐ Not applicable

6. ALTERNATIVE CAUSE

Is there any possibility that the AA is related to an alternative cause to medication?
☐ Yes ☐ No If yes, please specify (further information in the AAG description section if necessary):

____

____

7. RELEVANT MEDICAL HISTORY

If you need more space, use copies of this page and tick this box☐.

Pathological history	Start date (dd-mmm-yyyy)	End date (dd-mmm-yyyy)	(if continued tick the box)
			☐
			☐
			☐
			☐

Supplementary information to the Medical History data

8. LABORATORY DATA AND OTHER SUPPLEMENTARY EXPLORATIONS: (indicate only test results relevant to documenting the reported MAA)

If you need more space, use copies of this page and tick this box☐.

TESTING PERFORMED	TEST DATE (dd-mmm-yyyy)	RESULT (Units)	Range Reference	COMMENT

Supplementary information on laboratory data and other examinations:

Signature of the notifying investigator	Date of notification
**Signature of the Pharmacovigilance Manager**	**Date of receipt of the notification**

SEND IMMEDIATELY BY FAX TO THE PHARMACOVIGILANCE NODE OF THE UICEC OF THE HOSPITAL UNIVERSITARIO VIRGEN DEL ROCÍO

(FAX: 955095338)

email: pv_shorten2@scren.es

ANNEX XIV. HELSINKI DECLARATION OF THE WORLD MEDICAL ASSOCIATION

Ethical principles for medical research involving human subjects
Adopted by the 18th World Medical Assembly, Helsinki, Finland, June 1964, and amended by the
29th World Medical Assembly, Tokyo, Japan, October 1975
35th World Medical Assembly, Venice, Italy, October 1983
41st World Medical Assembly, Hong Kong, September 1989
48th General Assembly Somerset West, South Africa, October 1996
52nd General Assembly, Edinburgh, Scotland, October 2000
Clarification Note to Paragraph 29, added by the WMA General Assembly, Washington 2002.
Clarification Note to Paragraph 30, added by the WMA General Assembly, Tokyo 2004.
59th General Assembly, Seoul, Korea, October 2008
64th General Assembly, Fortaleza, Brazil, October 2013

A. INTRODUCTION

1. The World Medical Association (WMA) has promulgated the Declaration of Helsinki as a set of proposed ethical principles for medical research involving human subjects, including research on identifiable human material and information.
The Declaration should be considered as a whole and one paragraph should be applied with consideration of all other relevant paragraphs.
2. In accordance with the WMA's mandate, the Declaration is intended primarily for physicians. The WMA urges others involved in medical research involving human subjects to adopt these principles.

B. GENERAL PRINCIPLES

3. The Declaration of Geneva of the World Medical Association binds the physician to the formula "the health of my patient first and foremost", and the International Code of Medical Ethics states that: "A physician shall consider the best interests of the patient when providing medical care".
4. It is the duty of physicians to promote and safeguard the health, welfare and rights of patients, including those involved in medical research. The physician's knowledge and conscience must be subordinate to the fulfilment of this duty.
5. Progress in medicine is based on research, which ultimately must include human studies.
6. The primary purpose of medical research in human subjects is to understand the causes, course and effects of diseases and to improve preventive, diagnostic and therapeutic interventions (methods, procedures and treatments). Even the best proven interventions

must be continuously evaluated through research to ensure their safety, efficacy, effectiveness, accessibility and quality.

7. Medical research is subject to ethical standards that serve to promote and ensure respect for all human beings and to protect their health and individual rights.

8. Although the primary objective of medical research is to generate new knowledge, this objective must never take precedence over the rights and interests of the research subject.

9. In medical research, it is the duty of physicians to protect the life, health, dignity, integrity, right to self-determination, privacy and confidentiality of personal information of research subjects. The responsibility for the protection of research subjects must always rest with a physician or other health care professional and never with the research participants, even if they have given their consent.

10. Physicians should consider the ethical, legal and regulatory norms and standards for research involving human subjects in their own countries, as well as existing international norms and standards. No national or international ethical, legal or regulatory requirement should be permitted to diminish or eliminate any of the protections for research subjects set forth in this Declaration.

11. Medical research should be conducted in a manner that minimizes potential harm to the environment.

12. Medical research involving human subjects should be conducted only by persons with appropriate scientific and ethical education, training and qualifications. Research on healthy patients or volunteers requires the supervision of a physician or other appropriately qualified and competent health care professional.

13. Groups that are under-represented in medical research must have appropriate access to participation in research.

14. A physician who combines medical research with medical care should involve his or her patients in research only to the extent that there is justified potential preventive, diagnostic or therapeutic value and if the physician has good reason to believe that participation in the study will not adversely affect the health of the patients taking part in the research.

15. Appropriate compensation and treatment should be ensured for individuals who are harmed during their participation in research.

C. RISKS, COSTS AND BENEFITS

16. In the practice of medicine and medical research, most interventions involve some risks and costs.

Medical research involving human subjects should only be undertaken when the importance of the research objective outweighs the risk and costs to the research subject.

17. All medical research involving human subjects must be preceded by a careful comparison of the risks and costs to the individuals and groups participating in the research compared with the foreseeable benefits to them and to other individuals or groups affected by the condition under investigation.

Measures should be implemented to minimize risks. Risks should be continuously monitored, assessed and documented by the investigator.
18. Physicians should not engage in research studies involving human subjects unless they are confident that the risks have been adequately assessed and can be satisfactorily managed.
When the risks involved outweigh the expected benefits or if there is conclusive evidence of definitive results, physicians should evaluate whether to continue, modify or discontinue the study immediately.

D. VULNERABLE GROUPS AND INDIVIDUALS
19. Some groups and individuals subject to the research are particularly vulnerable and may be more likely to suffer abuse or additional harm.
All groups and individuals should receive specific protection.
20. Medical research on a vulnerable group is only justified if the research responds to the health needs or priorities of this group and the research cannot be conducted on a non-vulnerable group. In addition, this group will be able to benefit from the knowledge, practices or interventions resulting from the research.

E. SCIENTIFIC REQUIREMENTS AND RESEARCH PROTOCOLS
21. Medical research involving human subjects must conform to generally accepted scientific principles and must be supported by a thorough knowledge of the scientific literature, other relevant sources of information, as well as by properly conducted laboratory and animal experiments, where appropriate. The welfare of animals used in experiments should also be taken into account.
22. The design and method of any human study should be clearly described and justified in a research protocol.
The protocol should always make reference to relevant ethical considerations and should indicate how the principles set forth in this Declaration have been considered. The protocol should include information on funding, sponsors, institutional affiliations, potential conflicts of interest and incentives for study subjects, and information on provisions for treating or compensating subjects who have suffered harm as a result of their participation in the research.
In clinical trials, the protocol should also describe appropriate arrangements for post-trial stipulations.

F. RESEARCH ETHICS COMMITTEES
23. The research protocol should be submitted for consideration, comment, advice and approval to the relevant research ethics committee prior to the start of the trial. This committee should be transparent in its operation, should be independent of the investigator, the sponsor or any other undue influence, and should be appropriately qualified. The committee should consider the laws and regulations in force in the country where the research is conducted, as well as applicable international standards, but these

should not be allowed to diminish or eliminate any of the protections for research subjects set out in this Declaration.
The committee has the right to monitor ongoing trials. The investigator has the obligation to provide monitoring information to the committee, especially on any serious adverse events. No amendments to the protocol should be made without the consideration and approval of the committee. After the study is completed, the investigators should submit a final report to the committee with a summary of the results and conclusions of the study.

G. PRIVACY AND CONFIDENTIALITY

24. Every precaution should be taken to protect the privacy of the research participant and the confidentiality of his or her personal information.

H. INFORMED CONSENT

25. The participation of persons capable of giving informed consent in medical research must be voluntary. While it may be appropriate to consult with family members or community leaders, no person capable of giving informed consent should be included in a study unless he or she freely consents.
26. In medical research involving human subjects capable of giving informed consent, each potential subject must receive adequate information about the aims, methods, sources of funding, potential conflicts of interest, institutional affiliations of the investigator, anticipated benefits, foreseeable risks and discomforts arising from the experiment, post-study stipulations and any other relevant aspects of the research. The potential subject should be informed of the right to participate or not to participate in the research and to withdraw consent at any time without risk of reprisal. Special attention should be given to the specific information needs of each potential subject, as well as to the methods used to deliver the information.
After ensuring that the individual has understood the information, the physician or other appropriately qualified person must then seek, preferably in writing, the individual's voluntary informed consent. If consent cannot be given in writing, the process for achieving it should be formally documented and witnessed.
All persons participating in medical research should have the option of being informed about the overall results of the study.
27. In seeking informed consent for participation in research, the physician should take special care when the potential subject is related to him or her by a dependent relationship or if he or she consents under duress. In such a situation, informed consent should be sought by an appropriately qualified person who has nothing to do with that relationship.
28. When the potential subject is incapable of giving informed consent, the physician must seek the informed consent of the legally authorized representative. Such persons should not be included in research that is not likely to be of benefit to them unless the research is intended to promote the health of the group represented by the potential subject and the

research cannot be conducted on persons capable of giving informed consent and the research involves only minimal risk and minimal cost.

29. If a potential research subject deemed incapable of giving informed consent is able to give assent to participate or not to participate in the research, the physician should request this, in addition to the consent of the legally authorized representative. The consent of the potential subject must be respected.

30. Research on individuals who are physically or mentally incapable of giving consent, e.g. unconscious patients, can be performed only if the physical/mental condition that prevents informed consent is a necessary characteristic of the research group. In these circumstances, the physician should seek informed consent from the legally authorized representative. If such a representative is not available and if the research cannot be delayed, the study may be conducted without informed consent, provided that the specific reasons for including individuals with a condition that prevents them from giving informed consent have been stated in the research protocol and the study has been approved by a research ethics committee. Consent to remain in the research should be obtained as soon as possible from the individual or a legal representative.

31. The physician must fully inform the patient of the research-related aspects of care. A patient's refusal to participate in an investigation or decision to withdraw should never adversely affect the patient-physician relationship.

32. For medical research involving identifiable human material or data, such as research on material or data contained in biobanks or similar repositories, the physician must seek informed consent for collection, analysis, storage and re-use. There may be exceptional situations in which it will be impossible or impracticable to obtain consent for such research. In this situation, the research may only be conducted after consideration and approval by a research ethics committee.

I. USE OF PLACEBO

33. The potential benefits, risks, costs and effectiveness of any new intervention should be assessed by comparison with the best proven interventions, except in the following circumstances:

- Where there is no proven intervention, the use of a placebo, or no intervention at all, is acceptable, or
- When for methodological, scientific and compelling reasons, the use of any intervention less effective than the best proven, the use of a placebo or no intervention is necessary to determine the efficacy and safety of an intervention.
- and patients who receive any intervention that is less effective than the best proven intervention, placebo or no intervention, will not suffer additional risks, serious adverse effects or irreversible harm as a consequence of not receiving the best proven intervention.

Extreme care must be taken to avoid overuse of this option.

J. POST-TEST STIPULATIONS

34. Prior to the clinical trial, sponsors, investigators, and host country governments should provide for post-trial access to all participants who still need an intervention that has been identified as beneficial in the trial. This information should also be provided to participants during the informed consent process.

K. REGISTRATION AND PUBLICATION OF RESEARCH AND DISSEMINATION OF RESULTS

35. All research studies involving human subjects should be registered in a publicly available database before the first subject is accepted.

36. Researchers, authors, sponsors, editors and publishers all have ethical obligations with respect to the publication and dissemination of the results of their research. Researchers have a duty to make the results of their human subjects research publicly available and are responsible for the integrity and accuracy of their reports. Ethical standards of reporting must be accepted by all parties. Negative as well as positive results must be published or otherwise made publicly available. The source of funding, institutional affiliations and conflicts of interest should be disclosed in the publication. Research reports that do not adhere to the principles outlined in this Declaration should not be accepted for publication.

L. UNPROVEN INTERVENTIONS IN CLINICAL PRACTICE

37. When proven interventions are not available in the care of a patient or other known interventions have proven ineffective, the physician, after seeking expert advice, with the informed consent of the patient or a legally authorized representative, may be permitted to use unproven interventions if, in the physician's judgement, this gives some hope of saving life, restoring health or alleviating suffering. Such interventions should be further investigated in order to assess their safety and efficacy. In all cases, such new information should be recorded and, where appropriate, made publicly available.
