# Supplementary material for: Study protocol for a randomized clinical trial to assess 7 versus 14-days of treatment for Pseudomonas aeruginosa bloodstream infections (SHORTEN-2 trial)
Source: PLoS One. 2022 Dec 22;17(12):e0277333. doi: 10.1371/journal.pone.0277333 (PMC9778939; doi:10.1371/journal.pone.0277333)
Supplement: S6 File — (PDF) [file pone.0277333.s006.pdf]

PROTOCOLO DE ENSAYO CLÍNICO

"Eficacia y seguridad de 7 versus 14 días de tratamiento antibiótico para la bacteriemia producida por *Pseudomonas aeruginosa*: un ensayo clínico multicéntrico, aleatorizado (SHORTEN-2) con un análisis DOOR/RADAR"

CÓDIGO: SHORTEN II

Nº EUDRACT: 2021-003847-10

VERSIÓN: 2.0 de 17 de enero de 2022

Historial de versiones del protocolo de estudio.

Fecha	Versión	Cambios	Autores
15 octubre 2021	1.0		
17 de enero de 2022	2.0		

RESUMEN PROTOCOLO	
TIPO DE SOLICITUD	Ensayo clínico para evaluar la eficacia y la seguridad de 7 vs. 14 días de diferentes antibióticos autorizados en el tratamiento de la bacteriemia por *Pseudomonas aeruginosa.*
IDENTIFICACIÓN DEL PROMOTOR	Fundación Pública Andaluza para la Gestión de la Investigación en Salud de Sevilla (FISEVI).<br>Hospital Universitario Virgen del Rocío.<br>Edificio de laboratorios, 6ª planta.<br>Avda. Manuel Siurot S/N<br>41013/Sevilla<br>Tel. 955012820
TÍTULO DEL ENSAYO	“Eficacia y seguridad de 7 versus 14 días de tratamiento antibiótico para la bacteriemia producida por *Pseudomonas aeruginosa:* un ensayo clínico multicéntrico, aleatorizado (SHORTEN-2) con un análisis DOOR/RADAR”
CÓDIGO DE PROTOCOLO	SHORTEN II
INVESTIGADOR COORDINADOR	Dr. José Miguel Cisneros Herreros<br>UGC de Enfermedades Infecciosas, Microbiología y Medicina Preventiva<br>Hospital Universitario Virgen del Rocío/ Instituto de Biomedicina de Sevilla (IBiS)<br>Avda. Manuel Siurot s/n<br>41013 Sevilla<br><br>Coordinador Clínico<br>Dr. José Molina Gil-Bermejo<br>UGC de Enfermedades Infecciosas, Microbiología y Medicina Preventiva<br>Hospital Universitario Virgen del Rocío/ IBiS<br>Avda. Manuel Siurot s/n<br>41013 Sevilla

	Coordinador Microbiológico<br>Dr. José Antonio Lepe Jiménez<br>UGC de Enfermedades Infecciosas, Microbiología y Medicina Preventiva<br>Hospital Universitario Virgen del Rocío/ IBiS<br>Avda. Manuel Siurot s/n<br>41013 Sevilla<br><br>Coordinadora Farmacéutica Clínica<br>Dra. Ana Belén Guisado Gil<br>UGC de Farmacia Hospitalaria<br>Hospital Universitario Virgen del Rocío/ IBiS<br>Avda. Manuel Siurot s/n<br>41013 Sevilla<br><br>Coordinadora Logística de las muestras y de la Investigación experimental<br>Dra. María Eugenia Pachón Ibáñez<br>UGC de Enfermedades Infecciosas, Microbiología y Medicina Preventiva<br>Hospital Universitario Virgen del Rocío/ IBiS<br>Avda. Manuel Siurot s/n<br>41013 Sevilla<br><br>Coordinadora/monitor médico y de seguridad<br>Dra. Clara M Rosso Fernández<br>UICEC-HUVR, CDCA<br>Hospital Universitario Virgen del Rocío/ IBiS<br>Avda. Manuel Siurot s/n<br>41013 Sevilla
CENTROS EN LOS QUE SE PREVÉ REALIZAR EL ENSAYO	Estudio multicéntrico en el que participan 30 hospitales españoles. Anexo II
COMITÉ/S ÉTICO/S DE INVESTIGACIÓN CLÍNICA	Comité de referencia: CEIm provincial de Sevilla.

RESPONSABLE DE LA MONITORIZACIÓN	Unidad de Investigación Clínica y Ensayos Clínicos, Hospital Universitario Virgen del Rocío-SCReN (*Spanish Research Network*).
TRATAMIENTO EXPERIMENTAL Y CONTROL	Los pacientes del estudio recibirán el tratamiento antibiótico elegido según criterio del facultativo responsable del paciente para el tratamiento de la bacteriemia por *P. aeruginosa,* de acuerdo a los protocolos establecidos en el hospital al que pertenezcan.<br><br>La intervención del estudio consiste en el control de la duración del tratamiento antibiótico activo según los grupos que se describen a continuación:<br><br>● **Grupo experimental (brazo corto)**: recibirá siete días de tratamiento antibiótico activo desde el último hemocultivo positivo a *P. aeruginosa* siempre que el paciente haya permanecido durante los tres días previos sin síntomas ni signos de infección.<br><br>● **Grupo control (brazo largo)**: recibirá 14 días de tratamiento antibiótico activo desde el último hemocultivo positivo a *P. aeruginosa* siempre que el paciente haya permanecido durante los tres días previos sin síntomas ni signos de infección.<br><br>Tras la interrupción del tratamiento, éste puede reanudarse si el médico a cargo del paciente lo considera necesario debido a una evolución desfavorable.
FASE DEL ENSAYO CLÍNICO	Fase IV
OBJETIVOS	Objetivo principal: Determinar si un régimen de tratamiento antibiótico de 7 días es superior a un régimen de 14 días en el tratamiento de bacteriemia por *P. aeruginosa*, evaluando de manera integrada tanto la efectividad del régimen corto como su potencial para reducir los acontecimientos adversos graves y la exposición a los antibióticos.

	Objetivos secundarios:<br><br>1. Determinar si el régimen corto es no inferior al régimen prolongado en términos de recurrencia de infección y mortalidad, y si se puede aplicar la interrupción de forma segura a nivel individual utilizando una regla de decisión clínica simple.<br><br>2. Describir los efectos adversos y las sobreinfecciones, específicamente las producidas por bacterias multirresistentes, hongos y *Clostridioides difficile* en ambos regímenes de tratamiento.<br><br>3. Analizar la eficiencia de los regímenes cortos analizando el número de días de tratamiento y días de estancia hospitalaria evitados al final del período de seguimiento.<br><br>4. Comprobar si las recurrencias son por la misma cepa, comparando las cepas de *P. aeruginosa* mediante secuenciación genética.<br><br>5. Comparar el impacto ecológico de los regímenes de tratamiento corto y prolongado analizando la diversidad de la microbiota intestinal.
DISEÑO	Ensayo clínico pragmático, fase IV, abierto, aleatorizado, multicéntrico, para demostrar la superioridad de antibióticos con indicación autorizada durante de 7 días vs. 14 días en el tratamiento de bacteriemia por *P. aeruginosa*.
ENFERMEDAD O TRASTORNO DEL ESTUDIO	Bacteriemia monomicrobiana producida por *P. aeruginosa.*
VARIABLE PRINCIPAL DE VALORACIÓN	La variable principal será la probabilidad de que cualquier paciente del brazo experimental logre mejores

	resultados que un paciente en el grupo de control evaluado a través de su puntuación en el análisis DOOR / RADAR.
POBLACIÓN EN ESTUDIO Y NÚMERO TOTAL DE PACIENTES	La población en estudio estará formada por pacientes adultos (18 años o más) con bacteriemia monomicrobiana producida por *P. aeruginosa*.<br>El tamaño muestral necesario es de 306 pacientes (153 por brazo).
SEGUIMIENTO	Noventa días desde la fecha de extracción del primer hemocultivo positivo.
DURACIÓN DEL TRATAMIENTO	La duración inicial del tratamiento antibiótico activo será de 7 o 14 días, dependiendo del brazo asignado. En los pacientes asignados al brazo corto (experimental), el tratamiento activo se suspenderá el séptimo día si el paciente está apirético y no presenta síntomas de infección en las 72 horas previas. En los pacientes asignados al brazo largo (control), el tratamiento activo se suspenderá el día 14 si el paciente está apirético y no presenta síntomas de infección en las 72 horas previas. De no cumplirse esta regla clínica, el tratamiento debe mantenerse, y el paciente será reevaluado cada 48-72h. Una vez se cumplan los criterios clínicos arriba indicados, se procederá a la interrupción del tratamiento.
CALENDARIO Y FECHA PREVISTA DE FINALIZACIÓN	Se prevé una duración total del ensayo de cuatro años desde que se inicia el periodo de reclutamiento (2022 a 2025). Se incluye un periodo de 3 meses para la presentación de la documentación a la Agencia Española de Medicamentos y Productos Sanitarios (AEMPS) y a los Comités Éticos, así como la formación inicial en los centros participantes. El periodo de reclutamiento de pacientes será de 36 meses y se calculan otros 9 meses para el análisis y posterior difusión de resultados.

1.- ÍNDICE

2.- INFORMACIÓN GENERAL 12
2.1 Identificación del ensayo 12
2.2 Tipo de ensayo clínico 12
2.3 Datos relativos al promotor 12
2.3.1 Persona autorizada por el promotor 12
2.3.2 Responsable de la monitorización 12
2.4 Descripción de los productos en estudio 13
2.5 Datos de los investigadores del estudio 14
2.6 Laboratorios, Departamento Médico o Instituciones relacionadas 14
3.- JUSTIFICACIÓN.......... 15
4.- HIPÓTESIS Y OBJETIVOS DEL ENSAYO 19
4.1 Hipótesis 19
4.2. Objetivos 20
5.- DISEÑO DEL ENSAYO 20
5.1 Variables del ensayo 20
5.1.1 Variable principal de resultado 20
5.1.2 Variables secundarias. 21
5.2 Diseño 23
5.3 Procedimiento de aleatorización 23
5.4 Enmascaramiento 25
5.5 Tratamientos del ensayo 25
5.6 Medicamentos y tratamientos permitidos 27
5.7 Seguimiento de los pacientes 27
5.8 Criterios de finalización o interrupción del estudio 28
5.9 Adquisición, acondicionamiento y etiquetado de la medicación 28
5.10 Almacenamiento y dispensación de la medicación 28
5.11 Final del ensayo 28

6.- CRITERIOS DE SELECCIÓN ... 28
6.1 Criterios de inclusión 29
6.2 Criterios de exclusión 29
6.3 Criterios de retirada 30
6.3.1 Por decisión del paciente 30
6.3.2 Por pérdida de seguimiento 30
6.3.3 Por criterios de seguridad 30
6.3.4 Por incumplimiento o violación de las normas contenidas en el protocolo 31
6.4 Seguimiento de los pacientes retirados prematuramente 31
6.5 Definiciones 31
Nuevo episodio de bacteriemia 33
Reaparición de fiebre 33
Días libres de tratamiento antibiótico 34
Días de hospitalización 34
Acontecimientos adversos graves 34
7.- TRATAMIENTO DE LOS SUJETOS ... 34
7.1 Medicación concomitante 34
7.2 Medicación de rescate 35
7.3 Calendario de visitas y evaluaciones 36
7.4 Procedimientos por visita 37
7.5 Otros procedimientos del estudio (Subestudios) 40
7.5.1 Estudios microbiológicos 40
7.5.2 Estudios de diversidad bacteriana en la microbiota intestinal. 41
8.- VALORACIÓN DE LA EFICACIA ... 41
8.1 Variables de eficacia 41
8.2 Pruebas de laboratorio 42
9.- VALORACIÓN DE SEGURIDAD ... 42
9.1 Evaluaciones de seguridad 42
9.1.1 Exploración física, constantes vitales 42

9.1.2 Pruebas de laboratorio y microbiológicas 43

9.2 Acontecimientos adversos de interés para su seguimiento 43

9.3 Definiciones 43

9.4 Información de seguridad de referencia 45

9.5 Notificación y recogida de acontecimientos adversos graves 45

9.6 Notificación expeditiva de RAGI 46

9.7 Notificación expeditiva de otra información de seguridad relevante 47

9.8 Notificación a los investigadores 47

9.9 Errores de medicación 47

10.- ESTADÍSTICA 48

10.1 Cálculo del tamaño muestral 48

10.2 Análisis estadístico 48

10.3 Poblaciones de análisis 49

10.4 Análisis de seguridad y otros resultados secundarios 50

10.5 Análisis intermedio 50

10.6 Comité independiente de evaluación 51

11.- ASPECTOS ÉTICOS 51

11.1 Consentimiento informado 51

11.2 Protección de datos 52

11.3 Responsabilidades de los participantes en el estudio 52

11.4 Monitorización y auditoría 53

11.5 Finalización prematura o suspensión del estudio 53

11.6 Documentación del estudio 53

12.- FINANCIACIÓN Y SEGURO 53

12.1 Financiación 54

12.2 Seguro 54

13.- POLÍTICA DE PUBLICACIÓN...54

ANEXO I. BIBLIOGRAFÍA...56

ANEXO II: LISTADO DE CENTROS PARTICIPANTES 59

ANEXO III. DIAGRAMA DE FLUJO..61

ANEXO IV. EUCAST 62
ANEXO V. MATERIAL DIDÁCTICO 63
ANEXO VI. CUESTIONARIO PARA VISITAS 3, 4 Y NO PLANIFICADAS........64
ANEXO VIII. HOJA DE INFORMACIÓN AL PACIENTE Y CONSENTIMIENTO INFORMADO. 67
ANEXO IX. ÍNDICE DE CHARLSON 68
ANEXO X. PITT SCORE........69
ANEXO XI. PROCEDIMIENTO DE ENVÍO DE AISLAMIENTOS MICROBIOLÓGICOS Y DE MUESTRAS DE HECES FECALES PARA ESTUDIOS DE MICROBIOTA INTESTINAL. ..70
ANEXO XII: FORMULARIO DE AISLADO 73
ANEXO XIII. FORMULARIO DE NOTIFICACIÓN DE AAG 74
ANEXO XIV. DECLARACIÓN DE HELSINKI DE LA ASOCIACIÓN MÉDICA MUNDIAL ...77

2.- INFORMACIÓN GENERAL

2.1 Identificación del ensayo

Código del estudio: SHORTEN II
EudraCT: 2021-003847-10

2.2 Tipo de ensayo clínico

Ensayo clínico fase IV, abierto, aleatorizado, pragmático, multicéntrico, para demostrar la superioridad de un régimen antibiótico con indicación autorizada durante 7 días *vs.* 14 días en el tratamiento de la bacteriemia por *P. aeruginosa*.

2.3 Datos relativos al promotor

Fundación Pública Andaluza para la Gestión de la Investigación en Salud de Sevilla (FISEVI).
Hospital Universitario Virgen del Rocío.
Edificio de laboratorios, 6ª planta.
Avda. Manuel Siurot S/N
41013 Sevilla
Tel. 955012820

2.3.1 Persona autorizada por el promotor

Dra. Clara M. Rosso Fernández
Unidad de Investigación Clínica y Ensayos Clínicos
Hospital Universitario Virgen del Rocío
Avda. Manuel Siurot s/n
41013 Sevilla
Tel.:955313414
Fax: 954232992

2.3.2 Responsable de la monitorización

Unidad de Investigación Clínica y Ensayos Clínicos, Hospital Universitario Virgen del Rocío-SCReN (Spanish Research Network).
Hospital Universitario Virgen del Rocío
Avda. Manuel Siurot S/N
41013 Sevilla
Tel.:955313414
Fax: 955095338

2.4 Descripción de los productos en estudio

- **Grupo experimental (brazo corto)**: régimen corto de siete días con un tratamiento antibiótico activo.
- **Grupo control (brazo largo)**: régimen largo de 14 días con un tratamiento antibiótico activo.

Se considerará **tratamiento antibiótico activo** cualquier tratamiento con actividad *in vitro* probada frente a la cepa responsable de la bacteriemia del paciente, independientemente de la dosis administrada.

A continuación, se detalla en la **Tabla 1** el listado de tratamientos que se considerarán activos en función de las CMI del microorganismo aislado:

Tabla 1. Listado de tratamientos activos en función de las CMI del microorganismo aislado.

**Tratamientos antibióticos activos**	**CMI microorganismo aislado**
Ciprofloxacino	CMI ≤0,5 mg/L
Levofloxacino	CMI ≤1 mg/L
Piperacilina/tazobactam	CMI ≤16 mg/L
Ceftazidima	CMI ≤8 mg/L
Cefepima	CMI ≤8 mg/L
Ceftazidima/avibactam	CMI ≤8 mg/L
Ceftolozano/tazobactam	CMI ≤4 mg/L
Imipenem	CMI ≤4 mg/L
Meropenem	CMI ≤ 8 mg/L
Meropenem-vaborbactam	CMI ≤8 mg/L
Colistina	CMI ≤2 mg/L
Amikacina	CMI ≤16 mg/L
Tobramicina	CMI ≤2 mg/L
Aztreonam	CMI ≤16 mg/L

Una vez el paciente sea aleatorizado, las dosis serán optimizadas en caso de que no se hubiera hecho previamente a la aleatorización, de acuerdo con las recomendaciones EUCAST reflejadas en la **Tabla 2**:

Tabla 2. Listado de la posología de las dosis optimizadas de los tratamientos antibióticos activos.

Tratamientos antibióticos activos	Posología dosis optimizada
Ciprofloxacino	750 mg/12h oral o 400 mg/8h i.v.
Levofloxacino	500 mg/12h oral o i.v.
Piperacilina/tazobactam	4 g/0.5 g/8h administrado en perfusión extendida de 4 h.<br>4g/0.5 g/6h en perfusión extendida en el caso de cepas resistentes a ceftazidima o en neumonías.
Ceftazidima	2 g/8h i.v.
Cefepima	2 g/8h i.v.
Ceftazidima/avibactam	2 g/8h i.v. administrado en perfusión de 2 h
Ceftolozano/tazobactam	1 g/0.5 g/8h. Para neumonía, la dosis apropiada es 2 g/1g/8h.
Imipenem	1g/ 6h i.v.
Meropenem	CMI ≤2 mg/L: 1g/8h i.v.<br>CMI >2 mg/L y ≤8 mg/L: 2g/8h i.v en perfusion de 3h.
Meropenem-vaborbactam	2 g/2 g cada 8h en perfusión de 3 h.
Colistina	4,5 MU/12h i.v.
Amikacina	25-30 mg/kg/24h i.v.
Tobramicina*	6-7 mg/kg/24h i.v.
Aztreonam	2 g/6h i.v.

* De manera específica la monoterapia con aminoglucósidos no será considerada como tratamiento activo, excepto para el tratamiento de pielonefritis aguda.

2.5 Datos de los investigadores del estudio

Ver Anexo II

2.6 Laboratorios, Departamento Médico o Instituciones relacionadas

Unidad de Gestión Clínica de Enfermedades Infecciosas, Microbiología y Medicina Preventiva.
Hospital Universitario Virgen del Rocío/ Instituto de Biomedicina de Sevilla (IBiS)
Avenida Manuel Siurot s/n
41013 Sevilla
Tel. 955012820

Unidad de Gestión Clínica de Farmacia
Hospital Universitario Virgen del Rocío

Avenida Manuel Siurot s/n
41013 Sevilla
Tel. 955012095

Unidad de Investigación Clínica y Ensayos Clínicos, Hospital Universitario Virgen del Rocío-SCReN (*Spanish Research Network*). Hospital Universitario Virgen del Rocío
Avda. Manuel Siurot S/N
41013 Sevilla
Tel.:955313414
Fax: 955095338

Servicio de Microbiología.
Hospital Universitario Virgen Macarena
Avenida Dr. Fedriani, 3
41009 Sevilla
Tel. 955926552

CIBER de Enfermedades Infecciosas

3.- JUSTIFICACIÓN

Las infecciones por bacterias multirresistentes (BMR) son una de las mayores amenazas para la salud pública mundial (1). Por ello, la Unión Europea y los países miembros, incluida España, han puesto en marcha planes de lucha contra las BMR (2,3).

Mejorar el uso de los antibióticos es un objetivo clave de estos planes porque el uso inapropiado de los mismos, con frecuencia excesivo y en ocasiones injustificado, es un factor de riesgo determinante en el desarrollo de BMR (2,3).

Los programas de optimización de antibióticos (PROA) son una de las intervenciones más eficaces para conseguir mejorar el uso de los antibióticos (4) y al hacerlo de manera intensa y sostenida logran reducir las resistencias bacterianas tanto en hospital (5) como en atención primaria (6).

Administrar la duración justa de tratamiento antibiótico es una de las principales áreas de mejora de los PROA porque evita el sobretratamiento y sus consecuencias negativas tanto a nivel individual como poblacional. Desafortunadamente la duración óptima del tratamiento antibiótico es aún desconocida en muchas de las infecciones más comunes. Esto es debido a que los ensayos clínicos realizados para la aprobación de los antibióticos disponibles, se diseñaron para conocer la eficacia y la seguridad de los mismos, no la duración óptima.

Por todo lo anterior, la investigación clínica sobre la duración del tratamiento antibiótico tiene gran interés. Para conocerla, varios ensayos clínicos se han llevado a cabo en diferentes enfermedades infecciosas, incluidas la neumonía adquirida en la comunidad (7); la neumonía asociada a ventilación mecánica (8); pielonefritis (9); infección intraabdominal (10), neutropenia febril postquimioterapia (11) y la bacteriemia por *Enterobacterales* (12, 13, 14) demostrando que las pautas cortas de tratamiento no son inferiores a las pautas largas recomendadas tradicionalmente.

Las infecciones por *P. aeruginosa* y, particularmente la bacteriemia, que es el objeto de este proyecto, son frecuentes y tienen mal pronóstico.

P. aeruginosa se encuentra entre las especies bacterianas que más frecuentemente causan infecciones invasivas en humanos. Estas infecciones suelen afectar a pacientes con comorbilidades y situaciones relacionadas con la asistencia sanitaria como los pacientes inmunodeprimidos por cualquier causa: receptores de trasplante, pacientes hematológicos y oncológicos, grandes quemados, cirugía mayor y en pacientes ingresados en unidades de cuidados intensivos, y se asocian a una alta mortalidad, que oscila entre el 12 y el 60% en diversos estudios sobre bacteriemias (15). *P. aeruginosa* es una causa frecuente de bacteriemia, que se asocia con una alta morbi-mortalidad. Las opciones terapéuticas frente a estas infecciones son limitadas por varias razones: *P. aeruginosa* es intrínsecamente resistente a la mayoría de los antibióticos utilizados frente a otras bacterias Gram negativas, desarrolla con frecuencia resistencia durante el tratamiento (15) y, además, se ha producido un aumento de la incidencia de cepas resistentes a múltiples fármacos (MDR) y extensivamente resistentes a fármacos (XDR) en los últimos años (16).

La duración del tratamiento antibiótico para la bacteriemia causada por *P. aeruginosa* ha respondido tradicionalmente a las opiniones de los expertos. Diferentes sociedades científicas proponen tratar las bacteriemias Gram negativas relacionadas con catéter, incluida *P. aeruginosa*, con una duración variable de 7 a 14 días (19,20) y no existen indicaciones de otras fuentes. Un estudio reciente sugirió que ciclos más cortos de antibióticos para tratar la bacteriemia por *P. aeruginosa* podrían ser igualmente eficaces que ciclos más prolongados en términos de riesgo de mortalidad o recurrencia a 30 días (21). Sin embargo, el diseño de este estudio observacional (una cohorte retrospectiva de 249 pacientes con tratamientos de diferente duración) no es lo suficientemente sólido como para obtener conclusiones que se puedan aplicar a la práctica clínica.

El riesgo de recurrencia de la infección es el argumento más utilizado para justificar la recomendación de prolongar el tratamiento en las infecciones producidas por *P. aeruginosa*. Está basado en un ensayo clínico realizado en 401 pacientes con neumonía asociada a ventilación mecánica por diferentes etiologías que demostró que el tratamiento

antibiótico durante 8 días tuvo una eficacia clínica comparable al de 14 días en cuanto a mortalidad y recurrencia, excepto en las neumonías producidas por bacilos Gram negativos no fermentadores, incluida *P. aeruginosa*, en las que la recurrencia fue más frecuente en el grupo de tratamiento corto, siendo igual la mortalidad (8). Estos resultados tienen limitaciones por tamaño muestral insuficiente y la dificultad para distinguir si la *P. aeruginosa* aislada en la muestra de las secreciones respiratorias de la supuesta recurrencia producía infección o era una colonización del tubo endotraqueal.

En la bacteriemia por *P. aeruginosa* la recurrencia es relativamente frecuente y tiene peor pronóstico. En una serie de 290 pacientes con bacteriemia por *P. aeruginosa*, la recurrencia de la bacteriemia sucedió en el 15% de los episodios en los 30 días que siguieron a la suspensión del tratamiento (20). La recurrencia de la bacteriemia por *P. aeruginosa* se asoció a peor pronóstico que el episodio primario de bacteriemia (18 vs 7% de mortalidad) en otra serie de 441 pacientes (22). La ausencia de tipificación molecular de los aislados es una limitación mayor de estos resultados que no permite confirmar si los segundos aislados fueron recurrencias o reinfecciones. Las reinfecciones podrían ser más comunes en los pacientes de alto riesgo de bacteriemia por *P. aeruginosa*, como son los pacientes hematológicos con neutropenia postquimioterapia. La frecuencia de este hecho es desconocida.

La duración óptima del tratamiento antibiótico de los pacientes con bacteriemia por *P. aeruginosa* es desconocida. Se necesitan ensayos clínicos bien diseñados para conocerla. El diseño tradicional de los ensayos clínicos de no inferioridad tiene limitaciones para valorar diferentes estrategias de tratamiento antibiótico, pauta corta *vs.* larga, principalmente por la falta la integración de los beneficios y los daños a nivel del paciente. Con este diseño tradicional, las variables de eficacia y seguridad se han analizado por separado, pero este enfoque puede evitar que los investigadores detecten grupos de pacientes con una superposición significativa de estos efectos (es decir, mayor eficacia a expensas de un aumento de los eventos adversos) (23).

El diseño DOOR (*Desirability of Outcome Ranking)/*RADAR (*Response Adjusted for Duration of Antibiotic Risk*) es un novedoso diseño de estudio en la evaluación de los riesgos y beneficios de nuevas estrategias para optimizar el uso de antibióticos para superar la mayoría de estas deficiencias

RADAR es una metodología que utiliza un diseño de superioridad y un proceso de 2 pasos: Primero, ordenar a los pacientes en función de los resultados clínicos globales (riesgo/beneficio), y segundo, clasificar a los pacientes con según una clasificación de desenlaces deseables o DOOR. Los DOORs se construyen asignando rangos más altos a los pacientes con, primero, mejores resultados clínicos globales y, segundo, con duraciones más cortas de uso de antibióticos para resultados clínicos globales similares. Las estrategias de uso de antibióticos se comparan a través de las clasificaciones DOOR.

Se estima la probabilidad de que un paciente seleccionado al azar tenga un mejor DOOR si se le asigna una nueva estrategia.

Una limitación de este análisis es que, al igual que otras variables compuestas, la ventaja de una estrategia en el análisis DOOR no implica necesariamente una ventaja en todas las variables; de hecho, desventajas en variables específicas son posibles.

El método DOOR / RADAR es una mejora significativa con respecto a las medidas anteriores, de resultados logrados por los PROA. Proporciona un enfoque innovador del impacto clínico de una intervención determinada, ponderando su eficacia y los posibles daños sobre los pacientes de una manera intuitiva para los médicos (23). Además, su metodología puede facilitar el diseño de ensayos clínicos de superioridad a través de tamaños de muestra más accesibles (24).

El análisis DOOR/RADAR ha sido utilizado para evaluar el impacto del tratamiento corto en la enfermedad meningocócica invasiva (25), en la infección intraabdominal (26) y en la neumonía adquirida en la comunidad (27) pero hasta ahora pero no se han diseñado ensayos clínicos con este análisis innovador.

En los últimos años, el reciente aumento en el conocimiento sobre la microbiota intestinal humana ha cambiado nuestra visión sobre los antibióticos. Por lo tanto, los antibióticos ya no se consideran solo medicamentos beneficiosos, sino también potencialmente dañinos, ya que su abuso parece desempeñar un papel en la patogénesis de varios trastornos asociados con el deterioro de la microbiota (por ejemplo, infección por *C. difficile* o trastornos metabólicos) (28). Esta nueva percepción ha desencadenado estudios orientados a conocer cómo la clase de antibióticos, la dosis, la vía de administración y la duración del tratamiento afectan a la microbiota intestinal y, por lo tanto, al resultado de los pacientes (29).

En una revisión sistemática reciente, que estudia cómo los antibióticos inducen cambios en la microbiota intestinal humana para los antibióticos más comúnmente recetados en la atención primaria en el Reino Unido (30), concluyeron que los antibióticos tienen un impacto en la microbiota intestinal, reduciendo rápidamente la diversidad bacteriana, lo que lleva a la disbiosis. Después del tratamiento, las bacterias intestinales son capaces de cierto grado de recuperación a su estado basal en la mayoría de los individuos, pero estos estudios no consideran la recuperación en los ancianos u otras poblaciones. Se analizó el efecto sobre cómo la diversidad bacteriana del tracto intestinal impacta en la mortalidad y el desenlace de varias enfermedades como el cáncer o en el seguimiento del trasplante alogénico de células madre hematopoyéticas (31,32), sin embargo, no existe ningún estudio que analice el efecto de la duración de los antibióticos en la microbiota intestinal de pacientes con bacteriemia por *P. aeruginosa*.

El objetivo del presente ensayo será demostrar que un curso de antibióticos de 7 días es mejor que los esquemas tradicionales de 14 días para tratar a los pacientes con bacteriemia por *P. aeruginosa*, en términos de reducción a la exposición a antibióticos de los pacientes y, por lo tanto, sus efectos secundarios, al tiempo que se logran resultados clínicos similares.

Justificación del diseño

La duración óptima del tratamiento antibiótico en la bacteriemia por *P. aeruginosa* es desconocida, no existen ensayos clínicos que lo hayan estudiado. Por ello, el tiempo de tratamiento actualmente recomendado se basa en opiniones de expertos, y es tan impreciso que varía entre 7 y 14 días.

Basados en datos procedentes de ensayos clínicos realizados en otras bacterias diferentes a *P. aeruginosa*, que demuestran que pautas de tratamiento con duración más corta podrían ser tan eficaces y seguras como las pautas más prolongadas actualmente recomendadas, se diseña este proyecto de investigación para realizar el primer ensayo clínico que trate este importante problema de salud, y dé respuesta a la hipótesis de que los tratamientos antibióticos actuales son demasiado largos y, por lo tanto, menos seguros para el paciente, porque está más tiempo expuesto a sus efectos secundarios directos, al riesgo de desarrollar bacterias resistentes a los antibióticos utilizados y otras sobreinfecciones y finalmente, por tener estancias hospitalarias más prolongadas.

Este ensayo clínico utilizará una metodología innovadora para el análisis de los resultados, la llamada DOOR/RADAR, que permite comprobar que la duración óptima del tratamiento antibiótico se asocia con el mejor pronóstico de las personas con esta infección.

4.- HIPÓTESIS Y OBJETIVOS DEL ENSAYO

4.1 Hipótesis

En pacientes con bacteriemia producida por *Pseudomonas aeruginosa* el tratamiento antibiótico activo con una duración de 7 días es superior al de 14 días.

<u>Hipótesis específicas</u>:

1. El régimen corto es mejor que el prolongado en cuanto a días sin tratamiento antibiótico y riesgo de acontecimientos adversos, sobreinfecciones y días de estancia hospitalaria
2. El régimen corto es no inferior al prolongado en cuanto a recurrencia de la bacteriemia y mortalidad, y se puede aplicar de manera segura la interrupción del antibiótico a nivel individual siguiendo una regla de decisión clínica simple.
3. Las bacteriemias recurrentes por *P. aeruginosa* son, en algunos casos, reinfecciones por cepas distintas de la inicial.
4. El régimen corto tendrá un menor impacto ecológico, produciendo una menor reducción de la biodiversidad bacteriana de la microbiota intestinal.

4.2. Objetivos

Objetivo principal: Determinar si un régimen de tratamiento antibiótico de 7 días es superior a un régimen de 14 días en el tratamiento de bacteriemia por *P. aeruginosa*, evaluando de manera integrada tanto la efectividad del régimen corto como su potencial para reducir los acontecimientos adversos graves y la exposición a antibióticos.

Objetivos secundarios:

1. Determinar si el régimen corto es no inferior al régimen prolongado en términos de recurrencia de infección y mortalidad, y si la interrupción del tratamiento antibiótico se puede aplicar de forma segura a nivel individual utilizando una regla de decisión clínica simple.

2. Describir los efectos adversos y sobreinfecciones, y específicamente los producidos por bacterias multirresistentes y *C. difficile* en ambos regímenes de tratamiento.

3. Analizar la eficiencia de los regímenes cortos en cuanto al número de días de tratamiento y días de estancia hospitalaria evitados al final del período de seguimiento.

4. Determinar los factores de riesgo relacionados con el fracaso de tratamiento y el riesgo de recurrencias.

5. Confirmar la recurrencia de infecciones mediante la secuenciación de los aislados de *P. aeruginosa* que ocurran durante el seguimiento.

6. Comparar el efecto de los regímenes de tratamiento corto y prolongado sobre la preservación de la diversidad de la microbiota intestinal.

5.- DISEÑO DEL ENSAYO

5.1 Variables del ensayo

5.1.1 Variable principal de resultado

La variable principal será la probabilidad de que cualquier paciente del brazo experimental logre mejores resultados que un paciente en el grupo de control evaluado a través de su puntuación en el análisis DOOR / RADAR. Este análisis categoriza a los pacientes en dos pasos:

a) Una primera clasificación ordinal de resultados clínicos (DOOR), definida por las siguientes categorías mutuamente excluyentes, evaluadas al final del seguimiento:
1. Curación sin incidencias.
2. Curación con recurrencia probada o probable.
3. Curación con un evento adverso grave.
4. Sin curación clínica.
5. Muerte.

b) Una segunda clasificación en la que los pacientes de la misma categoría de resultado clínico se clasifican según número de días de tratamiento con antibióticos (RADAR), definido como el número de días de tratamiento antimicrobiano desde el momento de la extracción del primer hemocultivo positivo y hasta 30 días después de la interrupción del tratamiento del ensayo. En la medición del número de días de tratamiento antimicrobianos se incluirán todos los antibióticos y antifúngicos indicados durante ese período como tratamiento empírico o dirigido de sobreinfecciones ocurridas durante ese período de seguimiento. No se incluirán los antimicrobianos indicados como profilaxis.

Por tanto, el rango de resultado se establece como criterio de clasificación prioritario, subordinando la valoración de la duración del tratamiento a pacientes con igual resultado clínico. Así, los pacientes con una puntuación DOOR / RADAR más baja serán aquellos con mejores resultados en términos de efectividad clínica, así como una exposición reducida al tratamiento antibiótico.

El resultado final se expresará como la probabilidad de que un paciente del grupo experimental haya obtenido una mejor puntuación que un paciente del grupo control en la escala DOOR/RADAR. Los resultados se considerarán favorables para la intervención estudiada siempre que este porcentaje supere el 50%.

5.1.2 Variables secundarias.

Tabla 3. Listado de las variables secundarias del estudio y el período de seguimiento de las mismas.

Variable	Periodo de seguimiento
Fracaso del tratamiento	Día +30 desde la interrupción del tratamiento del ensayo.<br>Día +90 desde la fecha de extracción del primer hemocultivo positivo.
Análisis DOOR	Día +30 desde la interrupción del tratamiento del ensayo.

	Día +90 desde la fecha de extracción del primer hemocultivo positivo.
Mortalidad por cualquier causa	Día +30 desde la interrupción del tratamiento del ensayo.<br>Día +90 desde la fecha de extracción del primer hemocultivo positivo.
Curación clínica	Día +30 desde la interrupción del tratamiento del ensayo.<br>Día +90 desde la fecha de extracción del primer hemocultivo positivo.
Recurrencias probadas, probables o posibles	Día +30 desde la interrupción del tratamiento del ensayo.<br>Día +90 desde la fecha de extracción del primer hemocultivo positivo.
Nuevo episodio de bacteriemia	Día +30 desde la interrupción del tratamiento del ensayo.<br>Día +90 desde la fecha de extracción del primer hemocultivo positivo.
Reaparición de la fiebre	Día +30 desde la interrupción del tratamiento del ensayo.<br>Día +90 desde la fecha de extracción del primer hemocultivo positivo.
Sobreinfecciones	Día +30 desde la interrupción del tratamiento del ensayo.<br>Día +90 desde la fecha de extracción del primer hemocultivo positivo.
Eventos adversos graves	Día +30 desde la interrupción del tratamiento del ensayo.<br>Día +90 desde la fecha de extracción del primer hemocultivo positivo.<br>Ponderado por 1.000 días de seguimiento.
Días libres de tratamiento antibiótico al final del seguimiento	Día +30 desde la interrupción del tratamiento del ensayo.

	Día +90 desde la fecha de extracción del primer hemocultivo positivo.
Número total de días de hospitalización	Día +30 desde la interrupción del tratamiento del ensayo.<br><br>Día +90 desde la fecha de extracción del primer hemocultivo positivo.
Recuperación de la capacidad funcional basal	Día +30 desde la interrupción del tratamiento del ensayo.<br><br>Día +90 desde la fecha de extracción del primer hemocultivo positivo.

5.2 Diseño

Ensayo clínico fase IV, abierto, aleatorizado, pragmático, multicéntrico, para demostrar la superioridad/no inferioridad de un régimen de antibióticos con indicación autorizada durante de 7 días *vs*. 14 días en el tratamiento de bacteriemia por *P. aeruginosa*.

a. Grupo experimental (brazo corto): recibirá siete días de tratamiento antibiótico activo desde el último hemocultivo positivo a *P. aeruginosa* siempre que cumpla la regla de decisión clínica para la interrupción del tratamiento.

b. Grupo control (brazo largo): recibirá 14 días de tratamiento antibiótico activo desde el último hemocultivo positivo a *P. aeruginosa* siempre que cumpla la regla de decisión clínica para la interrupción del tratamiento.

5.3 Procedimiento de aleatorización

La aleatorización se realizará en el 6º día (+/- 24h) desde el inicio del tratamiento antibiótico activo.

Los pacientes que antes del momento de la aleatorización hubieran recibido un tratamiento activo a dosis no optimizadas según criterios de EUCAST, podrán ser incluidos en el ensayo. En este caso, la dosis será optimizada en el momento de la aleatorización, sin precisar prolongar la duración total del tratamiento prevista en el ensayo.

Los pacientes se detectarán a partir de la revisión diaria de los resultados de hemocultivos. En aquellos pacientes con aislamiento de *Pseudomonas aeruginosa* en el mismo, se

procederá a comprobar los criterios de inclusión y exclusión del estudio y a solicitar el consentimiento informado. Los pacientes que cumplan todos los criterios de inclusión, pero tengan alguno de exclusión se considerarán fallo de *screening*, y el motivo de la exclusión deberá ser recogido en el cuaderno de recogida de datos correspondiente.

Para facilitar la inclusión de pacientes en el estudio se realizará el siguiente procedimiento.

1. Identificación diaria de todos los hemocultivos positivos para *P. aeruginosa* por el Microbiólogo responsable del estudio en cada centro.

2. Valoración clínica de los pacientes identificados con bacteriemia por *P. aeruginosa* por el clínico responsable del estudio para ver si el paciente cumple los criterios de inclusión y de exclusión.

3. A los pacientes que cumplan los criterios de inclusión y no tengan ninguno de exclusión, el Clínico responsable del estudio en cada centro les pedirá el consentimiento informado.

4. Los pacientes que cumplan los criterios de inclusión, pero tengan alguno de exclusión se considerarán fallo de *screening* y el motivo de exclusión debe ser recogido.

5. Los pacientes que no cumplan los criterios de inclusión, no serán incluidos en el ensayo. El/los criterio/s que no cumplen deben ser recogidos.

Será imprescindible disponer del antibiograma de la cepa aislada realizado en base a alguno de los métodos recomendados por EUCAST.

Una vez firmado el consentimiento informado y después de evaluar todos los criterios de elegibilidad, la aleatorización se llevará a cabo en el sexto día desde inicio del tratamiento antibiótico (+/-24h), en una proporción de 1:1, al brazo experimental o al brazo control. La aleatorización será simple y estratificada por centros y por origen pulmonar/extrapulmonar para la bacteriemia (ver detalles en el diagrama de flujo, anexo III).

Se generará una lista de aleatorización utilizando el software Epidat 4.0 y se integrará en el cuaderno de recogida de datos (eCRD), de forma que sólo después del registro de todos los criterios de elegibilidad se revelará al investigador la asignación del brazo al que pertenece el paciente, así como un código para identificar al mismo, compuesto por un código numérico que identifica centro y paciente (ej. XX-XX).

Solo el personal a cargo de este ensayo en la Unidad de Ensayos Clínicos y el equipo de soporte de IT responsable del eCRD tendrá acceso a la lista de aleatorización.

5.4 Enmascaramiento

Se ha elegido un diseño abierto por razones pragmáticas; además, el enmascaramiento de un gran número de diferentes medicamentos que habría que llevar a cabo sería logísticamente inviable.

Al no haber enmascaramiento alguno, tampoco es aplicable ningún procedimiento de rotura del ciego.

Para evitar en la medida de lo posible el sesgo que el carácter abierto del ensayo pudiera generar se han establecido las siguientes medidas:

- Se han establecido criterios de clasificación objetivos para la evaluación de las variables primarias y secundarias.
- La evaluación de las variables de resultado será realizada por un comité independiente ciego (que no forme parte del estudio como investigadores) respecto de la asignación del tratamiento. Dicho comité estará formado por 3 investigadores expertos pertenecientes a CIBER de Enfermedades Infecciosas y tomará sus conclusiones por consenso. Esta evaluación independiente es esencial en el único indicador de resultado que no es objetivo: el diagnóstico de recurrencia probable.Por último, se llevarán a cabo análisis estadísticos de los resultados obtenidos en un conjunto de datos ciego en el que los datos relativos a la asignación a los brazos de tratamiento serán eliminados.

5.5 Tratamientos del ensayo

a. Grupo experimental (brazo corto): recibirá siete días de tratamiento antibiótico activo desde el último hemocultivo positivo siempre que cumpla la regla de decisión clínica para la interrupción del tratamiento.

b. Grupo control (brazo largo): recibirá 14 días de tratamiento antibiótico activo desde el último hemocultivo positivo a *P. aeruginosa* siempre que cumpla la regla de decisión clínica para la interrupción del tratamiento.

Se considerará tratamiento antibiótico activo cualquier tratamiento con actividad *in vitro* probada de acuerdo con las recomendaciones de EUCAST frente a la cepa responsable de la bacteriemia del paciente, independientemente de la posología o forma de administración (**Tabla 3**).

Una vez el paciente sea aleatorizado, el tratamiento será optimizado cuando sea necesario, según se define en la lista de la **Tabla 4**, de acuerdo con las recomendaciones EUCAST (Anexo IV).

De manera específica la monoterapia con aminoglucósidos no será considerada apropiada excepto para el tratamiento de pielonefritis aguda.

a) En el **grupo experimental (brazo corto)** la duración del tratamiento antibiótico activo será de 7 días desde la fecha de extracción del último hemocultivo positivo, siempre que no existan ya síntomas y signos de infección en las 72 horas previas. Durante este periodo de tiempo se admite modificar el antibiótico inicial (simplificación del tratamiento empírico tras obtener los resultados del antibiograma, continuación de tratamiento intravenoso a tratamiento oral, modificación del agente por acontecimientos adversos, etc.) siempre que el nuevo antibiótico sea una opción terapéutica apropiada para el tratamiento de la infección en cuestión. En caso de que el paciente no cumpla con los criterios clínicos necesarios para la retirada, deberá ser reevaluado en intervalos sucesivos de 48-72h hasta documentar que cumple estos criterios, procediéndose entonces a suspender el tratamiento.

b) En el **grupo control (brazo largo)** la duración total del tratamiento antibiótico activo será de 14 días desde el último hemocultivo positivo, siempre que no existieran ya síntomas y signos de infección en las 72h previas. Durante este periodo de tiempo se admite modificar el agente antibiótico inicial por otro diferente (por simplificación del tratamiento empírico tras obtener los resultados del antibiograma, continuación de tratamiento intravenoso a tratamiento oral, modificación del agente por efectos adversos, etc.) siempre que el nuevo agente sea una opción terapéutica apropiada para el tratamiento de la infección en cuestión.
En caso de que el paciente no cumpla con los criterios clínicos necesarios para la retirada, deberá ser reevaluado en intervalos sucesivos de 48-72h hasta documentar que cumple estos criterios, procediéndose entonces a suspender el tratamiento.

Los pacientes que antes del momento de la aleatorización hubieran recibido un tratamiento activo pero a dosis no optimizadas según criterios de EUCAST, podrán ser incluidos en el ensayo. En este caso, la dosis será optimizada en el momento de la aleatorización, y no necesitarán prolongar la duración total del tratamiento prevista en el brazo del ensayo, siempre que cumplan los requisitos clínicos establecidos por el ensayo para la interrupción del tratamiento.

El tratamiento antibiótico podría reanudarse después de este punto si el médico a cargo del paciente lo considera necesario debido a una evolución desfavorable. La vía y la forma de administración del antibiótico, criterios para el alta hospitalaria o el manejo clínico de eventuales complicaciones se realizarán a criterio del médico responsable del paciente, siguiendo los estándares clínicos habituales y las políticas locales de manejo de antibióticos.

Se admite la continuación del tratamiento intravenoso mediante programas de terapia antimicrobiana domiciliaria endovenosa en pacientes que a criterio de su facultativo responsable reúnan criterios de estabilidad para ser manejados de manera ambulatoria.

Previamente al inicio del ensayo y durante su desarrollo, los centros participantes recibirán material didáctico sobre el uso óptimo de antibióticos en pacientes con infecciones invasivas producidas por *P. aeruginosa*, según la mejor evidencia disponible (Anexo V).

Hemocultivo de control: hemocultivo extraído en el momento de la inclusión del paciente, y realizado sucesivamente cada 48-72h si los hemocultivos siguen siendo positivos, hasta obtener un hemocultivo negativo. También serán extraídos hemocultivos de control siempre que el paciente presente síntomas o signos clínicos de una posible recidiva de la infección, o siempre que su situación clínica así lo requiera a criterio de su facultativo responsable.

5.6 Medicamentos y tratamientos permitidos

Se permite el uso de cualquiera de los antibióticos comúnmente empleados en la práctica clínica habitual para el tratamiento de la bacteriemia por *P. aeruginosa*, con las dosis y pautas de administración habituales descritas en la ficha técnica o que se recomienden por las sociedades científicas locales e internacionales en cada caso (**Tabla 1**).

No se establecen restricciones para el uso de otros medicamentos, sin embargo, solamente se recogerá como medicación concomitante la antibioterapia.

5.7 Seguimiento de los pacientes

Para los pacientes con criterios de ingreso hospitalario, el seguimiento clínico será realizado diariamente por su facultativo o facultativos responsables mientras permanezcan hospitalizados, y por el investigador Clínico responsable del Ensayo en el Centro y cuando se considere necesario por el Coordinador Clínico del Ensayo. Las visitas de aleatorización, fin de tratamiento y la visita 2 serán necesariamente presenciales. Para los pacientes sin criterios de ingreso hospitalario, las visitas 3 y 4 podrán realizarse mediante llamada telefónica en los días +30 desde la interrupción del tratamiento del ensayo y +90 desde la extracción del primer hemocultivo positivo, a través de un cuestionario estructurado (Anexo VI).
El seguimiento de los pacientes se realizará en las visitas definidas en este protocolo y se continuará hasta el día +90 desde la extracción del primer hemocultivo positivo. El calendario de visitas se especifica en la **Tabla 4** y en el apartado 8.4.

5.8 Criterios de finalización o interrupción del estudio

La interrupción prematura del ensayo clínico puede ocurrir por una decisión de las autoridades reguladoras, por un cambio en la opinión de los Comités Éticos de Investigación Clínica, por problemas de seguridad y/o del fármaco o indicios de ineficacia.

Se ha establecido una regla de interrupción predefinida para el análisis intermedio, que se realizará una vez reclutado el 40% del tamaño muestral. Éste será realizado por un comité independiente de evaluación que tendrá acceso completo a todos los datos del estudio (ver epígrafe 2.6.1). Se evaluará la posible futilidad del ensayo por medio de la potencia condicional (conditional power o CP) para la variable secundaria según la tendencia observada en el análisis intermedio, así como la proyectada si se cumple la hipótesis inicial. Se considera que un CP <15 % hace improbable demostrar la eficacia del tratamiento, por lo que se valorará conjuntamente la interrupción del ensayo si se atraviesa dicho margen. Además, cualquier diferencia en mortalidad entre los grupos será cuidadosamente evaluada.

El investigador, así como el promotor, se reservan el derecho a interrumpir el estudio en cualquier momento por motivos médicos y/o administrativos razonables.

5.9 Adquisición, acondicionamiento y etiquetado de la medicación

No procede ya que los medicamentos que se utilizarán en este estudio serán los que cotidianamente se dispensan en los Servicios de Farmacia de los hospitales participantes.

5.10 Almacenamiento y dispensación de la medicación

La medicación estará almacenada en el Servicio de Farmacia de cada centro bajo las condiciones de almacenamiento especificadas por el fabricante para dichos productos.

La dispensación se realizará según los procedimientos normalizados de cada centro participante, llevando siempre un control de administración. Se incluirá en la documentación del estudio un registro de dispensación para llevar la trazabilidad de los productos administrados, anotando el principio activo, lote, fecha de caducidad y número de unidades suministradas.

5.11 Final del ensayo

Se considerará final del ensayo el día de la visita final del último paciente incluido en el estudio.

6.- CRITERIOS DE SELECCIÓN

Pacientes adultos con bacteriemia monomicrobiana (aislamiento en hemocultivo) por *P. aeruginosa.*

6.1 Criterios de inclusión

1. Pacientes adultos (18 años o más).
2. Que presentan bacteriemia por *P. aeruginosa.*
3. Que hayan recibido de 6 días (+/-1) de tratamiento antibiótico activo frente a la bacteriemia contado desde la fecha de extracción del primer hemocultivo positivo y hasta el momento de la aleatorización.
4. Que hayan firmado el consentimiento informado del ensayo.

Nota: no está permitida la inclusión del mismo paciente más de una vez

6.2 Criterios de exclusión

1. Menores de 18 años.
2. Mujeres embarazadas o en período de lactancia. Las pacientes potencialmente fértiles deberán tener un test de gestación negativo.
3. Foco de la bacteriemia no adecuadamente controlado al menos 72h antes de la aleatorización.
4. Bacteriemia secundaria a una infección que requiera necesariamente un tratamiento antibiótico prolongado, mayor de 7 días, incluyendo:
    - neumonía post-obstructiva o necrosante
    - abscesos pulmonares
    - prostatitis aguda
    - infecciones de huesos y articulaciones
    - infecciones del sistema nervioso central
    - infecciones endovasculares relacionadas con prótesis vasculares.
    - Cualquier otra a juicio del médico responsable del paciente.
5. Coexistencia de una infección diferente en el momento del diagnóstico de la bacteriemia que también requiera tratamiento antibiótico.
6. Neumonía bacteriémica en pacientes gravemente inmunodeprimidos, definidos como:
    - pacientes con neutropenia grave (<500 células / mm3)
    - receptores de trasplante alogénico de células madre hematopoyéticas o de órganos sólidos, durante el primer año tras el trasplante
    - enfermedad activa de injerto contra huésped que requiera tratamiento inmunosupresor

- pacientes con tumores sólidos que estén recibiendo quimioterapia
- infección por VIH no tratada con CD4 < 200 células / mm3
- pacientes con inmunodeficiencia combinada primaria
- tratamiento con esteroides con prednisona > 20 mg / día (o equivalente) durante los 14 días previos a la aleatorización

7. Bacteriemia de cualquier origen en pacientes con neutropenia grave (<500 células/mm3) en el momento de la aleatorización.
8. Bacteriemia de cualquier origen en grandes quemados.
9. Bacteriemia producida por cepas resistentes a todos los betalactámicos y a quinolonas.
10. Bacteriemia polimicrobiana incluyendo microorganismos diferentes a *P. aeruginosa.*
11. Pacientes en cuidados paliativos o con una expectativa de supervivencia inferior a 48h en el momento de la aleatorización.
12. Bacteriemia por *P. aeruginosa* en los 90 días previos.
13. El médico responsable del paciente no quiere incluir al paciente en el ensayo clínico.

6.3 Criterios de retirada

6.3.1 Por decisión del paciente

De acuerdo con la Declaración de Helsinki, los pacientes tienen derecho a retirarse del estudio en cualquier momento y por cualquier motivo, pudiéndose expresar personalmente o a través de su representante.

6.3.2 Por pérdida de seguimiento

Debido a pérdida de seguimiento del paciente, se considerará pérdida de seguimiento cuando el paciente no realice las visitas de fin de tratamiento (Día 7+/-2 tras el inicio de tratamiento antibiótico activo en el brazo experimental y Día 14+/-2 tras el inicio de tratamiento antibiótico activo en el brazo control) y la visita de la prueba de curación (día +30 desde la interrupción del tratamiento del ensayo). Independientemente de ello, los datos de estos pacientes serán incluidos en el análisis por intención de tratar.

6.3.3 Por criterios de seguridad

Por la aparición de un acontecimiento adverso o cualquier otro motivo que, a criterio del clínico, obligue a la retirada del antibiótico en uso por no considerarse el tratamiento seguro para el paciente, pueda poner en peligro su vida o tener graves consecuencias para el mismo.

6.3.4 Por incumplimiento o violación de las normas contenidas en el protocolo

Cuando el paciente deje de cumplir las normas del ensayo podrá ser retirado a criterio del investigador responsable o por pérdida de seguimiento.

6.4 Seguimiento de los pacientes retirados prematuramente

Si se retira prematuramente a un paciente del ensayo, el investigador proporcionará la razón principal de la suspensión y, como indican las normas de BPC, se seguirán los procedimientos según protocolos habituales de tratamiento de su patología a criterio del clínico responsable.

6.5 Definiciones

Tratamiento activo: Cualquier tratamiento con actividad probada *in vitr*o contra la cepa responsable de la bacteriemia del paciente según criterios de EUCAST (34), independientemente de la dosis utilizada.
De manera específica, la monoterapia con aminoglucósidos no será considerada activa excepto para el tratamiento de la pielonefritis aguda.
El listado de tratamientos activos aparece reflejado en la **Tabla 1**.

Tratamiento optimizado: Tratamiento con actividad probada *in vitro* y administrado a dosis optimizadas según recomendaciones de EUCAST (34).
El listado de tratamientos optimizados está reflejado en la **Tabla 2.**

Los pacientes que antes del momento de la aleatorización hubieran recibido un tratamiento activo pero a dosis no optimizadas según criterios de EUCAST, podrán ser incluidos en el ensayo siempre que reúnan los criterios de mejoría clínica previstos para la aleatorización. En este caso, la dosis será optimizada en el momento de la aleatorización, no siendo necesario prolongar el tiempo de tratamiento previsto en su grupo por este motivo, siempre que cumplan los requisitos clínicos de seguridad establecidos por el ensayo para la interrupción del tratamiento.

Muerte: Muerte del paciente por cualquier causa.

Recurrencia probada: Comprende tres situaciones diferentes:

a) Recaída de los síntomas y signos de la infección responsable de la inclusión en el ensayo tras la interrupción del tratamiento, en un paciente cuyos síntomas ya se habían resuelto, con un nuevo aislamiento de *P. aeruginosa* en hemocultivos o en muestras representativas del lugar de la infección.

b) Pacientes con aislamientos recurrentes de *P. aeruginosa* en muestras microbiológicas sin signos ni síntomas clínicos de infección de nueva aparición están específicamente excluidos de esta categoría, ya que estos aislamientos deben considerarse como colonizaciones.

c) Paciente que desarrolla durante el seguimiento una infección hematógena a distancia atribuida a la infección que motivó la inclusión en el ensayo (espondilodiscitis, infección osteoarticular o de material protésico, embolismos pulmonares, etc.), en los que se documenta la presencia de *P. aeruginosa* en una muestra representativa del nuevo sitio de infección a distancia.

Recurrencia probable: Comprende tres situaciones diferentes:

a) Pacientes con recaída de los síntomas y signos locales de la infección que motivó la inclusión en el ensayo tras la interrupción del tratamiento, en un paciente cuyos síntomas ya se habían resuelto, pero en los que no se aísla ningún agente etiológico responsable de los síntomas (por ser los cultivos negativos o no haberse recogido).
Los pacientes con recurrencia de los síntomas y signos relacionados con el sitio inicial de la infección, para los que se determina en los cultivos una etiología diferente a *P. aeruginosa* están específicamente excluidos de esta categoría (deben codificarse como sobreinfecciones).

b) Pacientes con reaparición de la bacteriemia por *P. aeruginosa* sin ningún síntoma o signo focalizador en los que no se demuestra un foco para el nuevo episodio de bacteriemia.Los pacientes que tienen un nuevo episodio de bacteriemia por *P. aeruginosa* en los que sí se demuestra un foco alternativo y distinto al que motivó su inclusión en el ensayo están específicamente excluidos de esta categoría (deben codificarse como “nuevo episodio de bacteriemia”).

c) Paciente que desarrolla durante el seguimiento una infección hematógena a distancia atribuida a la infección que motivó la inclusión en el ensayo (espondilodiscitis, infección osteoarticular o de material protésico, embolismos pulmonares, etc.), en los que no se documenta la presencia de *P. aeruginosa* por no disponerse de ninguna muestra representativa del nuevo sitio de infección a distancia, o por no disponerse de muestras de este sitio de infección.
Los pacientes en los que se sospeche este tipo de complicaciones, pero en los que las muestras microbiológicas del nuevo sitio de infección confirmen una etiología alternativa para la infección diferente a *P. aeruginosa* están específicamente excluidos de esta categoría (deben codificarse como sobreinfecciones).

Al ser una variable de resultado subjetiva, la evaluación de la misma la realizará el comité externo de evaluación, que desconoce la asignación del caso al grupo experimental o control.

Recurrencia posible: Recaída de la fiebre tras la interrupción del tratamiento en un paciente cuyos síntomas ya se habían resuelto, sin síntomas específicos del sitio de la infección responsable de la inclusión en el ensayo, y sin ninguna otra causa alternativa definida para la fiebre.

La adscripción de los pacientes a las categorías de recurrencia probada, probable o posible, se facilita en el algoritmo del Anexo VII.

Fracaso del tratamiento: Muerte por cualquier causa o recurrencia probada o probable durante el seguimiento.

Curación clínica: Paciente vivo, con resolución de la fiebre y de los signos y síntomas de la infección responsable de la inclusión en el ensayo y hemocultivos de control negativos durante el seguimiento.

Control del foco: La realización de intervenciones destinadas a eliminar o drenar la fuente responsable de la bacteriemia, incluida la extracción de catéteres vasculares, derivaciones de vías urinarias o biliares, desbridamiento quirúrgico de tejidos blandos, drenaje quirúrgico o percutáneo de abscesos profundos, etc. En el caso de infecciones urinarias en pacientes con catéteres urinarios permanentes, la extracción del catéter no es obligatoria para considerar la fuente correctamente controlada.

Sobreinfección: Infecciones por cualquier otra etiología diferente a *P. aeruginosa*, incluidos otros bacilos Gram-negativos, *Enterococcus* sp., *Staphylococcus* sp., infección por *C. difficile* e infecciones fúngicas por levaduras u hongos filamentosos, durante el seguimiento.

Específicamente, los cultivos microbiológicos obtenidos en zonas no estériles (orina, piel, vía respiratoria) que no se correlacionen con un síndrome clínico de infección, serán interpretados como colonizaciones y no como sobreinfecciones.

Nuevo episodio de bacteriemia

Nuevo aislamiento de *P. aeruginosa* en hemocultivos por cualquier causa tras la interrupción del tratamiento en un paciente en el que ya se había demostrado un hemocultivo negativo tras el episodio inicial que motivó su inclusión en el ensayo.

Reaparición de fiebre

Reaparición de la fiebre una vez alcanzada la apirexia (temperatura ≤37ºC) desde hace al menos 72h.

Días libres de tratamiento antibiótico

Número total de días libres de tratamiento antibiótico. Para ello se sustraerán al total de días observados en cada paciente los días en los que se reciba al menos una dosis de tratamiento antimicrobiano (antibiótico o antifúngico) sea cual fuere su indicación (incluyendo la profilaxis antibiótica).

Días de hospitalización

Días desde el inicio del tratamiento hasta el alta hospitalaria. Se sumarán todos los días de estancia hospitalaria observados durante el seguimiento independientemente de si son consecutivos o no.

Acontecimientos adversos graves

Se recogerán el número de acontecimientos adversos graves y se presentarán como tasa por 1.000 pacientes-día y como proporción de pacientes con al menos un acontecimiento adverso grave.
Para la definición de acontecimientos adversos grave consultar la última versión del protocolo.

Recuperación de la capacidad funcional basal: Retorno a la actividad autorreferida que el paciente era capaz de realizar antes del episodio de bacteriemia.

***P. aeruginosa* multirresistente:** De acuerdo con la reciente propuesta del Centro Europeo para la Prevención y el Control de las Enfermedades Control de Enfermedades del Centro Europeo de Prevención y Control de Enfermedades, definimos un patógeno multirresistente como aquel que es resistente a al menos un agente de ≥3 clases de agentes antimicrobianos (35).

***P. aeruginosa* extremadamente resistente:** De acuerdo con la reciente propuesta del Centro Europeo para la Prevención y el Control de las Enfermedades Control de Enfermedades del Centro Europeo de Prevención y Control de Enfermedades, definimos un patógeno multirresistente como aquel que es resistente a algún agente de todas las clases de agentes antimicrobianos excepto uno o dos (35).

7.- TRATAMIENTO DE LOS SUJETOS

7.1 Medicación concomitante

Se permite el uso de cualquiera de los antibióticos comúnmente empleados en la práctica clínica habitual para el tratamiento de la bacteriemia por *P. aeruginosa*, con las dosis y pautas de administración habituales descritas en la ficha técnica o que se recomienden por las sociedades científicas locales e internacionales en cada caso (**Tabla 1**).

No se establecen restricciones para el uso de otros medicamentos, sin embargo solamente se recogerá en el CRD como medicación concomitante la antibioterapia.

7.2 Medicación de rescate

No está prevista la utilización de medicación de rescate. Si un paciente fuera retirado por falta de eficacia o alguna condición similar, será tratado según las guías clínicas y la práctica clínica habitual para estos casos.

7.3 Calendario de visitas y evaluaciones

Tabla 4. Calendario de visitas y procedimientos.

**Procedimientos**	**Visita 0 selección y aleatorización**	**Visita 1 Fin de tratamiento**	**Visita 2 Seguimiento**	**Visita 3 Test de curación**	**Visita 4 Fin de Seguimiento**	**Visita no planificada**
**Días**	**Día 0** (Día 6º +/-1) de tratamiento antibiótico activo)	**Día +7 (+/-1) o +14 (+/-1)** desde inicio tratamiento activo	**Día +7 +/-48h** desde fin tratamiento activo	**(Día +30 +/-2)** desde interrupción tratamiento del ensayo Telefónica*	**Día +90 +/-2)** desde el primer hemocultivo positivo Telefónica*	
**Aleatorización**	X					
**Consentimiento informado**	X					
**Criterios inclusión/exclusión**	X					
**Test de gestación**[1]	X					
**Historia clínica / anamnesis**	X	X	X	X	X	X
**Datos demográficos**	X					
**Exploración física y constantes vitales**	X	X	X			X
**Síntomas de infección**	X	X	X	X	X	X
**Tratamiento antibiótico**	X	X				X
**Hematología/ bioquímica**	X	X				X
**Hemocultivo**	X[3]					X
**Datos microbiológicos**[2]	X	X	X	X	X	X
**Muestras fecales**	X	X		X	X	
**Suspensión tratamiento antibiótico**		X				
**Medicación concomitante**	X	X	X	X	X	X
**Acontecimientos adversos**		X	X	X	X	X

1 Si procede. Se realizará en sangre u orina
2 Revisión de hemocultivos y cultivos para identificar aislamiento de *P. aeruginosa* y potenciales sobreinfecciones
3 En caso de que el primer hemocultivo de control fuera positivo se extraerá un nuevo hemocultivo en el momento de la recepción del informe de positividad. En caso de que este primer hemocultivo sea negativo, no serán necesarias ulteriores determinaciones salvo criterio clínico.

*Si el paciente participa en el estudio de diversidad bacteriana en la microbiota intestinal, la visita tendrá lugar de manera presencial

7.4 Procedimientos por visita

Visita 0 (Selección y aleatorización)

Se realizará **en el 6º día (+/-1) de tratamiento antibiótico activo** y se llevarán los siguientes procedimientos consecutivos.

1. Comprobación de los criterios de inclusión/exclusión. Los fallos en la selección se definen como aquellos sujetos que resultan candidatos del ensayo pero tras verificar criterios de inclusión/exclusión incumplen alguno de ellos. Todo ello deberá quedar registrado en una base de datos independiente al CRD.
2. Firma del consentimiento informado. Se explicará al paciente con detalle la naturaleza del estudio así como los riesgos y beneficios potenciales asociados al ensayo. Los pacientes firmarán el consentimiento informado (Anexo VIII) o en su defecto del representante legal.
3. Recogida de datos:
    - Datos demográficos: fecha de nacimiento, edad, género, fecha de inclusión.
    - Historia clínica, incluyendo antecedentes personales: alergias a fármacos, comorbilidades previas, tratamientos antibióticos administrados, motivo actual del ingreso, diagnóstico etiológico de la infección y posibilidades de controlar o erradicar el foco en las siguientes 24 horas cuando esto sea posible, síntomas basales y fecha de inicio de los mismos, tratamiento antibiótico recibido durante su hospitalización, índice de Charlson (Anexo IX) y Pitt score (Anexo X). Esta información se describe en detalle en el cuaderno de recogida de datos.
    - Exploración física incluyendo constantes vitales (presión arterial, frecuencia cardiaca, frecuencia respiratoria, temperatura corporal, saturación de oxígeno) y exploración por órganos y aparatos (auscultación cardiorrespiratoria, palpación abdominal, exploración de miembros y piel, incluyendo inspección de catéteres vasculares, urinarios o biliares si los hubiere).
    - Revisión de medicación concomitante.
4. Determinaciones de laboratorio:
    - Recogida de datos analíticos basales. Podrán recuperarse los datos analíticos obtenidos en las determinaciones sanguíneas pertinentes al proceso infeccioso del paciente que motiva su ingreso o asistencia en consultas. Se admite una variabilidad máxima de +/- 48 horas de la extracción sanguínea respecto a la fecha de la visita, debiendo recogerse los datos de la determinación más próxima a ésta. Los datos recogidos incluyen una bioquímica básica plasmática (glucosa, creatinina, urea, sodio, potasio, bioquímica hepática), proteína C reactiva y un hemograma básico.
    - En el caso de que la paciente a incluir fuera una mujer en edad fértil, se realizará en esta visita un test de gestación.

5. Extracción de hemocultivo en el momento de la aleatorización. Si éste fuera positivo se repetirá, si es negativo, no serán necesarias ulteriores determinaciones salvo mejor criterio clínico.

Visita 1 (Fin de tratamiento)

Brazo experimental: (Día 7+/-1 tras el inicio de tratamiento antibiótico activo).

1. Recogida de datos:
   - Historia clínica, incluyendo la presencia de signos o síntomas de infección y la fecha de resolución de los mismos, confirmación del adecuado control del foco de la infección, tratamientos antibióticos administrados, etc. Esta información se describe en detalle en el cuaderno de recogida de datos.
   - Exploración física incluyendo constantes vitales (presión arterial, frecuencia cardiaca, frecuencia respiratoria, temperatura corporal, saturación de oxígeno) y exploración por órganos y aparatos (auscultación cardiorrespiratoria, palpación abdominal, exploración de miembros y piel, incluyendo inspección de catéteres vasculares, urinarios o biliares si los hubiere).
   - Recogida de acontecimientos adversos, incluyendo reacciones adversas a fármacos, aparición de sobreinfecciones por microorganismos resistentes, o diarrea por *Clostridioides difficile*, tal y como se describe en el cuaderno de recogida de datos.
   - Recogida de medicación concomitante.
2. Determinaciones de laboratorio:
   - Recogida de datos analíticos.
3. Suspensión del tratamiento antibiótico si el paciente pertenece al grupo experimental. Previamente a suspender el tratamiento antibiótico deberá confirmarse que el paciente reúne todos los requisitos necesarios:
   - Apirexia (temperatura ≤37ºC) desde hace al menos 72h.
   - Resolución de los síntomas y signos atribuibles a la infección desde hace al menos 72h.

   En caso de que el paciente cumpla estos requisitos, se procederá a la suspensión del tratamiento antibiótico en los pacientes del grupo experimental.

   En caso de que el paciente no cumpla alguno de estos requisitos, se reevaluará nuevamente al paciente pasadas 48-72 h, repitiéndose la evaluación sistemática de estos dos criterios, y así sucesivamente.

En los pacientes asignados al brazo experimental, los procedimientos designados para la visita 0 y 1 pueden realizarse en un mismo día, unificándolos en una visita única.

Brazo control (Día 14 +/- 1 día tras el inicio de tratamiento antibiótico activo).

1. Recogida de datos:
    - Historia clínica, incluyendo la presencia de signos o síntomas de infección y la fecha de resolución de los mismos, confirmación del adecuado control del foco de la infección, tratamientos antibióticos administrados, etc. Esta información se describe en detalle en el cuaderno de recogida de datos.
    - Exploración física incluyendo constantes vitales (presión arterial, frecuencia cardiaca, frecuencia respiratoria, temperatura corporal, saturación de oxígeno) y exploración por órganos y aparatos (auscultación cardiorrespiratoria, palpación abdominal, exploración de miembros y piel, incluyendo inspección de catéteres vasculares, urinarios o biliares si los hubiere).
    - Recogida de acontecimientos adversos.
    - Recogida de medicación concomitante.
2. Determinaciones de laboratorio:
    - Recogida de datos analíticos.

3. Suspensión del tratamiento antibiótico si el paciente pertenece al grupo control. Previamente a suspender el tratamiento antibiótico deberá confirmarse que el paciente reúne todos los requisitos necesarios:
    - Apirexia (temperatura ≤37ºC) desde hace al menos 72h.
    - Resolución de los síntomas y signos atribuibles a la infección desde hace al menos 72h.

    En el caso de que el paciente cumpla estos requisitos, se procederá a la suspensión del tratamiento antibiótico en los pacientes del grupo experimental.

    En el caso de que el paciente no cumpla alguno de estos requisitos, se reevaluará nuevamente al paciente pasadas 48-72h, repitiéndose la evaluación sistemática de estos tres criterios, y así sucesivamente.

Visita 2 (Seguimiento, día +7 +/- 48h desde la fecha de interrupción del tratamiento).

1. Recogida de datos:
    - Historia clínica, incluyendo la presencia de signos o síntomas de infección y la fecha de resolución de los mismos, confirmación del adecuado control del foco de la infección, tratamientos antibióticos administrados, etc. Esta información se describe en detalle en el cuaderno de recogida de datos.
    - Exploración física incluyendo constantes vitales (presión arterial, frecuencia

cardiaca, frecuencia respiratoria, temperatura corporal, saturación de oxígeno) y exploración por órganos y aparatos (auscultación cardiorrespiratoria, palpación abdominal, exploración de miembros y piel, incluyendo inspección de catéteres vasculares, urinarios o biliares si los hubiere).
- Recogida de acontecimientos adversos.
- Recogida de medicación concomitante.
- Recogida de datos microbiológicos (revisión de hemocultivos y cultivos para identificar aislamiento de *P. aeruginosa* o sobreinfecciones)

Visita 3 (prueba de curación, día 30 +/-48h desde la interrupción del tratamiento antibiótico): se realizará por vía telefónica mediante un cuestionario estructurado (Anexo VI)**.**

1. Recogida de datos:
    - Recogida de datos clínicos, incluyendo la presencia de signos o síntomas de infección, tratamientos antibióticos administrados, etc. como se detalla en el cuaderno de recogida de datos.
    - Recogida de acontecimientos adversos.
    - Recogida de datos microbiológicos (revisión de hemocultivos y cultivos para identificar aislamiento de *P. aeruginosa* o sobreinfecciones).

Visita 4 (fin de seguimiento, día 90 +/48h desde <u>la fecha de extracción del primer hemocultivo positivo</u>): se realizará por vía telefónica mediante un cuestionario estructurado (Anexo VI)**.**

2. Recogida de datos:
    - Recogida de datos clínicos, incluyendo la presencia de signos o síntomas de infección, tratamientos antibióticos administrados, etc. como se detalla en el cuaderno de recogida de datos.
    - Recogida de acontecimientos adversos.
    - Recogida de datos microbiológicos (revisión de hemocultivos y cultivos para identificar aislamiento de *P. aeruginosa*)

7.5 Otros procedimientos del estudio (Subestudios)

7.5.1 Estudios microbiológicos

El procesamiento de los hemocultivos positivos se realizará en base a los procedimientos establecidos en cada centro participante. La identificación de los aislamientos de *P. aeruginosa* se realizará preferentemente mediante espectrometría de masas, considerándose segura la identificación a nivel de género y especie cuando el *score value* sea ≥ 2. El estudio de sensibilidad antibiótica se realizará mediante sistemas que permitan obtener el valor de CMI a los distintos antibióticos (microdilución comercial o tiras de gradiente de CMI) y deberán ser interpretados en base a criterios EUCAST (*The European Committee on Antimicrobial Susceptibility Testing. Breakpoint tables for interpretation of MICs and zone diameters*. Version 11.0, 2021).

Todos los aislados de *P. aeruginosa* que se recuperen de los hemocultivos (o de otras muestras clínica de interés) se conservarán congelados (preferentemente a -80ºC) para su envío al laboratorio 208 del Instituto de Biomedicina de Sevilla IBiS (Anexo XI). En él se registrarán y se enviarán al centro correspondiente de acuerdo con cada uno de los objetivos de los diferentes subestudios.

Las normas para toma de muestras y el procesamiento de las mismas se detallan en el Anexo XI. Los aislados se remitirán acompañados del formulario incluido en el Anexo XII.

7.5.2 Estudios de diversidad bacteriana en la microbiota intestinal.

La diversidad de la microbiota intestinal bacteriana de pacientes con bacteriemia por *P. aeruginosa* basal y en diferentes puntos de tiempo se recogerán en hospitales seleccionados tanto del brazo experimental, (grupo 1, 7 días de tratamiento) o del brazo de control, (grupo 2, 14 días de tratamiento). Las muestras de heces de pacientes de cada grupo 1 y 2 se recolectarán en la visita 0 (aleatorización), visita 1 (fin de tratamiento), visita 3 (prueba de curación) y visita 4 (fin de seguimiento). Para cada grupo, se obtendrán 20 muestras de pacientes que reciben tratamiento con betalactámicos con espectro de actividad para bacilos anaeróbicos (piperacilina / tazobactam y carbapenémicos), de 20 muestras de pacientes que reciben tratamiento con betalactámicos sin actividad frente a anaerobios. (ceftazidima y cefepime) y 20 muestras de pacientes que reciben ciprofloxacino.

8.- VALORACIÓN DE LA EFICACIA

8.1 Variables de eficacia

Principal: Análisis DOOR/RADAR en el día +30 desde la interrupción del tratamiento del ensayo.

Secundarias:
Variable secundaria de no-inferioridad: fracaso del tratamiento en el día +30.

Otras variables secundarias: fracaso del tratamiento en el día +90, mortalidad por todas las causas, curación clínica, análisis DOOR, sobreinfecciones, días de estancia hospitalaria y recurrencias (probadas, probables o posibles), reaparición de la fiebre, nuevo episodio de bacteriemia, recuperación de capacidad funcional basal en los días +30 y +90. Número total de días libres de tratamiento antibiótico y días de hospitalización en el día +90.

8.2 Pruebas de laboratorio

Se contempla realizar análisis sanguíneos en la visita inicial y posteriormente según lo especificado en el calendario de visitas (**Tabla 4**). Dichos análisis serán realizados de manera local en cada centro atendiendo a los estándares de práctica clínica habitual. Se realizarán las siguientes pruebas:

- Hemograma que incluya recuento de leucocitos, neutrófilos, hemoglobina y plaquetas.

- Bioquímica sanguínea que incluya la determinación de sodio, potasio, creatinina, urea, proteína C reactiva, AST (GOT), ALT (GPT) y bilirrubina total (si alterada también bilirrubina directa).

9.- VALORACIÓN DE SEGURIDAD

9.1 Evaluaciones de seguridad

Se realizarán las siguientes evaluaciones clínicas para evaluar el perfil de seguridad del Ensayo:

9.1.1 Exploración física, constantes vitales

Exploración física y constantes vitales. Se realizará en cada una de las visitas una toma de constantes vitales (presión arterial, frecuencia cardiaca, frecuencia respiratoria, temperatura corporal, saturación de oxígeno) y exploración por órganos y aparatos (auscultación cardiorrespiratoria, palpación abdominal, exploración de miembros y piel, incluyendo inspección de catéteres vasculares, urinarios o biliares si los hubiere).

9.1.2 Pruebas de laboratorio y microbiológicas

Se realizarán hemograma básico, bioquímica y hemocultivos seriados según calendario de procedimientos. Igualmente se realizarán estudios microbiológicos de identificación de las cepas patógenas.

9.2 Acontecimientos adversos de interés para su seguimiento

Incidencia de acontecimientos adversos.

- Diarrea: a aquellos sujetos que experimenten diarrea (3 o más deposiciones al día de consistencia disminuida) durante el estudio se les solicitará la detección de toxina de *C. difficile* en heces. Tanto si es positiva como negativa, se registrará como acontecimiento adverso (AA) aunque no obligará a suspender el tratamiento del estudio, salvo que así lo considere el IP.
- Náuseas, vómitos.
- Cefalea.
- Convulsiones.
- Toxicidad hepática (elevación de transaminasas y/o bilirrubina).
- Anemia hemolítica y otras alteraciones hematológicas (leucopenia, trombopenia).
- Alteraciones de la coagulación.
- Alteraciones en los niveles de sodio y potasio.
- Reacciones de hipersensibilidad y exantema cutáneo.

9.3 Definiciones

Acontecimiento Adverso (AA):

Un acontecimiento adverso es cualquier reacción médica no deseada experimentada por el paciente en cualquier momento en el transcurso del estudio, se considere o no relacionada con el tratamiento del estudio. Esta definición incluye la aparición de una nueva enfermedad y la exacerbación de trastornos preexistentes distintos de la indicación en estudio.

Reacción Adversa (RA):

Una RA es toda reacción nociva y no intencionada a un medicamento en investigación, independientemente de la dosis administrada.

Acontecimiento Adverso Grave (AAG) y Reacción Adversa Grave (RAG):

Se consideran graves los AA o RA que, a cualquier dosis puedan provocar la muerte, amenazar la vida del sujeto, requerir la hospitalización del paciente o prolongar una hospitalización existente, provocar invalidez o incapacidad permanente o importante, o dar lugar a una anomalía o malformación congénita. También se consideran graves aquellas sospechas de AA o RA importantes desde el punto de vista médico, aunque no cumplan los criterios anteriores, incluyendo los acontecimientos médicos importantes que requieran una intervención para evitar que se produzca una de las consecuencias anteriormente descritas. Asimismo, se notificarán como graves todas las sospechas de transmisión de un agente infeccioso a través de un medicamento.

El concepto “amenazar la vida del sujeto” en la definición se refiere a que, en opinión del investigador, el paciente en el momento del AA o RA está en riesgo real de muerte; no se refiere a que el AA/RA hipotéticamente pudiera haber ocasionado la muerte en caso de haber sido más intenso.

El concepto “requerir hospitalización” excluirá tanto las hospitalizaciones planeadas para tratamientos programados como las que hayan sido planificadas o que se prevean antes de comenzar el estudio en relación con una situación médica preexistente.

Los siguientes acontecimientos adversos, se recogerán en el CRD del estudio y serán evaluados dentro de las variables de seguridad, pero debido a su esperabilidad dentro de la evolución propia de la patología en estudio **no se realizará notificación específica al departamento de FV (no será necesario cumplimentar el formulario de SAES):**

- Recidiva probada o probable que cumpla criterios de acontecimiento adverso grave.
- Sobreinfección que cumpla criterios de acontecimiento adverso grave.

Ver definiciones en apartado 7.2

Reacción Adversa Inesperada (RAI):

Cualquier RA cuya naturaleza, intensidad o consecuencias no se corresponde con la información de seguridad de referencia.

Reacción Adversa Grave e Inesperada (RAGI):

RAG (definida previamente), cuya naturaleza, gravedad o consecuencias no se corresponde con la información de seguridad de referencia.

Criterios de Causalidad:

- AA relacionado: La relación temporal del AA con la medicación en estudio indica una relación causal posible y no puede ser explicado por factores tales como el estado clínico del paciente, intervenciones terapéuticas.
- AA no relacionado: La relación temporal del AA con la medicación del estudio indica una relación causal improbable, o bien otros factores (medicación o condiciones concomitantes), otras intervenciones terapéuticas proporcionan una explicación satisfactoria para el AA.

9.4 Información de seguridad de referencia

Fichas técnicas de todos los antibióticos con actividad antipseudomonas con indicación autorizada, se incluirán en el archivo del investigador de cada centro participante.

9.5 Notificación y recogida de acontecimientos adversos graves

El investigador principal o un colaborador deberá comunicar al departamento de Farmacovigilancia FV-UICEC-HUVR, todos los acontecimientos adversos graves (tal como se definen a continuación), se consideren o no relacionados con el tratamiento o esperados, en un plazo de 24 horas (un día laborable), a partir de su conocimiento (Anexo XIII). Deben notificarse los acontecimientos adversos graves que se produzcan en cualquier momento desde la inclusión del paciente en el estudio (que se define como el momento en que el sujeto firma el consentimiento informado) y hasta 90 días desde la fecha de extracción del primer hemocultivo positivo o abandone el estudio. A un sujeto se le considera completo BIEN tras la conclusión de la última visita o contacto (p.ej., contacto telefónico con el investigador o un colaborador), como se indica en el calendario de evaluaciones del protocolo, O BIEN tras la última dosis de la medicación del estudio, lo que suceda más tarde. La retirada se define como la fecha en que un sujeto y/o el investigador determinan que el sujeto no puede ya cumplir los requisitos del estudio en ninguna de las visitas y evaluaciones subsiguientes.

El investigador cumplimentará y firmará el formulario de notificación de AAG (Anexo XIII) que enviará por fax o correo electrónico a:

Unidad de Investigación Clínica y Ensayos Clínicos
Hospital Universitario Virgen del Rocío

Dpto. Farmacovigilancia
email: pv_shorten2@scren.es
Avda. Manuel Siurot S/N
41013. Sevilla
Tel.: 955 01 34 14
Fax: 955095338

El personal de FV revisará el formulario recibido y, si procede, solicitará información adicional al investigador. El investigador proporcionará información al promotor o a quien asuma las tareas delegadas por el promotor (Unidad de FV-UICEC-HUVR) siempre que se le solicite y, en cualquier caso, cuando cambie su evaluación inicial en cuanto a gravedad o causalidad. Para comunicar la información de seguimiento se seguirá el procedimiento de notificación descrito previamente.

El personal de FV-UICEC-HUVR llevará un registro detallado de todos los AAG o de especial interés que le sean comunicados por los investigadores.

En caso de que se produzca algún error de medicación o el medicamento en investigación se utilice fuera de lo previsto en el protocolo durante el desarrollo del estudio, el investigador lo notificará al FV-UICEC-HUVR en un plazo de 24 horas desde su conocimiento. El circuito para la notificación y el formulario serán los mismos que para los AAG.

Se considerarán graves aquellos AA que cumplan los criterios de gravedad descritos en el apartado de definiciones 10.1. Los acontecimientos clínicamente significativos que no produzcan la muerte ni constituyan amenaza para la vida o precisen hospitalización pueden considerarse experiencias farmacológicas adversas graves cuando, basándose en un juicio médico adecuado, puedan poner en peligro al sujeto o requerir una intervención médica o quirúrgica para prevenir uno de los resultados enumerados en esta definición. Ejemplos de tales acontecimientos médicos son el broncoespasmo alérgico que precise tratamiento intensivo, domiciliario o en una unidad de urgencias, discrasias sanguíneas o convulsiones que no den lugar a hospitalización, o desarrollo de una dependencia o de abuso de sustancias.

También es necesario notificar las alteraciones de las pruebas de laboratorio, a no ser que se indique lo contrario en esta sección del protocolo.

9.6 Notificación expeditiva de RAGI

El departamento de FV-UICEC-HUVR, es responsable de notificar a la AEMPS y a las CCAA donde se realiza el ensayo, todas las RAGI que se recojan en el estudio, siguiendo el procedimiento indicado en la legislación vigente.

El plazo máximo de notificación de un caso individual de sospecha de RAGI será de 15 días naturales a partir del momento en el que el promotor haya tenido conocimiento de la misma. Cuando la sospecha de RAGI haya ocasionado la muerte del paciente, o puesto en peligro su vida, el promotor enviará la información en un plazo de 7 días naturales a partir del momento en el que tenga conocimiento de la misma. Completará dicha información, en lo posible, en los 8 días siguientes.

Esta información deberá incluir una evaluación de la importancia e implicación de los hallazgos, incluyendo experiencia previa relevante con el mismo medicamento o similares. Asimismo, se deberá notificar al órgano competente de cada una de las Comunidades Autónomas donde se realiza el ensayo las sospechas de RAGI ocurridas en los centros sanitarios de su Comunidad. En ambos casos, se utilizará para ello el formulario de notificación de RAGI.

9.7 Notificación expeditiva de otra información de seguridad relevante

El departamento de FV-UICEC-HUVR notificará tan pronto como sea posible y no más tarde de 15 días después de que tenga conocimiento de ella cualquier información que pudiera modificar la relación beneficio/riesgo del medicamento en investigación (por ejemplo: aumento en el porcentaje de aparición de las RAG esperadas, las RAGI que ocurran después de la finalización de un ensayo clínico, nuevos acontecimientos relacionados con la realización del ensayo o del desarrollo del medicamento en investigación, cualquier recomendación del comité de monitorización de datos relevante para la seguridad de los sujetos, etc.).

9.8 Notificación a los investigadores

El promotor presentará a los investigadores la información de seguridad que podría impactar en la seguridad de los pacientes incluidos en el estudio tan pronto como sea posible.

Además, el investigador será informado a lo largo del estudio acerca de cualquier aspecto sobre la seguridad, incluyendo las modificaciones del protocolo debidas a razones de seguridad.

9.9 Errores de medicación

Los errores de medicación son errores no intencionados en la prescripción, dispensación, administración o monitorización de un medicamento mientras está bajo el control de un profesional de la salud o del paciente y que puede causar daño al paciente.

El mal uso se refiere a una situación en la que el producto médico se utiliza de manera intencional de forma inapropiada, sin estar en concordancia con el protocolo.

Los errores de medicación del estudio y el uso fuera de lo previsto en el protocolo se registran en el cuaderno de recogida de datos (CRD), independientemente de si están asociados con un AA/AAG o no. El mal uso o abuso se recopilará e informará en la base de datos de seguridad dentro de las 24 horas del conocimiento del mismo por parte del investigador.

10.- ESTADÍSTICA

10.1 Cálculo del tamaño muestral

Definición de superioridad. La superioridad del tratamiento experimental para la variable del resultado principal del estudio se establece si el tratamiento de 7 días es un ≥60% mejor que el tratamiento de 14 días, evaluado mediante la escala DOOR/RADAR.

Para el cálculo del tamaño muestral se realizó la prueba U de Mann-Whitney para una hipótesis nula de un 60% de probabilidad de que X > Y (siendo “X” la puntuación DOOR/RADAR de un paciente del grupo experimental seleccionado al azar, e “Y” la puntuación DOOR/RADAR de un paciente del grupo control seleccionado al azar), siguiendo la metodología previamente publicada.

Para confirmar esta hipótesis de superioridad el tamaño muestral necesario estimado será de 262 pacientes, asumiendo un error α = 0,05, una potencia del 80%.

La hipótesis de no inferioridad se evaluará para la variable fracaso del tratamiento evaluada a los 30 días tras la interrupción del tratamiento del ensayo. Un estudio observacional publicado por Bae et al. (20), reporta una tasa de recurrencia o muerte por cualquier causa en este punto del 15,6% y el 11,3% para los pacientes que reciben tratamientos prolongados o cortos. Considerando este resultado y eligiendo un margen de no inferioridad del 7,5%, una potencia del 80%, un error α unilateral de 0,025, el tamaño muestral estimado sería de 262.

El número final de la muestra se ha ajustado, esperando una no-adherencia al tratamiento del 5% y unas pérdidas de seguimiento del 5%, con lo que el tamaño muestral necesario estimado para analizar la hipótesis de no inferioridad será de **306 pacientes**. Con ello se estima una potencia suficiente para ambas variables.

10.2 Análisis estadístico

El análisis primario se realizará en la población por intención de tratar (ITT) (todos los pacientes aleatorizados) y en la población por protocolo (PP) (todos los pacientes que cumplen con el protocolo del estudio sin desviaciones importantes).

Los análisis estadísticos a realizar serán los siguientes:

1. Estudio descriptivo de todas las variables. Variables continuas que se informarán con número de observaciones, mediana y rango intercuartílico y variables categóricas como números absolutos y porcentajes.

2. Prueba de chi-cuadrado o prueba exacta de Fisher para comparar las variables cualitativas en los dos grupos de estudio.

3. T de Student o U de Mann-Whitney para variables cuantitativas, según su ajuste a la distribución normal.

4. Análisis multivariado usando regresión logística o regresión lineal según sea apropiado para las variables asociadas independientemente de las variables de resultado.

5. Se realizará un análisis de subgrupos predefinido para las bacteriemias de origen pulmonar y extrapulmonar, los pacientes que reciben tratamiento optimizado y los pacientes inmunodeprimidos.

6. Los datos faltantes se analizarán mediante imputación múltiple.

7. Análisis DOOR/RADAR: además se realizará un análisis mediante desirability of outcome ranking (DOOR) y response adjusted for duration of antibiotic risk (RADAR) con la clasificación indicada en el apartado 5.1.1.

Todos los análisis se realizarán con los programas estadísticos R versión 3.6.3 y SPSS versión 22.0.

Consideraciones especiales: un comité de expertos (apartado 10.6) para evaluar a los pacientes con fracaso del tratamiento, específicamente la recurrencia probada o probable, por ser la variable no objetiva de los resultados. Debido al criterio anterior se someterán a la evaluación final de un panel de investigadores del estudio. Este comité estará cegado para así desconocer la asignación del paciente evaluado al grupo experimental o al control. Se pedirá a los tres investigadores del Comité que confirmen o no la recurrencia de la bacteriemia, proporcionando en este caso una explicación alternativa. En caso de discrepancia, la decisión final se tomará por mayoría.

10.3 Poblaciones de análisis

Los análisis de los resultados primarios y secundarios se realizarán en las poblaciones por intención de tratar (ITT) y por protocolo (PP). Éstas se definen como sigue:

- Población por intención de tratar: Todos los pacientes aleatorizados constituyen esta población, tanto si los pacientes continuaron recibiendo la terapia antibiótica o no.
- Población por protocolo: Este grupo está formado por todos los pacientes que fueron aleatorizados, recibieron la duración de la terapia antibiótica asignada (dentro de ± 24 horas), tuvieron un seguimiento en el día +30 desde la interrupción del tratamiento antibiótico y en los que no se documentaron desviaciones mayores del protocolo durante el periodo de estudio.

10.4 Análisis de seguridad y otros resultados secundarios

La frecuencia y el grado de severidad de los EA que se consideren relacionados posiblemente, probablemente o definitivamente con el antibiótico del estudio se describirán por clase de órgano del sistema y por el término preferido del Diccionario Médico para Actividades Reguladoras (MedDRA). La frecuencia de los EA se comunicará y se comparará entre los grupos de entre los grupos de intervención con la prueba exacta de Fisher o la prueba de Chi-cuadrado, según corresponda.

10.5 Análisis intermedio

Se realizará un análisis intermedio cuando se reclute el 40% de la muestra y se evaluarán todos los resultados definidos para el análisis final.

Éste será realizado por un comité independiente de evaluación que tendrá acceso completo a todos los datos del estudio (ver epígrafe 2.6.1). Se evaluará la posible futilidad del ensayo por medio de la potencia condicional (conditional power o CP) para la variable secundaria según la tendencia observada en el análisis intermedio, así como la proyectada si se cumple le hipótesis inicial. Se considera que un CP <15 % hace improbable demostrar la eficacia del tratamiento, por lo que se valorará conjuntamente la interrupción del ensayo si se atraviesa dicho margen. Además, cualquier diferencia en mortalidad entre los grupos será cuidadosamente evaluada.

Estos datos serán evaluados por una junta independiente de monitorización de datos y seguridad compuesto por 3 investigadores expertos pertenecientes al CIBER de enfermedades infecciosas y no participantes en este estudio (DSMB). La composición de estos miembros será comunicada al Comité de Ética antes del inicio del ensayo.

Las principales responsabilidades del DSMB son

1) Revisar y evaluar periódicamente los datos acumulados del estudio para la seguridad de los participantes, la realización del estudio y el progreso, y cuando sea apropiado, eficacia.

2) Hacer recomendaciones al equipo de coordinación del estudio sobre la continuación, modificación o terminación del ensayo. Para este objetivo, una definición clara del calendario de reuniones, Las reglas de detención con descripciones estadísticas y miembros seleccionados del comité serán aprobadas antes del inicio del estudio.

10.6 Comité independiente de evaluación

Para evitar en la medida de lo posible el sesgo que el carácter abierto del ensayo pudiera generar, la evaluación de los resultados será realizada por un comité independiente ciego (que no forme parte del estudio como investigadores) respecto de la asignación del tratamiento. Dicho comité estará formado por tres investigadores expertos pertenecientes al CIBER de enfermedades infecciosas y tomará sus conclusiones por consenso.

11.- ASPECTOS ÉTICOS

El ensayo se llevará a cabo de acuerdo con los principios que emanan de la Declaración de Helsinki, y según la normativa legal vigente (Real Decreto 1090/2015), y no se iniciará hasta haber obtenido la aprobación del CEIC de referencia, la conformidad de los directores de las Instituciones, y la autorización de la Agencia Española de Medicamentos y Productos Sanitarios.

El investigador debe cumplir todos los requisitos del protocolo. Si se produce una situación en la que se requiera una desviación temporal del protocolo, el investigador u otro médico responsable del paciente debe ponerse en contacto con el monitor lo antes posible con objeto de comentar la situación y acordar una línea de acción adecuada. El investigador documentará la desviación respecto al protocolo y las circunstancias que la exigieron.

11.1 Consentimiento informado

El paciente debe otorgar su consentimiento antes de ser admitido en el estudio clínico. El médico habrá de explicar la naturaleza, propósitos y posibles consecuencias del ensayo clínico, de una manera comprensible al paciente. La información proporcionada por el médico deberá ser también registrada. En la obtención y documentación del mismo, los investigadores cumplirán la legislación pertinente (artículo 4 del Real Decreto 1090/2015), las normas de la buena práctica clínica y los principios éticos que tienen su origen en la Declaración de Helsinki.

El sujeto del estudio otorgará su consentimiento, firmando el modelo correspondiente. El investigador recibirá un número adecuado de modelos de consentimiento informado a

través del promotor. A este fin cada modelo debe llevar la firma del investigador y del paciente.

El investigador no iniciará ninguna investigación correspondiente al ensayo hasta que haya obtenido el consentimiento del paciente.

11.2 Protección de datos

El tratamiento, la comunicación y la cesión de los datos de carácter personal de todos los sujetos participantes se ajustará a lo dispuesto en el Reglamento (UE) 2016/679 del Parlamento Europeo y del consejo de 27 de abril de 2016 relativo a la protección de las personas físicas en lo que respecta al tratamiento de datos personales y a la libre circulación de estos datos y la Ley Orgánica 3/2018, de 5 de diciembre de Protección de Datos Personales y garantía de los derechos digitales. De acuerdo con lo que establece la legislación mencionada, el paciente podrá ejercer los derechos de acceso, modificación, oposición y cancelación de datos, para lo cual deberá dirigirse a su médico del estudio.

Se mantendrá en todo momento el anonimato de los sujetos participantes en el estudio. Así pues, los datos recogidos para el estudio estarán identificados mediante un código y sólo el investigador y colaboradores podrán relacionar dichos datos con el paciente y con su historia clínica. Por lo tanto, la identidad del paciente no será revelada a persona alguna salvo excepciones: personal autorizado por el promotor, cuando lo precisen, para comprobar los datos y procedimientos del estudio, pero siempre manteniendo la confidencialidad de los mismos de acuerdo a la legislación vigente; en caso de urgencia médica o requerimiento legal (autoridades sanitarias: Agencia Española de Medicamentos y Productos Sanitarios y Comité Local de Ensayos Clínicos).

Los datos de este estudio se utilizarán únicamente para los fines específicos del mismo.

11.3 Responsabilidades de los participantes en el estudio

El sujeto participante deberá seguir las indicaciones de los investigadores y comunicar cualquier eventualidad a los mismos. El sujeto será debidamente informado de las prohibiciones o restricciones a las que deberá atenerse durante la realización del ensayo.

El incumplimiento de estas recomendaciones implicará el abandono del estudio.

Todos los sujetos participantes en el estudio tienen derecho a abandonar el estudio en cualquier momento, retirando su consentimiento, sin tener que justificar esta decisión y sin que ello le suponga detrimento alguno en su seguimiento clínico. Si esto ocurre, el investigador intentará que el sujeto realice todas las evaluaciones necesarias para

asegurar que no se presentan acontecimientos adversos y para garantizar un seguimiento apropiado en el caso de que se haya presentado algún tipo de problema.

11.4 Monitorización y auditoría

El estudio será monitorizado mediante visitas locales, llamadas telefónicas e inspección periódica de los CRD con la frecuencia suficiente para comprobar lo siguiente:

- Ritmo de inclusión de pacientes, cumplimiento de las normas de los procedimientos del protocolo, integridad y exactitud de los datos introducidos en los cuadernos, verificación frente a los documentos originales y aparición de acontecimientos adversos.

- Las visitas de monitorización serán realizadas por los monitores del estudio. Se entiende que estos monitores podrán acceder a las historias clínicas de los pacientes después de solicitarlo el investigador. El investigador dedicará el tiempo suficiente a estas visitas y facilitará el acceso a toda la documentación a las personas autorizadas.

- El estudio podrá ser auditado por un organismo independiente. De igual manera, miembros del CEIC podrán realizar un seguimiento al mismo.

11.5 Finalización prematura o suspensión del estudio

Si el ensayo finaliza prematuramente o se suspende, el promotor debe informar rápidamente al investigador y a las autoridades reguladoras de la finalización o suspensión y de la razón para ello. El promotor o investigador debe informar rápidamente al CEIC y facilitarle la razón de la finalización o suspensión, tal y como especifiquen los requisitos reguladores pertinentes.

11.6 Documentación del estudio

La documentación relativa al estudio (protocolo, CRD, consentimiento informado, autorizaciones…) será archivada en un lugar seguro y fácilmente accesible por el equipo investigador. Toda la información contenida en informes clínicos, histológicos, bioquímicos, observaciones u otras actividades es necesaria para la reconstrucción y evaluación del estudio.

12.- FINANCIACIÓN Y SEGURO

12.1 Financiación

El proyecto ha recibido financiación por convocatoria pública de Investigación Clínica Independiente del Instituto de Salud Carlos III con número de expediente ICI21/00075.

12.2 Seguro

Se solicitará al comité ético la consideración de ensayo clínico de bajo nivel de intervención de acuerdo a su definición establecida en el RD 1090/2015. En caso de no ser aceptado el promotor contratará, de acuerdo con la legislación española, un seguro de responsabilidad civil, de acuerdo a la legislación vigente (RD 1090/2015, artículo 9). Esta póliza cubrirá todos los posibles daños y perjuicios que el sujeto pueda sufrir a consecuencia de la administración del producto en estudio. Dicha póliza se abonará y se hará efectiva antes del inicio del ensayo clínico, si este es aprobado por las autoridades sanitarias correspondientes.

13.- POLÍTICA DE PUBLICACIÓN

Éstas se ajustarán a lo dispuesto en el Real Decreto 1090/2015 de 4 de diciembre, por el que se regula los Ensayos Clínicos con medicamentos, los Comités de Ética de la Investigación con medicamentos y el Registro Español de Estudios Clínicos, artículo 42, que recoge el siguiente texto:

“1. El promotor está obligado a publicar los resultados, tanto positivos como negativos, de los ensayos clínicos autorizados, preferentemente, en revistas científicas antes de ser divulgados al público no sanitario, con independencia de las obligaciones de publicación del informe de los resultados en el Registro español de estudios clínicos (REec) y de lo establecido al respecto en el Reglamento (UE) n. º 536/2014 del Parlamento Europeo y del Consejo, de 16 de abril de 2014.

2. Cuando se hagan públicos estudios y trabajos de investigación sobre medicamentos, dirigidos a la comunidad científica, se harán constar los fondos obtenidos por el autor, por o para su realización, y la fuente de financiación.

3. Se mantendrá en todo momento el anonimato de los sujetos participantes en el ensayo.

4. No se darán a conocer de modo prematuro o sensacionalista tratamientos de eficacia todavía no determinada, ni se exagerará esta. No se dará publicidad a resultados intermedios que puedan comprometer la fiabilidad de los resultados finales del ensayo.

5. La publicidad de medicamentos de uso humano en investigación queda terminantemente prohibida, tal como se establece en el texto refundido de la Ley de garantías y uso racional de los medicamentos y productos sanitarios, en el Real Decreto 1416/1994, de 25 de junio, por el que se regula la publicidad de los medicamentos de uso humano, en el Real Decreto 1907/1996, de 2 de agosto, sobre publicidad y promoción comercial de productos, actividades o servicios con pretendida finalidad sanitaria, y en la Ley 34/1988, de 11 de noviembre, General de Publicidad.

6. En todos los casos, para hacer públicos los resultados generales de las investigaciones una vez concluidas, se seguirán las directrices de la Comisión Europea y, en su caso, las instrucciones de la Agencia Española de Medicamentos y Productos Sanitarios.

7. Cuando un subestudio de un ensayo clínico finalice en fecha posterior al resto del ensayo será necesario que el resumen de sus resultados se publique en el año siguiente a su finalización, sin que esto suponga retraso en la presentación de los resultados del resto del ensayo.”

ANEXO I. BIBLIOGRAFÍA

1. WHO | Global action plan on antimicrobial resistance. WHO. [citado 28 de enero de 2019]. http://apps.who.int/iris/bitstream/handle/10665/193736/9789241509763_eng.pdf?sequence=1

2. Informe anual 2019-2020 Plan Nacional frente a la Resistencia a los Antibióticos (PRAN). [citado 17 de marzo de 2021]. https://resistenciaantibioticos.es/es/publicaciones/informe-anual-2019-2020-plan-nacional-frente-laresistencia-los-antibioticos.

3.European Centre for Disease Prevention and Control (ECDC). Antimicrobial resistance strategies and action plans. 2016 https://www.ecdc.europa.eu/en/publications-data/directory-guidance-prevention-and-control/antimicrobial-resistance-strategies

4. Rodríguez-Baño J, et al. Programas de optimización de uso de antimicrobianos (PROA) en hospitales españoles: documento de consenso GEIH-SEIMC, SEFH y SEMPSPH. Enferm Infecc Microbiol Clin. 2012;30(1):22.e1-22.e23.

5. Molina J, et al. Long-Term Impact of an Educational Antimicrobial Stewardship Program on Hospital-Acquired Candidemia and Multidrug-Resistant Bloodstream Infections: A Quasi-Experimental Study of Interrupted Time-Series Analysis. Clin Infect Dis. 2017; 65(12):1992-9.

6. Peñalva G, et al. Long-term impact of an educational antimicrobial stewardship programme in primary care on infections caused by extended-spectrum β-lactamase-producing Escherichia coli in the community: an interrupted time-series analysis. Lancet Infect Dis. 2020; 20(2):199-207.

7. Uranga A, et al. Duration of Antibiotic Treatment in Community-Acquired Pneumonia: A Multicenter Randomized Clinical Trial. JAMA Intern Med. 2016; 176(9):1257-65.

8. Chastre J, et al. Comparison of 8 vs 15 days of antibiotic therapy for ventilator-associated pneumonia in adults: a randomized trial. JAMA. 2003; 290(19):2588-98.

9. Eliakim-Raz N, et al. Duration of antibiotic treatment for acute pyelonephritis and septic urinary tract infection-- 7 days or less versus longer treatment: systematic review and meta-analysis of randomized controlled trials. J Antimicrob Chemother. 2013; 68(10):2183-91.

10. Sawyer RG, et al. Trial of Short-Course Antimicrobial Therapy for Intraabdominal Infection. N Engl J Med. 2015; 372(21):1996-2005.

11. Aguilar-Guisado M, et al. Optimisation of empirical antimicrobial therapy in patients with haematological malignancies and febrile neutropenia (How Long study): an open-label, randomised, controlled phase 4 trial. Lancet Haematol. 2017; 4(12):e573-83.

12. Yahav D, Franceschini E, Koppel F, Turjeman A, Babich T, Bitterman R, et al. Seven Versus 14 Days of Antibiotic Therapy for Uncomplicated Gram-negative Bacteremia: A Noninferiority Randomized Controlled Trial. Clin Infect Dis 2019; 69(7):1091-8.

13. von Dach E, et al. Effect of C-Reactive Protein-Guided Antibiotic Treatment Duration, 7-Day Treatment, or 14-Day Treatment on 30-Day Clinical Failure Rate in Patients With Uncomplicated Gram-Negative Bacteremia: A Randomized Clinical Trial. JAMA. 2020; 323(21):2160-9.
14. Araos R and D'Agata E. Mandell, Douglas and Bennett's Principles and Practice of Infectious Diseases. Elsevier; 2020. p. 2686-99.
15. Peña C, et al. Influence of virulence genotype and resistance profile in the mortality of Pseudomonas aeruginosa bloodstream infections. Clin Infect Dis. 2015; 60(4):539-48.
16. Recio R, et al. Pathogenic characteristics of Pseudomonas aeruginosa bacteraemia isolates in a high-endemicity setting for ST175 and ST235 high-risk clones. Eur J Clin Microbiol Infect Dis Off Publ Eur Soc Clin Microbiol. 2020; 39(4):671-8.
17. Sánchez-Diener I, et al. Weighting the impact of virulence on the outcome of Pseudomonas aeruginosa bloodstream infections. Clin Microbiol Infect. 2020; 26(3):351-7.
18. Mermel LA, et al. Clinical practice guidelines for the diagnosis and management of intravascular catheter-related infection: 2009 Update by the Infectious Diseases Society of America. Clin Infect Dis. 2009; 49(1):1-45.
19. Capdevila JA, et al. 2016 Expert consensus document on prevention, diagnosis and treatment of short-term peripheral venous catheter-related infections in adult. Rev Esp Quimioter. 2016; 29(4):230-8.
20. Bae M, Jeong Y, Bae S, Kim MJ, Chong YP, Kim S-H, et al. Short versus prolonged courses of antimicrobial therapy for patients with uncomplicated Pseudomonas aeruginosa bloodstream infection: a retrospective study. J Antimicrob Chemother. 2021; 77(1):223-228.
21. McCarthy KL, Paterson DL. Increased risk of death with recurrent Pseudomonas aeruginosa bacteremia. Diagn Microbiol Infect Dis. 2017; 88(2):152-7.
22. Molina J, Cisneros JM. Editorial Commentary: A Chance to Change the Paradigm of Outcome Assessment of Antimicrobial Stewardship Programs. Clin Infect Dis. 2015; 61(5):807-8.
23. Evans SR, et al. Desirability of Outcome Ranking (DOOR) and Response Adjusted for Duration of Antibiotic Risk (RADAR). Clin Infect Dis. 2015; 61(5):800-6.
24. Evans SR, et al. Methodologies for pragmatic and efficient assessment of benefits and harms: Application to the SOCRATES trial. Clin Trials 2020; 17(6):617-26.
25. Cabellos C, et al. Invasive meningococcal disease: Impact of short course therapy. A DOOR/RADAR study. J Infect. 2017; 75(5):420-5.
26. Celestin AR, et al. Novel Method Suggests Global Superiority of Short-Duration Antibiotics for Intra-abdominal Infections. Clin Infect Dis. 2017; 65(9):1577-9.
27. Schweitzer VA, et al. Response Adjusted for Days of Antibiotic Risk (RADAR): evaluation of a novel method to compare strategies to optimize antibiotic use. Clin Microbiol Infect. 2017; 23(12):980-5.
28. Ianiro G, et al. Antibiotics as deep modulators of gut microbiota: between good and evil. Gut. 2016; 65(11):1906-15.

29. Mu C, Zhu W. Antibiotic effects on gut microbiota, metabolism, and beyond. Appl Microbiol Biotechnol. 2019; 103(23-24):9277-85.
30. Elvers KT, et al. Antibiotic-induced changes in the human gut microbiota for the most commonly prescribed antibiotics in primary care in the UK: a systematic review. BMJ Open. 2020; 10(9):e035677.
31. Gori S, et al. Gut microbiota and cancer: How gut microbiota modulates activity, efficacy and toxicity of antitumoral therapy. Crit Rev Oncol Hematol. 2019; 143:139-47.
32. Taur Y, et al. The effects of intestinal tract bacterial diversity on mortality following allogeneic hematopoietic stem cell transplantation. Blood. 2014; 124(7):1174-82.
33. Korn EL, Freidlin B. Interim monitoring for non-inferiority trials: minimizing patient exposure to inferior therapies. Ann Oncol. 2018; 29(3):573-7.
34. EUCAST: Clinical breakpoints and dosing of antibiotics [citado 17 de marzo de 2021]. https://www.eucast.org/clinical_breakpoints/
35. Magiorakos AP, Srinivasan A, Carey RB, et al. Multidrug-resistant, extensively drug-resistant and pandrug-resistant bacteria: an international expert proposal for interim standard definitions for acquired resistance. Clin Microbiol Infect. 2012;18(3):268-281.

ANEXO II: LISTADO DE CENTROS PARTICIPANTES

CENTRO	INVESTIGADOR PRINCIPAL
Hospital Universitario Virgen del Rocío	Dra. María del Rocío Álvarez Marín
Hospital Universitario Virgen Macarena	Dr. Luis Eduardo López Cortés
Hospital Universitario Virgen de Valme	Dr. Nicolás Merchante Gutiérrez
Hospital Universitario Reina Sofía	Dr. Juan José Castón Osorio
Hospital Universitario Virgen de las Nieves	Dr. Miguel Ángel López Zúñiga
Hospital Universitario Clínico San Cecilio	Dr. Francisco Anguita Santos
Hospital Universitario Torrecárdenas	Dra. María Ángeles Esteban Moreno
Hospital Universitario Regional de Málaga	Dr. Juan Diego Ruiz Mesa
Hospital Costa del Sol	Dr. Alfonso del Arco Jiménez
Hospital Universitario Virgen de la Victoria	Dra. Rosario Palacios Muñoz
Hospital Universitario Juan Ramón Jiménez	Dra. María Franco Huerta
Hospital Universitario de Jerez de la Frontera	Dr. Antonio Jesús Hidalgo Castellón
Hospital Universitario de Puerto Real	Dra. Patricia Jiménez Aguilar
Complejo Hospitalario de Jaén	Dra. Carmen Herrero Rodríguez
Hospital Universitario Ramón y Cajal	Dra. Pilar Martín Dávila
Hospital Universitario La Paz	Dra. María Belén Loeches Yagüe
Hospital Universitario Cruces	Dra. Ane Josune Goikoetxea Agirre
Hospital Universitario de Donostia	Dra. Maialen Ibarguren Pinilla
Hospital Universitario San Pedro	Dr. José Antonio Oteo Revuelta
Hospital Universitario Marqués de Valdecilla	Dra. M. Carmen Fariñas Álvarez
Hospital Universitario Central de Asturias	Dr. Emilio García Prieto
Complexo Hospitalario Universitario de Vigo	Dr. Adrián Sousa Domínguez

Complexo Hospitalario Universitario A Coruña	Dra. Mª Dolores Sousa Regueiro
Hospital Universitario Lucus Augusti	Dra. Blanca Ayuso García
Hospital Clínico Universitario Lozano Blesa	Dr. José Ramón Paño Pardo
Hospital Universitario de Bellvitge	Dra. Mireia Puig Asensio
Hospital Parc Taulí	Dra. Aina Gomila Grange
Hospital Universitari Vall d'Hebron	Dr. Ibai Los Arcos Bertiz
Hospital Universitario Son Espases	Dra. Helem Vílchez Rueda
Hospital Universitario La Fe	Dra. Paula Ramírez Galleymore

ANEXO III. DIAGRAMA DE FLUJO

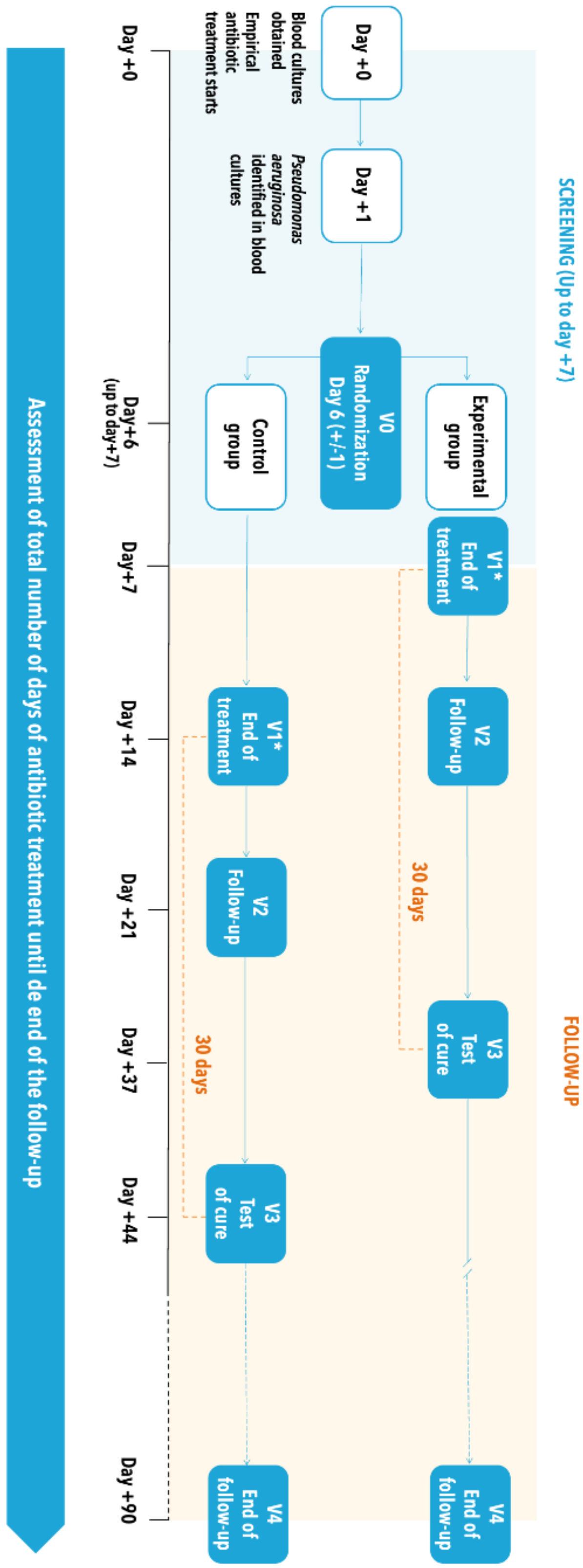

* Antibiotic treatment will be stopped at this point if the patient remain apyretic and without symptoms of infection for at least 72 hours. Antibiotic treatment could be resumed after this point whenever considered necessary by the clinician in charge of the patient if an unfavourable course was observed.

ANEXO IV. EUCAST

https://www.eucast.org/fileadmin/src/media/PDFs/EUCAST_files/Breakpoint_tables/v_11.0_Breakpoint_Tables.pdf

ANEXO V. MATERIAL DIDÁCTICO

GUÍA RÁPIDA PARA MEJORAR EL TRATAMIENTO DE LAS INFECCIONES POR *PSEUDOMONAS AERUGINOSA*

1 QUÉ ANTIBIÓTICO

- La "I" del antibiograma de *P. aeruginosa* ya no significa "sensibilidad intermedia"; **significa "incrementa la exposición al antibiótico"**. Este cambio de nomenclatura afecta solo a algunos antibióticos.
- Con una dosis y administración correctas, **puedes usar sin problema cualquier opción que no se clasifique como "R"** (sea "S" o "I").

	Umbral de CMI para considerar el tratamiento activo (mg/L)		Umbral de CMI para considerar el tratamiento activo (mg/L)
Ciprofloxacino	≤0,5	Imipenem	≤4
Levofloxacino	≤1	Meropenem	≤8
Piperacillina/tazobactam	≤16	Meropenem-vaborbactam	≤8
Ceftazidima	≤8	Colistina	≤2
Cefepima	≤8	Amikacina	≤16
Ceftazidima/avibactam	≤8	Tobramicina	≤2
Ceftolozano/tazobactam	≤4	Aztreonam	≤16

- **Evita los agentes con una CMI exactamente en el umbral** (p.e. ceftazidima = 8 mg/L, piperacilina/taz = 16 mg/L, etc.) en infecciones graves o focos complicados. Es posible un mayor riesgo de fracaso.

2 QUÉ DOSIS

- Las infecciones por *P. aeruginosa* requieren **dosis más altas**:

	Dosis antipseudomónicas	
**Fluorquinolonas**	**Vía oral**	**Vía intravenosa**
**Ciprofloxacino**	750 mg cada 12h	400 mg cada 8h
**Levofloxacino**	500 mg cada 12h	500 mg cada 12h
**Piperacillina/tazobactam**	4 g / 0.5 g cada 8h administrado en perfusión extendida de 4h.<br>4 g / 0.5 g cada 6h en perfusión extendida de 4h en infecciones graves por cepas resistentes a ceftazidima o neumonías.	
**Ceftazidima, cefepima**	2 g cada 8h	
**Ceftazidima/avibactam**	2 g cada 8h administrado en perfusión de 2 h	
**Ceftolozano/tazobactam**	1 g / 0.5 g cada 8h<br>Para neumonía, la dosis apropiada es 2 g / 1 g cada 8h	
**Imipenem**	1 g cada 6h	
**Meropenem**	**CMI ≤2 mg/L:** 1 g cada 8h<br>**CMI > 2 mg/l y ≤ 8 mg/L:** 2g cada 8h en perfusión de 3h	
**Meropenem-vaborbactam**	2 g / 2 g cada 8h en perfusión de 3 h	
**Colistina**	4,5 MU cada 12h	
**Amikacina**	25-30 mg/kg cada 24h	
**Tobramicina**	6-7 mg/kg cada 24h	
**Aztreonam**	2 g cada 6h	

3 QUÉ MODO DE ADMINISTRACIÓN

- Si vas a usar betalactámicos, **adminístralos siempre en perfusión extendida o continua.** Esto **ha demostrado reducir la mortalidad** de las infecciones graves por *P. aeruginosa*.

4 MONOTERAPIA O COMBINACIÓN

- Si dispones de una opción de **tratamiento plenamente activa, úsala en monoterapia.** Los tratamientos combinados no han demostrado más efectividad, pero sí más riesgo de efectos secundarios.

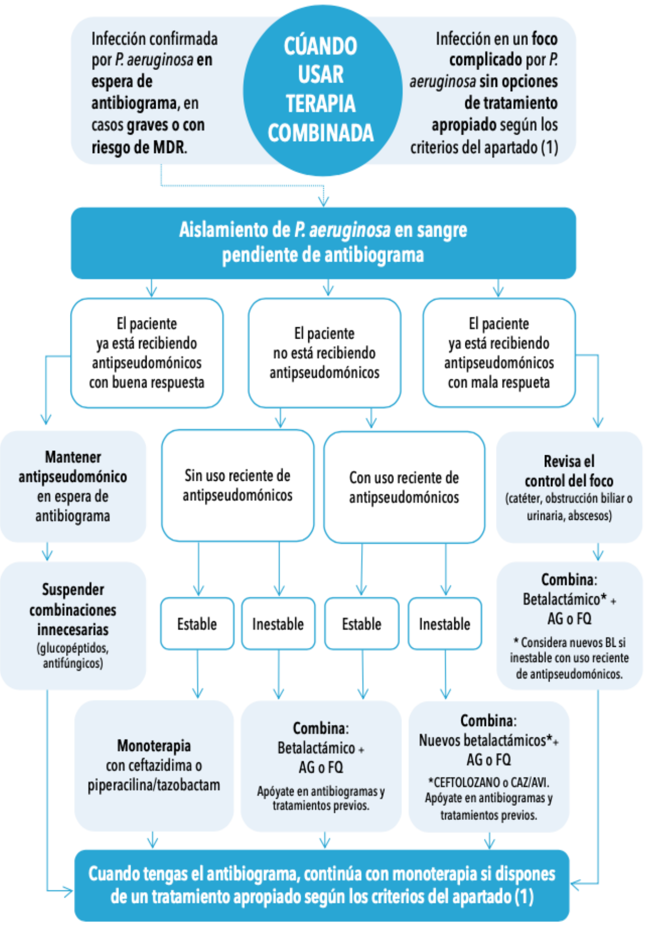

En infecciones en focos complicados por cepas sin una opción de tratamiento plenamente activa, puede ser necesario prolongar el tratamiento combinado.

AG = Aminoglucósido (tobramicina o amikacina) **FQ** = Fluorquinolonas **CAZ/AVI**: Ceftazidima/avibactam

5 CONTROLA EL FOCO

- Retira ese catéter, drena ese absceso… *P. aeruginosa* tiene una gran capacidad de desarrollar resistencias durante el tratamiento.

Referencias
- EUCAST Breakpoint tables for interpretation of MICs and zone diameters, version 10.0, 2021.
- Prolonged versus short-term intravenous infusion of antipseudomonal β-lactams for patients with sepsis: a systematic review and meta-analysis of randomised trials. Vardakas et al. Lancet ID 2018.
- Impact of borderline minimum inhibitory concentration on the outcome of invasive infections caused by Enterobacteriaceae treated with β-lactams: a systematic review and meta-analysis. Torres et al. Eur J Clin Microbiol Infect Dis, 2015.

ANEXO VI. CUESTIONARIO PARA VISITAS 3, 4 Y NO PLANIFICADAS

La visita 3 (día +30 desde la interrupción del tratamiento antibiótico) y la visita 4 (día +90 desde la fecha de extracción del primer hemocultivo positivo) se pueden realizar por vía telefónica si el paciente no se encuentra hospitalizado. Con el fin de facilitar la recogida de datos fundamentales para completar el cuaderno de recogida de datos se propone el siguiente cuestionario estructurado:

1. ¿Cuándo recibió el alta hospitalaria?

2. Si no había completado el tratamiento antibiótico antes del alta hospitalaria, ¿ha continuado recibiendo tratamiento antibiótico en su domicilio por vía intravenosa u oral? En caso positivo:

    a) ¿Qué tratamiento ha recibido?

    b) ¿Cuándo lo inició y cuándo ha finalizado?

3. Desde que interrumpió el tratamiento antibiótico del ensayo:

    a) ¿Han vuelto a aparecer los síntomas de infección? (Aludir a los síntomas locales que se presentaron al inicio del episodio infeccioso por el que fue incluido en el estudio)

    b) ¿Ha presentado fiebre en algún momento? Si la respuesta es positiva, ¿la fiebre se ha acompañado de algún otro signo o síntoma de infección? Especificar.

    c) ¿Le han tomado muestras ante la sospecha de una posible infección (hemocultivo, cultivo de orina, etc.)? Si la respuesta es positiva:

        - ¿Cuándo se tomó la muestra?
        - ¿Qué tipo de muestra?
        - ¿Conoce el resultado de ese cultivo?

    d) ¿Ha requerido ingreso hospitalario? Si la respuesta es positiva:

        - ¿Cuál ha sido el motivo?
        - ¿Podría indicar el centro donde ingresó y las fechas de ingreso y alta?

    e) ¿Ha requerido recibir tratamiento antibiótico por cualquier motivo? En caso positivo:

        - ¿Cuál ha sido el motivo?

- ¿Qué tratamiento ha recibido (antibiótico, dosis, frecuencia y vía de administración)?
- ¿Cuándo comenzó y finalizó el tratamiento?
- ¿Por qué ha interrumpido el tratamiento (paso a vía oral, final de tratamiento, evolución desfavorable, sobreinfección, efecto adverso u otros)? Especificar.

f) ¿Ha tenido algún efecto secundario que se haya relacionado con el tratamiento antibiótico que recibió?

- ¿Ha tenido diarrea (más de 3 deposiciones al día en pacientes con hábito intestinal previo normal)?
- ¿Ha detectado alguna erupción cutánea?
- ¿Su médico ha detectado alguna alteración analítica que haya puesto en relación con el tratamiento antibiótico previo?

ANEXO VII. ALGORITMO DE ADSCRIPCIÓN DE LOS PACIENTES A LAS CATEGORÍAS DE RECURRENCIA PROBADA, PROBABLE O POSIBLE.

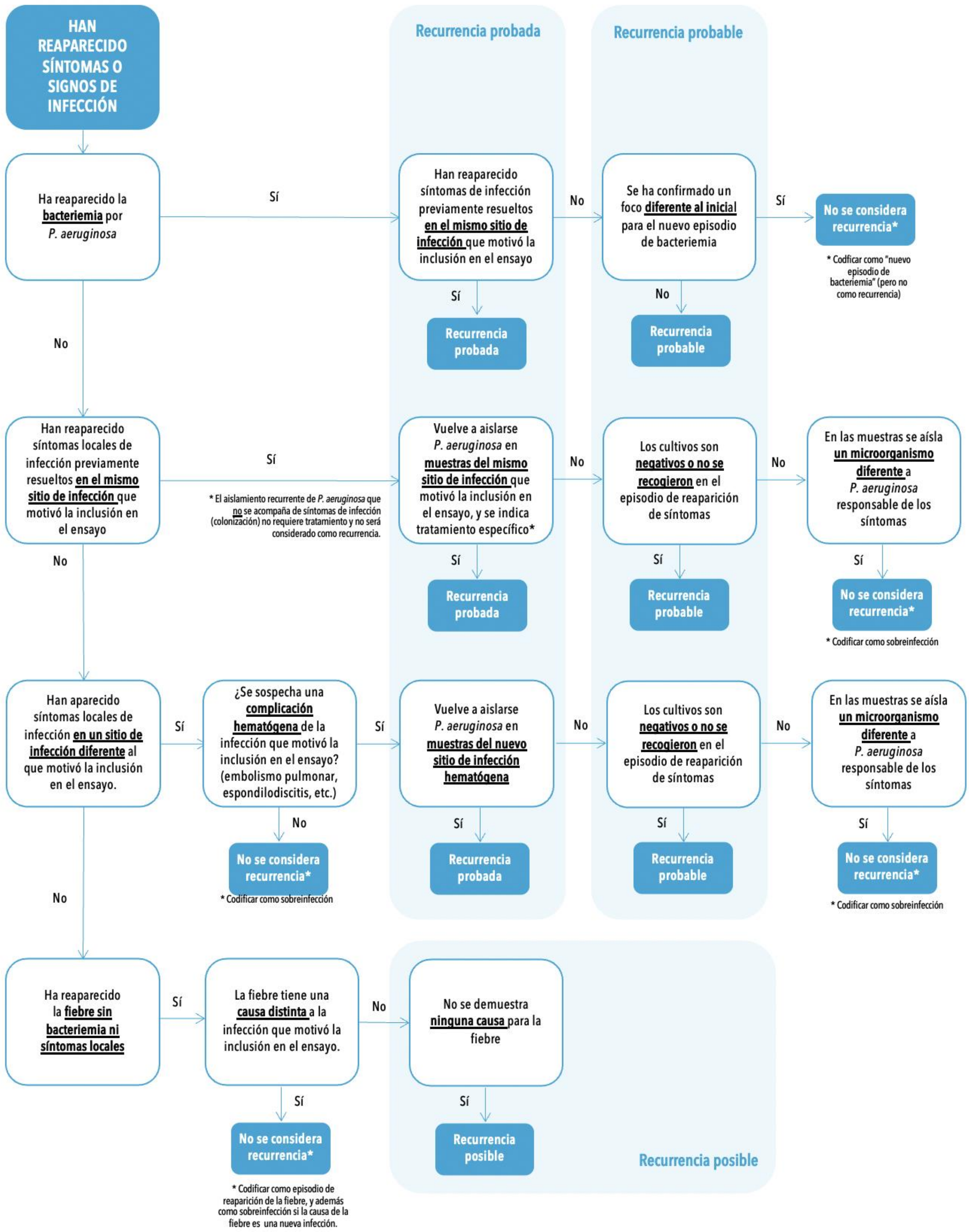

ANEXO VIII. HOJA DE INFORMACIÓN AL PACIENTE Y CONSENTIMIENTO INFORMADO.

Se adjunta en un documento aparte.

ANEXO IX. ÍNDICE DE CHARLSON

PUNTUACIÓN	CONDICIÓN
1	Infarto de miocardio<br>Insuficiencia cardiaca congestiva<br>Enfermedad vascular periférica<br>Enfermedad cerebrovascular<br>Demencia<br>Enfermedad pulmonar crónica<br>Enfermedad del tejido conectivo<br>Úlcera péptica<br>Enfermedad hepática leve<br>Diabetes sin afectación órgano diana
2	Hemiplejía o paraplejía<br>Enfermedad renal moderada o grave<br>Diabetes con afectación en órgano diana<br>Cualquier tumor
3	Enfermedad hepática moderada o grave
6	Tumor sólido metastásico<br>SIDA

Charlson ME, Pompei P, Ales KL, MacKenzie CR. A new method of classifying prognostic comorbidity in longitudinal studies: development and validation. J ChronDis 1987; 40: 373-383.

ANEXO X. PITT SCORE

Recoger la puntuación más alta entre el día de la bacteriemia y las 48 horas anteriores.

CRITERIO		PUNTOS
Fiebre (Tª)	≤ 35ºC ó ≥40ºC	2
	35,1-36 ó 39-39,9	1
	36,1-38,9	0
Hipotensión	Disminución aguda de la TA sistólica de 30 mmHg o diastólica de 20 mmHg	2
	requerimiento de drogas vasopresoras intravenosas	2
	TA sistólica < 90 mmHg	2
Ventilación mecánica		2
Fallo cardiaco		4
Estado mental	Alerta	0
	Desorientado	1
	Estuporoso	2
	Coma	4

ANEXO XI. PROCEDIMIENTO DE ENVÍO DE AISLAMIENTOS MICROBIOLÓGICOS Y DE MUESTRAS DE HECES FECALES PARA ESTUDIOS DE MICROBIOTA INTESTINAL.

Procedimientos para el envío de los aislamientos microbiológicos:

1. Los microorganismos una vez identificados deberán congelarse a -80ºC siguiendo el procedimiento habitual de cada centro. Se almacenará y permanecerá sin interrupciones hasta el momento de su envío. Cualquier pérdida (aunque sea transitoria) de las condiciones de conservación de las muestras se comunicará al Centro de coordinación del estudio.

2. Inmediatamente después de su obtención cada muestra se identificará y numerará de manera inequívoca con una etiqueta en la que constará el Código de identificación del Centro, el número de identificación del paciente en el estudio, el número de identificación de la muestra y la fecha de obtención de la muestra.

3. Hasta su envío las muestras permanecerán en el Centro participante bajo la responsabilidad de los investigadores del mismo. El traslado de todas las muestras de cada Centro se realizará en envíos sucesivos a través de una empresa de transporte (Enviplus Sl, Sevilla, España). Desde el centro de coordinación del estudio, se especificará y acordará con cada centro el día y detalles concretos de recogida y envío de las muestras.

4. Cada 3 meses deberá prepararse un envío al centro de referencia. El traslado de todas las muestras de cada Centro se realizará en envíos sucesivos a través de una empresa de transporte (Enviplus Sl, Sevilla, España). Desde el centro de coordinación del estudio, se especificará y acordará con cada centro el día y detalles concretos de recogida y envío de las muestras.

5. A partir de las cepas congeladas se realizará un subcultivo en una placa de agar sangre. Se procederá a incubar en estufa a 37ºC entre 18-20h hasta conseguir un crecimiento bacteriano adecuado. El crecimiento en estas placas servirá de inóculo para el transporte.

6. Los microorganismos se enviarán con torunda en medio de transporte Amies, Stuart o Cary-Blair a temperatura ambiente. Para ello se recogerá con una torunda estéril una cantidad abundante de microorganismos de la superficie del agar de la placa subcultivada. Se introducirá la torunda en el tubo que incorpora el medio de transporte. Encajar el tapón en el tubo asegurando un buen cierre. **Identificar el aislamiento de manera clara en la etiqueta de cada tubo utilizando el número de referencia de la muestra del centro remitente.**

7. Cada aislado debe estar identificado correctamente e ir acompañado de su correspondiente formulario de aislamiento (Anexo XII) y la copia del informe del antibiograma del centro remitente.

8. Las muestras se enviarán a:

A/A Mª Eugenia Pachón Ibáñez
Instituto de Biomedicina de Sevilla (IBiS)
Laboratorio 208, segunda planta
C/ Antonio Maura Montaner, S/N
41013, Sevilla.
Teléfono: 955923100 7 650720131
mpachon-ibis@us.es

9. Las muestras deben ser embaladas y transportadas como sustancias infecciosas de categoría A, de acuerdo, a la normativa vigente por la OMS (Guía sobre la reglamentación del transporte de sustancias infecciosas 2011-2012) y BOE nº 63 (14/3/2013).

10. Una vez recogidas por la mensajería, contactar con el centro de referencia vía correo electrónico (mpachon-ibis@us.es) indicando en asunto "ENVIO SHORTEN II_Nombre del hospital remitente" para confirmar el envío de muestras indicando en el texto del correo electrónico:

a. Nombre del hospital

b. Fecha de salida del envío

c. Nombre y teléfono de persona de contacto que realiza el envío

d. Número de microorganismos enviados

e. Confirmación de que el formulario correspondiente a cada cepa ha sido incluido en el envío.

11. Una vez registradas las muestras, se mandarán todos los aislados *P. aeruginosa* que cumplan los criterios de bacteriemia recurrente o vayan a ser utilizados como controles para estudios de identidad clonal. Se acordará con Mercedes Delgado del Servicio de Microbiología del Hospital Universitario Virgen Macarena el día y detalles concretos del envío de las muestras.

Muestras fecales para análisis de la microbiota

1. Los tubos de colección de muestras fecales para los estudios de microbiota (*Stool Nucleic Acid Collection and Preservation Tubes* [Cat. 45660, Norgen Biotek Corp.]) se enviarán desde el centro de referencia (IBiS) a los distintos centros participantes.

2. Una vez se acuerde entre los centros participantes y el IBiS, se recogerán las muestras. Estos tubos permiten la conservación y el envío de las muestras a temperatura ambiente; según especificaciones también se pueden almacenar a -20ºC o -70ºC.

Procedimiento para la recogida de muestra:

- Colocar la "cuña" junto con una empapadera sobre el váter.
- Retirar una pequeña muestra de 3 sitios distintos usando la cucharilla del botecito.
- No superar el nivel que marca el bote.
- Apretar el tapón del bote de tal modo que al agitar suavemente quede todo mezclado.

3. Inmediatamente después de su obtención cada muestra:

- Se identificará de manera inequívoca con una etiqueta en la que constará el Código de identificación del Centro, el número de identificación del paciente en el estudio, el número de identificación de la muestra y la fecha de obtención de la muestra.
- Correctamente identificada, se almacenará y permanecerá sin interrupciones hasta el momento de su envío. Cualquier pérdida (aunque sea transitoria) de las condiciones de conservación de las muestras se comunicará al Centro de coordinación del estudio. Hasta su envío las muestras permanecerán en el Centro participante bajo la responsabilidad de los investigadores del mismo.

4. El traslado de todas las muestras de cada Centro se realizará en envíos sucesivos a través de una empresa de transporte (Enviplus Sl, Sevilla, España). Se especificará y acordará con cada centro el día y detalles concretos de recogida y envío de las muestras.

Las muestras se enviarán para la secuenciación de las regiones hipervariables de ARNr 16S V3-V4, utilizando la plataforma Illumina MiSeq, y realizar posteriormente los análisis bioinformáticos para estudiar la diversidad bacteriana en la microbiota intestinal de las muestras de ambos grupos de estudio a la siguiente dirección: :

A/A Mª Eugenia Pachón Ibáñez
Instituto de Biomedicina de Sevilla (IBiS)
Laboratorio 208, segunda planta
C/ Antonio Maura Montaner, S/N
41013, Sevilla.
Teléfono: 955923100 7 650720131
mpachon-ibis@us.es

ANEXO XII: FORMULARIO DE AISLADO

NÚMERO DE REFERENCIA<br>*(a rellenar por centro de referencia)*	
NOMBRE DE CENTRO REMITENTE	
FECHA DE AISLAMIENTO	
NÚMERO DE REFERENCIA DE LA MUESTRA EN CENTRO REMITENTE	
SERVICIO O UNIDAD DE ORIGEN	
METODO DE IDENTIFICACION EN CENTRO REMITENTE	
MÉTODO DE ANTIBIOGRAMA EN CENTRO REMITENTE	
DATOS DE SENSIBILIDAD ANTIMICROBIANA<br>(se recomienda que adjunten copia del informe emitido en el centro de origen)	

ANEXO XIII. FORMULARIO DE NOTIFICACIÓN DE AAG

Código Sujeto: ________________

TIPO DE INFORME: ☐ Inicial ☐ Seguimiento

1. INFORMACIÓN DEL CENTRO

Nº de centro:	Investigador Principal:
Persona que notifica el AAG:	Tfno:
Fax:	Mail:

2. INFORMACIÓN DEL SUJETO

Sexo	Edad (Al comienzo del AA)	Grupo de edad (rellenar sólo si se desconoce la edad del sujeto)	Fecha de nacimiento (dd-mmm-aaaa)	Peso (kg)	Altura (cm)
☐ Masculino<br>☐ Femenino	_________<br>☐ Años ☐ Meses ☐ Días	☐ Neonato ☐ Lactante<br>☐ Niño ☐ Adolescente<br>☐ Adulto ☐ Anciano	_ _ – _ _ _ – _ _ _ _		

3. ACONTECIMIENTO ADVERSO.

Acontecimiento adverso grave (Especificar el diagnóstico o síndrome, si se conoce. Si se desconoce incluir signos y síntomas.)**:**

………

………

DESCRIPCIÓN DEL ACONTECIMIENTO ADVERSO (Proporcionar toda la información acerca de las circunstancias, la secuencia, el diagnóstico y el tratamiento del AA)

Fecha de inicio (dd-mmm-aaaa):_ _ – _ _ _ – _ _ _ _

Fecha de fin: (dd-mmm-aaaa):_ _ – _ _ _ – _ _ _ _

Criterio de gravedad:	Desenlace (estado en el momento de la notificación):
☐ Muerte	☐ Recuperado
☐ Amenaza para la vida	☐ En recuperación
☐ Requiere o prolonga hospitalización	☐ No recuperado
☐ Incapacidad permanente o significativa	☐ Recuperado con secuelas
☐ Defecto de nacimiento o anomalía congénita	☐ Mortal
☐ Clínicamente relevante	☐ Desconocido

En caso de fallecimiento cumplimentar los siguientes datos:

Fecha de exitus: _ _ – _ _ _ – _ _ _ _ Causa:________________________________

Se practicó autopsia: ☐ Si ☐ No ☐ Desconocido

Indicar la causa de fallecimiento según la autopsia: ________________________________

4. PRODUCTO/MEDICAMENTO EN INVESTIGACIÓN (Indicar el/los tratamiento/s asignado/s al sujeto tras la aleatorización)
Si necesita mas espacio, utilizar copias de esta página y marcar esta casilla ☐

Medicamento	Dosis y unidades	Frecuencia	Vía	Fecha de inicio (dd-mmm-aaaa)	Fecha de fin (dd-mmm-aaaa) (si continúa marcar la casilla)	Relación causal
					☐	☐Relacionado<br>☐No relacionado
					☐	☐Relacionado<br>☐No relacionado
					☐	☐Relacionado<br>☐No relacionado

Acción tomada con la medicación en respuesta al AA	☐Retirada de la medicación ☐Disminución de la dosis ☐Sin cambios<br>☐Interrupción temporal de la medicación ☐Aumento de la dosis ☐No aplica<br>☐Desconocido
¿Remitió el AA al suspender la medicación o reducir la dosis?	☐Si ☐No ☐Desconocido ☐No aplica
¿Reapareció el AA al reintroducir la medicación?	☐Si ☐No ☐Desconocido ☐No aplica

5. MEDICACION CONCOMITANTE (Incluir aquellos tratamientos concomitantes y de base que ha tomado en las dos semanas previas a la fecha de inicio del AA. No incluir el tratamiento administrado para tratar el AA o aquel administrado después de la fecha de inicio del AA)
Si necesita mas espacio, utilizar copias de esta página y marcar esta casilla☐

Medicamento	Dosis diaria (unidades)	Frecuencia	Vía	Fecha de inicio (dd-mmm-aaaa)	Fecha de fin (dd-mmm-aaaa) (si continúa marcar la casilla)	Relación causal	Indicación
					☐	☐Relacionado<br>☐No relacionado	
					☐	☐Relacionado<br>☐No relacionado	
					☐	☐Relacionado<br>☐No relacionado	

Acción tomada con la medicación en respuesta al AA	☐Retirada de la medicación ☐Disminución de la dosis ☐Sin cambios<br>☐Interrupción temporal de la medicación ☐Aumento de la dosis ☐No aplica<br>☐Desconocido
¿Remitió el AA al suspender la medicación o reducir la dosis?	☐Si ☐No ☐Desconocido ☐No aplica
¿Reapareció el AA al reintroducir la medicación?	☐Si ☐No ☐Desconocido ☐No aplica

6. CAUSA ALTERNATIVA

¿Existe alguna posibilidad de que el AA esté relacionado con alguna otra causa alternativa a la medicación?
☐Si ☐No En caso afirmativo, especificar (ampliar información en el apartado de descripción del AAG si fuera necesario):
____
____

7. HISTORIA MÉDICA RELEVANTE
Si necesita más espacio, utilizar copias de esta página y marcar esta casilla☐

Antecedente patológico	Fecha de inicio (dd-mmm-aaaa)	Fecha de fin (dd-mmm-aaaa)	(si continúa marcar la casilla)
			☐
			☐
			☐
			☐

Información complementaria de los datos de la Historia Médica

8. DATOS DE LABORATORIO Y OTRAS EXPLORACIONES COMPLEMENTARIAS: (indicar únicamente los resultados de pruebas relevantes para documentar el AAG notificado)
Si necesita mas espacio, utilizar copias de esta página y marcar esta casilla☐

TEST REALIZADO	FECHA TEST (dd-mmm-aaaa)	RESULTADO (Unidades)	Rango Referencia	COMENTARIO

Información complementaria de los datos de laboratorio y otras exploraciones:

Firma del investigador que notifica	Fecha de notificación
**Firma del Responsable de Farmacovigilancia**	**Fecha de recepción de la notificación**

ENVIAR INMEDIATAMENTE POR FAX AL NODO DE FARMACOVIGILANCIA DE LA UICEC DEL HOSPITAL UNIVERSITARIO VIRGEN DEL ROCÍO (FAX: 955095338)

email: pv_shorten2@scren.es

ANEXO XIV. DECLARACIÓN DE HELSINKI DE LA ASOCIACIÓN MÉDICA MUNDIAL

Principios éticos para las investigaciones médicas en seres humanos
Adoptada por la 18ª Asamblea Médica Mundial, Helsinki, Finlandia, junio 1964 y enmendada por la
29ª Asamblea Médica Mundial, Tokio, Japón, octubre 1975
35ª Asamblea Médica Mundial, Venecia, Italia, octubre 1983
41ª Asamblea Médica Mundial, Hong Kong, septiembre 1989
48ª Asamblea General Somerset West, Sudáfrica, octubre 1996
52ª Asamblea General, Edimburgo, Escocia, octubre 2000
Nota de Clarificación del Párrafo 29, agregada por la Asamblea General de la AMM, Washington 2002
Nota de Clarificación del Párrafo 30, agregada por la Asamblea General de la AMM, Tokio 2004
59ª Asamblea General, Seúl, Corea, octubre 2008
64ª Asamblea General, Fortaleza, Brasil, octubre 2013

A. INTRODUCCIÓN

1. La Asociación Médica Mundial (AMM) ha promulgado la Declaración de Helsinki como una propuesta de principios éticos para investigación médica en seres humanos, incluida la investigación del material humano y de información identificables.
La Declaración debe ser considerada como un todo y un párrafo debe ser aplicado con consideración de todos los otros párrafos pertinentes.
2. Conforme al mandato de la AMM, la Declaración está destinada principalmente a los médicos. La AMM insta a otros involucrados en la investigación médica en seres humanos a adoptar estos principios.

B. PRINCIPIOS GENERALES

3. La Declaración de Ginebra de la Asociación Médica Mundial vincula al médico con la fórmula "velar solícitamente y ante todo por la salud de mi paciente", y el Código Internacional de Ética Médica afirma que: "El médico debe considerar lo mejor para el paciente cuando preste atención médica".
4. El deber del médico es promover y velar por la salud, bienestar y derechos de los pacientes, incluidos los que participan en investigación médica. Los conocimientos y la conciencia del médico han de subordinarse al cumplimiento de ese deber.
5. El progreso de la medicina se basa en la investigación que, en último término, debe incluir estudios en seres humanos.
6. El propósito principal de la investigación médica en seres humanos es comprender las causas, evolución y efectos de las enfermedades y mejorar las intervenciones preventivas,

diagnósticas y terapéuticas (métodos, procedimientos y tratamientos). Incluso, las mejores intervenciones probadas deben ser evaluadas continuamente a través de la investigación para que sean seguras, eficaces, efectivas, accesibles y de calidad.
7. La investigación médica está sujeta a normas éticas que sirven para promover y asegurar el respeto a todos los seres humanos y para proteger su salud y sus derechos individuales.
8. Aunque el objetivo principal de la investigación médica es generar nuevos conocimientos, este objetivo nunca debe tener primacía sobre los derechos y los intereses de la persona que participa en la investigación.
9. En la investigación médica, es deber del médico proteger la vida, la salud, la dignidad, la integridad, el derecho a la autodeterminación, la intimidad y la confidencialidad de la información personal de las personas que participan en la investigación. La responsabilidad de la protección de las personas que toman parte en la investigación debe recaer siempre en un médico u otro profesional de la salud y nunca en los participantes en la investigación, aunque hayan otorgado su consentimiento.
10. Los médicos deben considerar las normas y estándares éticos, legales y jurídicos para la investigación en seres humanos en sus propios países, al igual que las normas y estándares internacionales vigentes. No se debe permitir que un requisito ético, legal o jurídico nacional o internacional disminuya o elimine cualquiera medida de protección para las personas que participan en la investigación establecida en esta Declaración.
11. La investigación médica debe realizarse de manera que reduzca al mínimo el posible daño al medio ambiente.
12. La investigación médica en seres humanos debe ser llevada a cabo sólo por personas con la educación, formación y calificaciones científicas y éticas apropiadas. La investigación en pacientes o voluntarios sanos necesita la supervisión de un médico u otro profesional de la salud competente y calificado apropiadamente.
13. Los grupos que están subrepresentados en la investigación médica deben tener un acceso apropiado a la participación en la investigación.
14. El médico que combina la investigación médica con la atención médica debe involucrar a sus pacientes en la investigación sólo en la medida en que esto acredite un justificado valor potencial preventivo, diagnóstico o terapéutico y si el médico tiene buenas razones para creer que la participación en el estudio no afectará de manera adversa la salud de los pacientes que toman parte en la investigación.
15. Se debe asegurar compensación y tratamiento apropiados para las personas que son dañadas durante su participación en la investigación.

C. RIESGOS, COSTOS Y BENEFICIOS

16. En la práctica de la medicina y de la investigación médica, la mayoría de las intervenciones implican algunos riesgos y costos.

La investigación médica en seres humanos sólo debe realizarse cuando la importancia de su objetivo es mayor que el riesgo y los costos para la persona que participa en la investigación.
17. Toda investigación médica en seres humanos debe ser precedida de una cuidadosa comparación de los riesgos y los costos para las personas y los grupos que participan en la investigación, en comparación con los beneficios previsibles para ellos y para otras personas o grupos afectados por la enfermedad que se investiga.
Se deben implementar medidas para reducir al mínimo los riesgos. Los riesgos deben ser monitoreados, evaluados y documentados continuamente por el investigador.
18. Los médicos no deben involucrarse en estudios de investigación en seres humanos a menos de que estén seguros de que los riesgos han sido adecuadamente evaluados y de que es posible hacerles frente de manera satisfactoria.
Cuando los riesgos que implican son más importantes que los beneficios esperados o si existen pruebas concluyentes de resultados definitivos, los médicos deben evaluar si continúan, modifican o suspenden inmediatamente el estudio.

D. GRUPOS Y PERSONAS VULNERABLES
19. Algunos grupos y personas sometidas a la investigación son particularmente vulnerables y pueden tener más posibilidades de sufrir abusos o daño adicional.
Todos los grupos y personas deben recibir protección específica.
20. La investigación médica en un grupo vulnerable sólo se justifica si la investigación responde a las necesidades o prioridades de salud de este grupo y la investigación no puede realizarse en un grupo no vulnerable. Además, este grupo podrá beneficiarse de los conocimientos, prácticas o intervenciones derivadas de la investigación.

E. REQUISITOS CIENTÍFICOS Y PROTOCOLOS DE INVESTIGACIÓN
21. La investigación médica en seres humanos debe conformarse con los principios científicos generalmente aceptados y debe apoyarse en un profundo conocimiento de la bibliografía científica, en otras fuentes de información pertinentes, así como en experimentos de laboratorio correctamente realizados y en animales, cuando sea oportuno. Se debe cuidar también del bienestar de los animales utilizados en los experimentos.
22. El proyecto y el método de todo estudio en seres humanos deben describirse claramente y ser justificados en un protocolo de investigación.
El protocolo debe hacer referencia siempre a las consideraciones éticas que fueran del caso y debe indicar cómo se han considerado los principios enunciados en esta Declaración. El protocolo debe incluir información sobre financiamiento, patrocinadores, afiliaciones institucionales, posibles conflictos de interés e incentivos para las personas del estudio y la información sobre las estipulaciones para tratar o compensar a las personas que han sufrido daños como consecuencia de su participación en la investigación.

En los ensayos clínicos, el protocolo también debe describir los arreglos apropiados para las estipulaciones después del ensayo.

F. COMITÉS DE ÉTICA DE INVESTIGACIÓN

23. El protocolo de la investigación debe enviarse, para consideración, comentario, consejo y aprobación al comité de ética de investigación pertinente antes de comenzar el estudio. Este comité debe ser transparente en su funcionamiento, debe ser independiente del investigador, del patrocinador o de cualquier otro tipo de influencia indebida y debe estar debidamente calificado. El comité debe considerar las leyes y reglamentos vigentes en el país donde se realiza la investigación, como también las normas internacionales vigentes, pero no se debe permitir que éstas disminuyan o eliminen ninguna de las protecciones para las personas que participan en la investigación establecidas en esta Declaración.

El comité tiene el derecho de controlar los ensayos en curso. El investigador tiene la obligación de proporcionar información del control al comité, en especial sobre todo incidente adverso grave. No se debe hacer ninguna enmienda en el protocolo sin la consideración y aprobación del comité. Después que termine el estudio, los investigadores deben presentar un informe final al comité con un resumen de los resultados y conclusiones del estudio.

G. PRIVACIDAD Y CONFIDENCIALIDAD

24. Deben tomarse toda clase de precauciones para resguardar la intimidad de la persona que participa en la investigación y la confidencialidad de su información personal.

H. CONSENTIMIENTO INFORMADO

25. La participación de personas capaces de dar su consentimiento informado en la investigación médica debe ser voluntaria. Aunque puede ser apropiado consultar a familiares o líderes de la comunidad, ninguna persona capaz de dar su consentimiento informado debe ser incluida en un estudio, a menos que ella acepte libremente.

26. En la investigación médica en seres humanos capaces de dar su consentimiento informado, cada individuo potencial debe recibir información adecuada acerca de los objetivos, métodos, fuentes de financiamiento, posibles conflictos de intereses, afiliaciones institucionales del investigador, beneficios calculados, riesgos previsibles e incomodidades derivadas del experimento, estipulaciones post estudio y todo otro aspecto pertinente de la investigación. La persona potencial debe ser informada del derecho de participar o no en la investigación y de retirar su consentimiento en cualquier momento, sin exponerse a represalias. Se debe prestar especial atención a las necesidades específicas de información de cada individuo potencial, como también a los métodos utilizados para entregar la información.

Después de asegurarse de que el individuo ha comprendido la información, el médico u otra persona calificada apropiadamente debe pedir entonces, preferiblemente por escrito, el consentimiento informado y voluntario de la persona. Si el consentimiento no se puede otorgar por escrito, el proceso para lograrlo debe ser documentado y atestiguado formalmente.
Todas las personas que participan en la investigación médica deben tener la opción de ser informadas sobre los resultados generales del estudio.
27. Al pedir el consentimiento informado para la participación en la investigación, el médico debe poner especial cuidado cuando el individuo potencial está vinculado con él por una relación de dependencia o si consiente bajo presión. En una situación así, el consentimiento informado debe ser pedido por una persona calificada adecuadamente y que nada tenga que ver con aquella relación.
28. Cuando el individuo potencial sea incapaz de dar su consentimiento informado, el médico debe pedir el consentimiento informado del representante legal. Estas personas no deben ser incluidas en la investigación que no tenga posibilidades de beneficio para ellas, a menos que ésta tenga como objetivo promover la salud del grupo representado por el individuo potencial y esta investigación no puede realizarse en personas capaces de dar su consentimiento informado y la investigación implica sólo un riesgo y costo mínimos.
29. Si un individuo potencial que participa en la investigación considerado incapaz de dar su consentimiento informado es capaz de dar su asentimiento a participar o no en la investigación, el médico debe pedirlo, además del consentimiento del representante legal. El desacuerdo del individuo potencial debe ser respetado.
30. La investigación en individuos que no son capaces física o mentalmente de otorgar consentimiento, por ejemplo los pacientes inconscientes, se puede realizar sólo si la condición física/mental que impide otorgar el consentimiento informado es una característica necesaria del grupo investigado. En estas circunstancias, el médico debe pedir el consentimiento informado al representante legal. Si dicho representante no está disponible y si no se puede retrasar la investigación, el estudio puede llevarse a cabo sin consentimiento informado, siempre que las razones específicas para incluir a individuos con una enfermedad que no les permite otorgar consentimiento informado hayan sido estipuladas en el protocolo de la investigación y el estudio haya sido aprobado por un comité de ética de investigación. El consentimiento para mantenerse en la investigación debe obtenerse a la brevedad posible del individuo o de un representante legal.
31. El médico debe informar cabalmente al paciente los aspectos de la atención que tienen relación con la investigación. La negativa del paciente a participar en una investigación o su decisión de retirarse nunca debe afectar de manera adversa la relación médico-paciente.
32. Para la investigación médica en que se utilice material o datos humanos identificables, como la investigación sobre material o datos contenidos en biobancos o depósitos similares, el médico debe pedir el consentimiento informado para la recolección, análisis, almacenamiento y reutilización. Podrá haber situaciones excepcionales en las que será

imposible o impracticable obtener el consentimiento para dicha investigación. En esta situación, la investigación sólo puede ser realizada después de ser considerada y aprobada por un comité de ética de investigación.

I. USO DEL PLACEBO

33. Los posibles beneficios, riesgos, costos y eficacia de toda intervención nueva deben ser evaluados mediante su comparación con las mejores intervenciones probadas, excepto en las siguientes circunstancias:

- Cuando no existe una intervención probada, el uso de un placebo, o ninguna intervención, es aceptable o
- Cuando por razones metodológicas, científicas y apremiantes, el uso de cualquier intervención menos eficaz que la mejor probada, el uso de un placebo o ninguna intervención es necesario para determinar la eficacia y la seguridad de una intervención.
- y los pacientes que reciben cualquier intervención menos eficaz que la mejor probada, el placebo o ninguna intervención, no correrán riesgos adicionales, efectos adversos graves o daño irreversible como consecuencia de no recibir la mejor intervención probada.

Se debe tener muchísimo cuidado para evitar abusar de esta opción.

J. ESTIPULACIONES POST ENSAYO

34. Antes del ensayo clínico, los auspiciadores, investigadores y los gobiernos de los países anfitriones deben prever el acceso post ensayo a todos los participantes que todavía necesitan una intervención que ha sido identificada como beneficiosa en el ensayo. Esta información también se debe proporcionar a los participantes durante el proceso del consentimiento informado.

K. INSCRIPCIÓN Y PUBLICACIÓN DE LA INVESTIGACIÓN Y DIFUSIÓN DE RESULTADOS

35. Todo estudio de investigación con seres humanos debe ser inscrito en una base de datos disponible al público antes de aceptar a la primera persona.

36. Los investigadores, autores, auspiciadores, directores y editores todos tienen obligaciones éticas con respecto a la publicación y difusión de los resultados de su investigación. Los investigadores tienen el deber de tener a la disposición del público los resultados de su investigación en seres humanos y son responsables de la integridad y exactitud de sus informes. Todas las partes deben aceptar las normas éticas de entrega de información. Se deben publicar tanto los resultados negativos incluso como los positivos o de lo contrario deben estar a la disposición del público. En la publicación se debe citar la fuente de financiamiento, afiliaciones institucionales y conflictos de intereses. Los informes sobre investigaciones que no se ciñan a los principios descritos en esta Declaración no deben ser aceptados para su publicación.

L. INTERVENCIONES NO PROBADAS EN LA PRÁCTICA CLÍNICA

37. Cuando en la atención de un enfermo las intervenciones probadas no existen u otras intervenciones conocidas han resultado ineficaces, el médico, después de pedir consejo de experto, con el consentimiento informado del paciente o de un representante legal autorizado, puede permitirse usar intervenciones no comprobadas, si, a su juicio, ello da alguna esperanza de salvar la vida, restituir la salud o aliviar el sufrimiento. Tales intervenciones deben ser investigadas posteriormente a fin de evaluar su seguridad y eficacia. En todos los casos, esa información nueva debe ser registrada y, cuando sea oportuno, puesta a disposición del público.
